# Effect of face-covering use on adherence to other COVID-19 protective behaviours: A systematic review

**DOI:** 10.1371/journal.pone.0284629

**Published:** 2024-04-11

**Authors:** Adam Millest, Sidra Saeed, Charles Symons, Holly Carter

**Affiliations:** Behavioural Science and Insights Unit, UK Health Security Agency, London, United Kingdom; Addis Ababa University, College of Health Sciences, ETHIOPIA

## Abstract

During the COVID-19 pandemic, concerns were raised that face covering use may elicit risk compensation; a false sense of security resulting in reduced adherence to other protective behaviours such as physical distancing. This systematic review aimed to investigate the effect of face covering use on adherence to other COVID-19 related protective behaviours. Medline, Embase, PsychInfo, EmCare, medRxiv preprints, Research Square and WHO COVID-19 Research Database were searched for all primary research studies published from 1^st^ January 2020 to 17^th^ May 2022 that investigated the effect of face covering use on adherence to other protective behaviours in public settings during the COVID-19 pandemic. Papers were selected and screened in accordance with the PRISMA framework. Backwards and forwards citation searches of included papers were also conducted on 16^th^ September 2022, with eligible papers published between 1^st^ January 2020 and that date being included. A quality appraisal including risk of bias was assessed using the Academy of Nutrition and Dietetics’ Quality Criteria Checklist. This review is registered on PROSPERO, number CRD42022331961. 47 papers were included, with quality ranging from low to high. These papers investigated the effects of face covering use and face covering policies on adherence to six categories of behaviour: physical distancing; mobility; face-touching; hand hygiene; close contacts; and generalised protective behaviour. Results reveal no consistent evidence for or against risk compensation, with findings varying according to behaviour and across study types, and therefore confident conclusions cannot be made. Any policy decisions related to face coverings must consider the inconsistencies and caveats in this evidence base.

## 1. Introduction

The COVID-19 pandemic highlighted the important role that public behaviour can play in the control of infectious disease. Prior to the introduction of vaccination, measures to control the spread of COVID-19 consisted primarily of non-pharmaceutical interventions (NPIs) (e.g., hand cleaning; reducing contact with others; staying at home), which included the use of face-coverings [1]. Over 160 governments mandated or recommended face-coverings during the pandemic [2], and their use was seen as crucial to controlling the spread of COVID-19.

At the onset of the pandemic however, concerns were raised that the use of face coverings may provide a false sense of security, leading to lower adherence to other protective measures such as hand hygiene and physical distancing; a phenomenon known as ‘risk compensation’ [3, 4] that has its theoretical origins in both economonics and psychology. Peltzman [4], for example, proposes that each individual has a targeted level of risk. If a person feels that they have taken an action to reduce their risk in a particular context, they may feel able to take greater risks in other areas in order to maintain their targeted risk level. People therefore compensate for the perceived decline in risk by taking other risky behaviours; this is known as the Peltzman effect. For example, in the case of driving cars, the Peltzman effect predicts that safer cars will prompt riskier driving as drivers increase their driving intensity to offset their reduced risk [5]. According to this effect, face-coverings may provide a level of safety that exceeds a targeted level, and people may compensate through a reduction in other COVID-19-related safety measures. Wilde [6] predicts such behaviour through a phenomenon he refers to as ‘risk homeostasis.’ Wilde posits that, just as our bodies have a certain biological balance that we unconsciously look to maintain, psychologically we have an optimum level of risk that we also seek to maintain, and that, depending on the circumstances we find ourselves in, we will look to make risk-related adjustments. As such if we find ourselves in circumstances in which our level of risk is below this optimum level, we will look to increase it through our behaviour. In the case of face-covering use, these theories would predict that by wearing face-coverings our perceived risk of catching or transmitting a virus may decrease to below our optimum level, and so we compensate by reducing other COVID-19-related safety behaviours, such as physical distancing. In summary, though these two theories take a slightly different angle, both predict that when our behaviour or circumstances reduce the level of risk we are subjected to, we look to compensate for that risk by undertaking behaviour that has an equal and opposite effect.

The literature on risk compensation during COVID-19 has found mixed results [7, 8]. One review was carried out early in the pandemic to examine the extent to which risk compensation may affect public behaviour [9], however, given the paucity of research into COVID-19 specifically at the time, this review necessarily relied on research into risk compensation in other contexts (such as the wearing of bike or ski helmets, the use of pre-exposure prophylaxis for HIV, and the use of the HPV vaccination). In a study in Germany, Henk et al. [10] found that self-reported adherence to protective behaviours was significantly lower after the implementation of two policies: mandatory quarantine for visitors to the country and mandatory face-covering use in shops and on public transport. Similarly, Jones Ritten et al. [11] investigated the risk compensating effect of COVID-19 testing by surveying students at two large universities in the US and found a positive association between testing behaviour and the number of risky events attended (e.g., large indoor gatherings). Moreover, it was found that those who participated in the testing programme perceived that doing so reduced their risk of contracting COVID-19, and further, that there was a positive association in the perceived risk-reducing effect of the testing programme and frequency of attending risky events; that is, the more respondents believed that testing reduced their risk of contracting COVID-19, the more likely they were to attend risky events. In contrast, Ludema et al. [12], used a randomised controlled trial to investigate risk compensation and anti-body testing and found that, irrespective of whether the result was positive or negative, receiving antibody test results did not lead to significant changes in adherence to COVID-19 protective behaviours. The extent to which risk compensation has occurred during the COVID-19 pandemic is therefore unclear.

There is now a substantial body of research investigating risk compensation in relation to face-covering use in the context of COVID-19. The aim of this systematic review was therefore to identify, summarise and assess the findings from studies examining the effect of face covering use on adherence to other protective behaviours in the context of COVID-19.

## 2. Method

Details of the protocol for this systematic review were registered on PROSPERO before screening took place. The protocol’s number is CRD42022331961 and can be accessed at https://www.crd.york.ac.uk/PROSPERO/display_record.php?RecordID=331961 [13]. It can also be seen in S1 Appendix.

### 2.1 Eligibility criteria

Studies were eligible if they reported primary quantitative or qualitative research relating to the impact of face covering use on adherence to other protective behaviours during the COVID-19 pandemic. Protective behaviours of interest included but were not limited to: COVID-19 physical/social distancing, hygiene (e.g., avoidance of face-touching, regular handwashing), staying at home, and reducing travel. Studies that examined the effectiveness and/or efficacy of wearing face coverings or factors related to adherence to face covering use were excluded. Published research and pre-publication articles were included. Reviews, position/discussion papers, conference abstracts, protocol papers, modelling studies, case reports and studies published in languages other than English were excluded (see Table 1).

**Table 1:** Inclusion criteria.

Inclusion Criteria	Exclusion Criteria
Papers published from 1^st^January 2020 to present day at time of search (17^th^ May 2022) All populations and all public settings other than healthcare settings Quantitative or qualitative research relating to the impact of face covering use on adherence to other protective behaviours during the COVID-19 pandemic Face covering use: i. All types of face covering, including (but not limited to) handmade and commercial cloth coverings (cloth, cotton, gauze, etc), and medical coverings such as surgical face masks. ii. Face covering mandate. Experimental studies, observational studies, qualitative studies, laboratory studies, any other primary data, secondary data analyses. Published research and pre-publication articles	Healthcare settings Papers not in English Systematic reviews, narrative reviews, guidelines, position/discussion papers, conference abstracts, protocol papers, modelling studies and case reports. Studies which examined the effectiveness and/or efficacy of wearing face coverings or factors related to adherence to face covering use

### 2.2 Search strategy

A systematic search was conducted by UKHSA researchers on 17^th^ May 2022 for papers from January 2020 until the date of searches. Sources searched included Ovid Medline, Ovid Embase, Ovid PsychInfo, Ovid EmCare, medRxiv preprints, Research Square and WHO COVID-19 Research Database. Search terms included terms related to COVID-19 (e.g., COVID-19, coronavirus, Sars-COV2), face coverings (e.g., mask, face cover, mouth covers) and protective behaviours (e.g., social distancing, handwashing, face-touching). A complete list of search terms is available in S2 Appendix.

Forward and backward citation searches of included papers were also conducted on 16^th^ September 2022, and eligible papers from 1^st^ January 2020 to that date were also included.

### 2.3 Study identification

**Fig 1:**
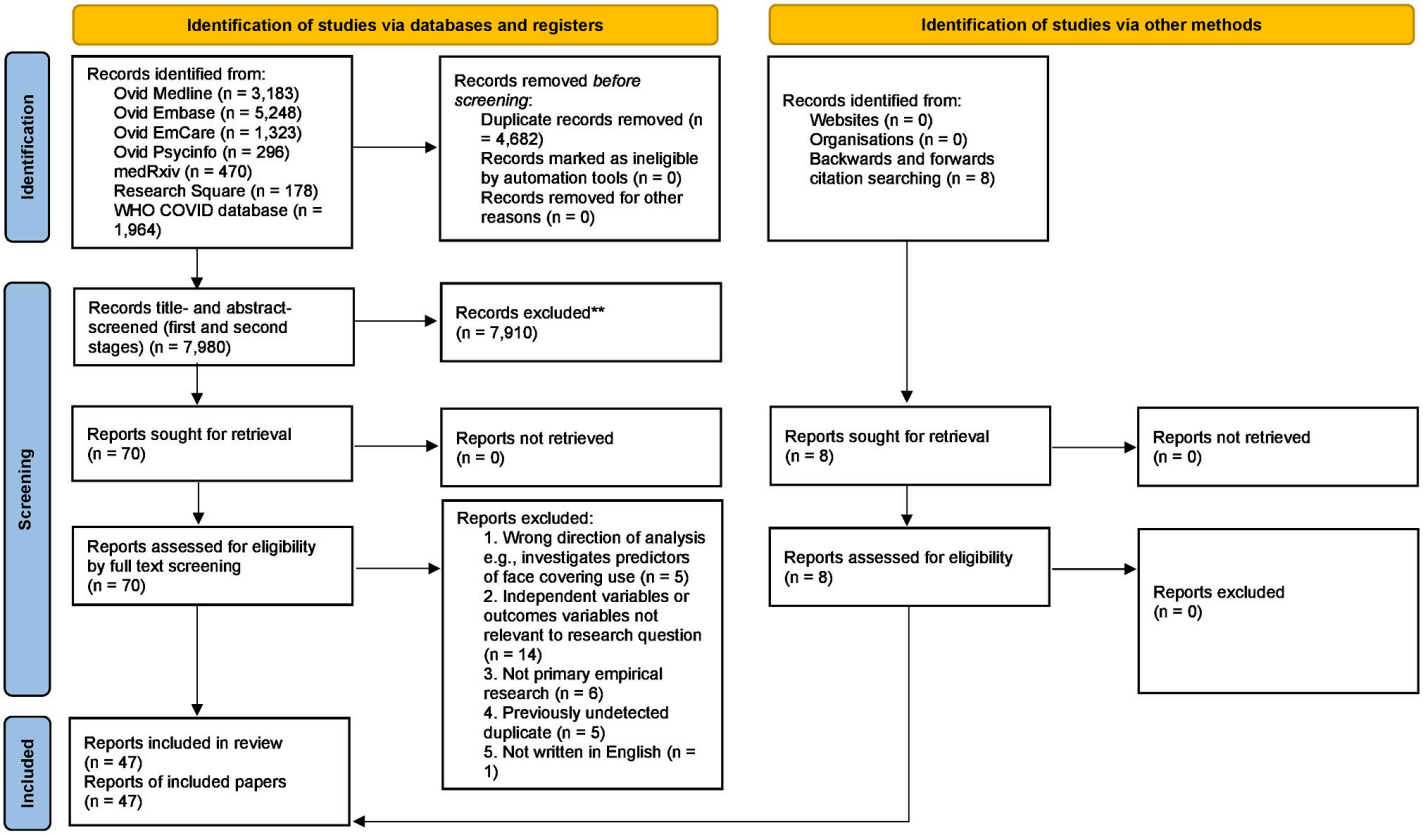
PRISMA 2020 flow diagram of the identification of studies process.

Selection and screening of papers followed a systematic search method following a Preferred Reporting Items for Systematic Reviews and Meta-Analyses (PRISMA) framework [14]. The study identification process is detailed in Fig 1 and contains full details of papers included and excluded at each stage. Papers were initially title- and abstract-screened (decisions were made as to the eligibility of each paper following a review of their title and abstract alone). In the first stage of title and abstract screening, 10% of records were assessed in duplicate by two reviewers, and disagreements were resolved by discussion and consensus. In the second stage of title and abstract screening one reviewer assessed the remaining 90% of records. The screening tool Rayyan [15] was used for both these first and second stages. All relevant records were then screened by full text by one reviewer (decisions were made as to the eligibility of each paper following a review of the paper in full). These decisions were then checked by a second reviewer. There were five reasons for exclusion: (i) The direction of analysis was incorrect e.g., the study investigated behavioural predictors of face-covering use rather than vice versa (n = 5), (ii) the independent variables or outcome variables were not relevant to the research question (n = 14), (iii) the study did not comprise primary empirical research (n = 6), (iv) the paper was a previously undetected duplicate (n = 5), and (v) the paper was not written in English (n = 1). By the end of this process, 47 papers were deemed eligible and included in the review.

### 2.4 Data extraction and synthesis

Findings related to any relevant COVID-19 protective behaviours were extracted; at the completion of extraction, these comprised the following behavioural outcomes: physical distancing, mobility, face-touching, hand hygiene, close contacts, and generalised measures of protective behaviour. All results from selected papers were included. The following information was then collated for each study: country, publication status, population and sample size, methods, key outcome variables and main results. Data were initially collated into one table which included an outcome variable column. This table was then divided by outcome variable into six smaller data extraction tables, and a narrative summary of results was produced, structured by these six outcome variable categories.

Data extraction was completed for each included study by one reviewer and independently checked by a second reviewer, with discrepancies resolved by discussion. Any evidence that was not directly relevant to the review question was not extracted.

### 2.5 Quality assessment

Quality of the selected articles, including risk of bias, was evaluated by one reviewer using the Quality Criteria Checklist (QCC) tool which can be used to assess the methodological quality of a study [16]. Using the QCC, studies were characterized as high, medium, or low quality.

This checklist tool is composed of ten questions, 4 of which are considered critical (questions on selection bias, group comparability, description of exposure/assessment of transmission routes, and validity of outcome measurements); full details can be seen in S3 Appendix.

A study was rated as high methodological quality if the answers were yes to the four critical questions plus at least one of the remaining questions. A study was rated as low methodological quality if answers were no to more than half of the 10 questions. Otherwise, a study was rated as medium methodological quality. In line with a systematic review that found that self-report methods of capturing adherence to protective behaviours can over-estimate objectively observed adherence by up to a factor of five [17], any studies which employed a self-report methodology were deemed not to satisfy the validity of outcome criterion, and so were necessarily limited to a quality score of medium or below. An additional reviewer independently assessed five of the studies, and conflicting findings were discussed. Following discussions, a potential source of bias was identified in the first reviewer’s application of one of the tool’s questions, and all studies were subsequently re-evaluated to correct for this bias.

## 3. Results

### 3.1 Overview of search results

The initial searches generated 12,662 articles. After de-duplication, 7,980 records remained, and title and abstract screening resulted in 70 papers being accepted for full text screening. Following full text screening, 39 papers were accepted for inclusion in the review. Backwards and forwards citation searches were conducted that yielded a further 8 papers for inclusion, meaning the total number of papers included in the review was 47. Quality appraisal of the articles revealed that nine were of a high quality, 33 of a medium quality, and five of a low quality. Of the papers included nine were pre-prints, 35 were peer-reviewed publications, and three were published conference papers. Finally, it was found that some papers included more than one methodology, intervention, or protective behaviour and thus, the number of studies described for each of these categories may exceed the total number of papers.

All studies included used quantitative methods, and none used qualitative methods. Methods included field experiments (n = 7), natural experiments (n = 14), lab experiments (n = 14), observational studies (n = 10) and cross-sectional studies (n = 9). Of these, 30 studies measured behaviour through observation, and 22 measured behaviour through self-report. Of the self-report studies, 10 were concerned with current or previous behaviour, and 12 with expected or intended behaviour. Though all included papers necessarily investigated the effect of face covering use on protective behaviours, the exact intervention of focus in each case was varied and fit into one of five categories: face covering use (self) i.e., the act of wearing a face covering oneself (n = 21); face covering use (other) i.e., in some way interacting with someone else wearing a face covering (n = 18); face covering use (self and other) i.e., in some way interacting with someone else who is wearing a face covering whilst also wearing a face covering oneself (n = 1); face covering policy i.e., the implementation of a face covering mandate (n = 19); and face covering intervention package i.e., a community programme designed to increase uptake of face covering usage (n = 1). The protective behaviour(s) in each case also varied, and in each case fit into one of 6 categories: physical distancing i.e., interpersonal distance (n = 30); mobility i.e., the extent to which people leave their home and visit public or residential spaces (n = 13); face-touching (n = 7); hand hygiene e.g., hand washing, avoidance of handshakes (n = 5); close contacts i.e., the extent to which people have close and extended interactions with people outside of their household (n = 4); and generalised protective behaviour i.e., a single measure to encapsulate two or more COVID-19 protective behaviours other than face-covering use, e.g., hand-washing, physical distancing and staying at home (n = 2). Full details on each paper can be seen in the data extraction tables in S4 Appendix.

Some studies initially appeared to meet the inclusion criteria but were ultimately excluded. For example, one study investigated how face covering use affected perceptions of physical distance between oneself and another person, however, since it did not investigate how face covering use affected distancing behaviour, it was not deemed to be eligible for inclusion [18]. Similarly, another study investigated participants’ judgements on how face covering use moderates the relationship between a given physical distance and transmission risk and was not included since, although its focus was related to the review’s objectives, it did not directly address it, in that it did not investigate how face covering use directly impacted adherence to protective behaviours [19].

The section below presents findings in relation to each of these six behaviours and describes the extracted data in terms of the effect that face covering use has on each type of behaviour, also exploring any patterns with regards to how the effect of face covering use varies by the type of intervention, and the type of study design.

### 3.2 Physical distancing (see table 2a in S4 Appendix for full details)

Outcomes in relation to the impact of face covering use on physical distancing were mixed and varied depending on the type of study. Findings from the majority of lab experiments (10 of 13) indicated that physical distancing was lower in conditions where a face covering was used by another person compared with conditions where it was not [20–27], [28: two different studies], and two of these studies also found that physical distancing was lower when a face covering was used by the self [28: two different studies]. Additionally, one of these studies investigated how physical distancing in conditions with and without face covering use compared with a baseline measure (that is, physical distancing outside the context of face covering use) [20]. It was found that physical distancing was significantly greater than the baseline in conditions without face covering use, but not significantly different in conditions with face covering use, potentially suggesting that differences between conditions is driven by participants moving away from people without face coverings (relative to where they would normally position themselves), rather than towards people with face coverings. Of the remaining three studies, one found that physical distancing was lower in conditions of face covering use (other), but that this effect was conditional on situational factors such as effort required to maintain physical distance, and age of the other person [29], and two found that distancing was greater in the case of face covering use (other) [30], [31: Study 2].

In contrast, the majority of field experiments (5 of 6) found that physical distancing was either greater or not significantly different in conditions where face coverings were used, compared to conditions where they were not. Two studies found that physical distancing was greater in conditions where face coverings (self or other) were used compared to when they were not [31: Study 1], [32], with a third study finding that physical distancing was greater in areas where a face covering intervention package had been implemented compared to those where it had not [33]. One study found mixed effects: face covering use (self) had no effect on distancing, but distancing was greater in conditions of face covering use (other) [34]. Another study found no differences in distancing between conditions of face covering use (self and other) and conditions without face covering use [35]. A minority of field experiments (1 of 6) found that physical distancing was lower in conditions of face covering use (other), but that this effect was conditional on gender of participant, race, and social status of confederate (a member of the research team acting as a participant), and face covering policy implemented at the time [36]. Additionally, three of the above studies also investigated through a natural design whether the effect of face covering use on physical distancing differed between different time periods that varied by the face covering policy (mandatory or voluntary) implemented at the time. One study found that face-covering use by others significantly reduced physical distancing adherence amongst men only, both in mandatory and in voluntary face covering policy contexts; the effect was slightly more pronounced in the mandatory context [36]. The two other studies found no difference between policy contexts; both found that face covering use (other) increased physical distancing both when it was mandated and when it was voluntary [32, 34].

The four natural experiments explored the impact of face covering policy on physical distancing. Three of these found that physical distancing did not significantly differ according to whether face covering wearing was voluntary or mandatory [28: Study 2], [34], [37: Study 2], while a third found that the impact of face covering policy on physical distancing was dependent on context; attention given to physical distancing decreased when a face covering mandate was introduced on public transport, but increased when the mandate was expanded to bars and restaurants, and again when expanded to all public spaces [38].

The four observational studies found either a positive or a neutral relationship between face covering use or face covering policy and physical distancing. Three observational studies found no significant relationship between face covering use (self) or face covering policy and physical distancing [37: two studies], [39], while the fourth observational study found that physical distancing was greater in those who wore face coverings, and also that it was greater when a face covering mandate was implemented [40].

Four of the six correlational studies found that as self-reported face covering use (self or other) increased, so did self-reported physical distancing [41–44]. One study showed mixed results for the relationship between face covering use and physical distancing, finding that those who reported wearing face coverings all the time were more likely to report adherence to physical distancing compared with those who reported never doing so, but those who reported wearing face coverings sometimes were more likely to report having reduced distancing with people than those who reported never doing so [45]. The final correlational study found no relationship between face covering use (self) and physical distancing [46].

### 3.3 Mobility (see table 2b in S4 Appendix for full details)

Of the 10 natural experiments, five found that when face covering policies were in place mobility increased (i.e., people were less likely to stay at home) [47–51], four found that mobility was unchanged [52–55], and one found that mobility decreased (i.e., people were more likely to stay at home) [56]. The only lab experiment found that mobility is likely to increase when policies are in place, as participants reported being more willing to use the London Underground when a face covering mandate was in operation [57].

Of the two cross-sectional studies, one found that self-reported adherence to personal protective behaviours, which included face covering use (self), predicted self-reported frequency of going out in public, and that this relationship was mediated by belief in ‘substitution myths’; the belief that one protective behaviour can be substituted for another, with risk staying constant [46]. The second cross-sectional study found that those who reported always wearing a face covering outside their home were less likely to report visiting a friend, neighbour, bar or club, compared with those who did not report always wearing a face covering outside their home [44].

### 3.4 Face-touching (see table 2c in S4 Appendix for full details)

The only field experiment found that face covering use (self) resulted in less face-touching; there was a significant increase in the number of individuals who touched their eyes or hair when they were not wearing a face covering, and, when investigating areas not covered by a face covering (hair, forehead, eyes, and ears) the absolute number of total touches was significantly higher in those not wearing one [58]. Two of the six observational studies found similar results; those who wore face coverings were less likely to touch their face [59, 60]. Two more observational studies found that results were dependent on the operationalisation of face-touching; when face-touching was defined as making contact with any part of the face (including touches to the mask), there was no significant relationship between face covering use (self) and face-touching, but when it was defined as making contact with specific parts of the face (not including touches to the mask), those who wore face coverings touched their faces less frequently [61: two studies]. A minority of observational studies (1 of 6) found a greater frequency of face-touching in those who wore coverings compared with those who did not [62]. The final observational study found that face covering policy had no impact on face-touching; there was no statistically significant association between the face covering policy in operation and frequency of face-touching [63].

### 3.5 Hand hygiene (see table 2d in S4 Appendix for full details)

The only natural experiment found that face covering policy had no impact on hand hygiene; attention paid to hygiene practices was not significantly different according to the implementation of different face covering policies (38). The three cross-sectional studies found that those who reported wearing face coverings more often were more likely to report also adhering to hand hygiene practices [41, 44, 45].

### 3.6 Close contacts (see table 2e in S4 Appendix for full details)

One of the two natural experiments found that the number of close contacts a person had was significantly lower when a face covering mandate was expanded from public transport to include restaurants and bars, and lower again when expanded to all public spaces [38]. The other natural experiment found that a face covering mandate did not affect whether participants cancelled or postponed personal or social activities [55].

One of the three cross-sectional studies found that the relationship between face covering use (self) and number of close contacts was mixed; those who reported wearing face coverings all the time were more likely to report spending less than 15 minutes in close contact with someone compared with those who reported never wearing face coverings, while those who reported sometimes wearing face coverings were more likely to report spending over 60 minutes in close contact with someone compared with those who reported never doing so [45]. The second cross-sectional study found that those who did not always wear face coverings were more likely than those who always wore face coverings to have close contact with people they do not live with [44], whilst the final cross-sectional study found that workers who self-reported wearing a face covering outside of work reported more daily contacts than those who did not wear a mask outside work [64].

### 3.7 Generalised protective behaviour (see table 2f in S4 Appendix for full details)

The term ‘generalised protective behaviour’ was used to categorise any studies whose outcome variable was a single measure which encapsulated two or more other COVID-19 protective behaviours besides face covering use e.g., hand washing, physical distancing and staying at home. Two studies examined generalised protective behaviour, and both found that self-reported face covering use (self) was associated with self-reported behaviours that are likely to reduce the spread of COVID-19. In one study the majority of participants reported that their adherence to other protective behaviours was either unchanged or greater when they wore a face covering compared with when they did not [65], while the other study found that self-reported face covering use (self) was associated with self-reported reduced engagement with COVID-19 related risky behaviour [66].

### 3.8 Quality assessment

Few clear patterns were found when analysing the results of studies cross-sectionally by quality. With the exception of face-touching, for all behaviours (physical distancing, mobility, hand hygiene, close contacts and generalised protective behaviour), there was no apparent association between quality of the study and the results. For face-touching, studies were either of a low or medium quality, with all four studies of medium quality finding the same result: a negative effect of (or association with) face covering use. The three studies of low quality were mixed: one found a positive association, one found a negative association, and one found a nonsignificant association.

## 4. Discussion

Concerns have been raised about the potential impact of face covering use on adherence to other COVID-19 protective behaviours. While several studies that explore this issue have been conducted, they had not previously been brought together in a review. This systematic review has identified and summarised the findings of 47 papers that collectively investigate the effect of face covering use on other COVID-19-related protective behaviours, namely, physical distancing, mobility, face-touching, hand hygiene, close contacts and generalised protective behaviour. Findings showed that results varied considerably, and that the nature of the variation often depended upon the specific behaviour investigated and the type of study employed to investigate it. The sections below, organised by behaviour, summarise and discuss the findings.

### 4.1 Physical distancing

One of the clearest patterns emerging from the studies relating to physical distancing was the distinction between the respective findings of lab experiments and field experiments. The majority of lab experiments found that physical distancing was lower in conditions of face covering use (compared with conditions without face covering use), whereas the majority of field experiments found that physical distancing was either greater or unchanged in conditions of face covering use.

A number of considerations should be made when interpreting these results, starting with the respective merits of field and lab experiments. There are three main advantages of lab experiments. First, the majority of the lab experiments employed a within-subjects design, meaning that individual differences that may impact physical distancing (e.g., age, gender, risk perception, conscientiousness) are essentially controlled for in a way that cannot be done with between-subjects field experiments [67] (although estimates of such variables, e.g., age, were in some cases recorded and controlled for in the field experiments). Second, the lab experiments typically allowed for more precision in data collection. The majority of lab experiments used an on-screen slider for participants to indicate their preferred distances, or a virtual reality environment in which computers captured the distances participants maintained from virtual agents. In contrast, some of the field experiments employed human judgement to determine whether a physical distancing guideline had been violated, increasing the risk of bias. However, four of the six field experiments used some form of electronic sensor to collect data (all of which found that physical distancing was greater or unchanged in conditions of face covering use). Third, lab experiments are able to examine the impact of face covering use on physical distancing in a context which is agnostic of face covering policy (mandatory vs voluntary), in contrast to field experiments which are necessarily conducted under periods of either mandatory or voluntary face covering wear (the potential for an interaction between face covering use and face covering policy is discussed in more detail in subsection 4.7).

However, whilst field experiments are less controlled than lab experiments, they are more ecologically valid [68]. A key advantage of field experiments compared to lab experiments is that the disease transmission risk is real, not hypothetical; therefore, a person’s approach to physical distancing has a real impact on their own risk and on others’ risk of contracting and spreading COVID-19. In all but one of the six field experiments not only were participants blinded to the experimental condition they were assigned to, but they were also unaware that they were taking part in an experiment at all, and so responses were unlikely to be biased by, for example, demand characteristics. Behaviour captured in field experiments may therefore be more representative of, and generalisable to, wider populations than that collected during lab experiments, in which participants could second-guess research objectives, potentially causing demand effects [67]. Additionally, in the majority of lab experiments, rather than being measured for actual behaviour, participants were asked to indicate distances they would prefer from the avatars or characters, allowing them to make a conscious decision which may or may not be indicative of how they might behave in real-life scenarios.

In addition to the lab and field experiments, the effect of face covering use on physical distancing was also investigated through a number of different methodologies; the majority of natural and observational studies tended not to find any significant associations between face covering use (or face covering policy) and physical distancing, and cross-sectional studies tended to find positive associations between self-reported face covering-wearing and physical distancing. These different study types also have their respective strengths and weaknesses that should be considered when interpreting the results. The observational studies are necessarily high in ecological validity, however, are less controlled [69]. As such, the means of measurement (human judgement) may be lacking in precision and subject to bias, and furthermore, only claims of association (and not causality) can be made [70]. For example, it is likely that relationships between face covering wearing and physical distancing are driven by confounding variables (e.g., risk perception, age, conscientiousness) impacting both behaviours. The natural experiments, though again high in validity, are types of observational studies and so are subject to the same qualifications. Cross-sectional studies are also unable to draw claims of causality due to the likely impact of confounding variables [70] and may also be limited in their precision since measures are self-reported and not necessarily indicative of real-life behaviour [17].

A final consideration that should be made when interpreting the results is to consider the nature of the intervention in each case. Specifically, consideration should be given to: whether the independent variable (IV) is face covering condition of the self, face covering condition of another person, face covering policy, or some combination of any of the three; and the way in which physical distancing is operationalised in each case. Whilst there is no clear pattern with regards to how these different interventions and operationalisations of distancing impact outcomes, it is important to consider such differences when interpreting findings. Overall, it is clear that there is no consensus in relation to the impact of face covering use on physical distancing. Further research is therefore required to bring the merits of different study types together and capture more reliable, and perhaps more consistent, findings with regards to the effect of face covering use on physical distancing.

### 4.2 Mobility

The findings on mobility are also inconclusive, although there is more evidence to suggest that face covering policies increase mobility than there is to suggest that they decrease mobility. Natural experiments tended to compare changes in mobility from a baseline between areas and periods of time that varied according to the face covering policy implemented. The findings arising from such studies are therefore likely to be confounded by any number of other variables which are likely to impact upon mobility (e.g., other COVID-19 public health measures, rate of infection at time/area of data collection) and claims of causality must be made with caution. Generally speaking, however, these studies employ rich data sources and complex statistical models to account for these variables as far as is possible, and so conclusions made from these studies can be arrived at with a certain level of confidence. If it is the case that implementing face covering policies increases mobility, a plausible explanation may be that a mandate instils confidence in people that if they are to leave home others are more likely to wear a face covering, leading to a greater sense of security; this is therefore a potential example of the Peltzman Effect [4]. Further qualitative work could be conducted to investigate how face covering policies impact upon people’s decision to leave home and move around their communities.

Two cross-sectional self-report studies which investigated how face covering use by the self impacted mobility were inconclusive, with relationships found in both directions [44, 46]. Interestingly however, in one case it was found that a positive relationship between self-reported adherence to personal protective behaviour (including wearing a face covering) and going out in public with people outside ones’ household was mediated by a belief that one protective behaviour can be safely substituted for another [46], providing evidence in favour of the risk compensation hypothesis.

### 4.3 Face-touching

The impact of face covering use on face-touching was the most consistent of all the behavioural outcomes, with a general trend for face-touching to be lower in conditions of face covering use than in conditions without. This result was found in a field experiment [58] and four of six observational studies investigating the relationship between face covering use by the self and face-touching [59, 60], [61: two studies]. Whilst observational studies are subject to the same caveats described in the physical distancing subsection the general pattern of a negative relationship between face touching and face covering use suggests that even if risk compensation is at play, it is not strong enough to result in a positive relationship. Furthermore, that this pattern is corroborated by the experimental study boosts the claim that face covering use might reduce face touching behaviour. Additionally, all of the relatively higher-quality studies investigating face-touching (which were all of a medium, as opposed to a low quality) found face covering use to have a negative effect on (or a negative association with) face-touching. It should however be noted that different operationalisations of face-touching were employed across face-touching studies, and that in cases where significant effects or associations were found, they did not always apply to all parts of the face. Further research using experimental designs is required in this area, but on the basis of the studies reviewed to date, it seems reasonable to conclude that face covering use reduces instances of face-touching.

### 4.4 Hand hygiene

Across four studies (one natural experiment and three cross-sectional studies), three studies found a positive relationship between either face-covering use or face-covering policy and hand hygiene practices and a fourth found no significant relationship. The three studies which found positive relationships were cross-sectional [41, 44, 46] and based on self-report surveys; findings therefore show that those who report wearing face coverings are more likely to also report practicing good hand hygiene, but the cross-sectional nature of the studies means a causal relationship cannot be established. Furthermore, these studies can only inform us on how the isolated practices of wearing a face covering and practising hand hygiene relate to one another and cannot tell us whether hand hygiene is affected whilst wearing a face covering. The natural experiment [38], which employed face covering policies as an IV and found no significant effect on self-reported attention paid to hygiene practices may be more informative, suggesting that implementing a face covering mandate has no ‘collateral’ effect on hand hygiene practices. It should be noted however that the natural experiment is also subject to the caveats around accuracy associated with self-report data. In summary, the literature on the effect of face covering use on hand hygiene is limited and relies on self-report data, possibly because hand hygiene is a relatively private practice that is difficult to investigate using an experimental or observational design. On the basis of the literature reviewed however, it can be said that there is no evidence thus far to suggest that face covering use causes a reduction in hand hygiene practices.

### 4.5 Close contacts

There was little consensus in the literature on close contacts. Across two natural experiments, it was found that face covering mandates either had no effect on close contacts, or that they reduced them [38, 55]. Across three cross-sectional studies the results were mixed with both positive and negative relationships found [44, 45, 64]. It is therefore difficult to draw robust conclusions about the impact of face covering use on close contacts.

### 4.6 Generalised protective behaviour

Two cross-sectional studies [65, 66] investigated the relationship between face covering use by the self and generalised adherence to other protective behaviours and found a relationship between face covering use and increased adherence to other protective behaviours (or reduced undertaking of risky behaviours). As previously discussed, cross-sectional studies do not allow for claims of causality to be made, and confounding variables are likely to influence any relationships.

### 4.7 Risk compensation outcomes under different policies

An area for consideration is whether risk compensation behaviour varies according to whether face covering use is voluntary or mandatory. For example, it might be expected that when face covering use is voluntary, there is a stronger relationship between face covering use (i.e., self-selected face covering use) and adherence to other protective behaviours given that the decision to wear a face covering is more likely to be driven by one’s own risk perception rather than the extrinsic factor of the mandatory face covering policy. Equally, when face covering use is voluntary, their use may serve as a stronger social signal as to the preferences or condition of the person using them since others know that they are doing so despite the lack of a mandate. For example, voluntary use may signal that the user is particularly risk-averse and therefore others may be more likely to keep their distance out of consideration and respect. Alternatively, voluntary use may signal that the user is more likely to be infectious, and so others may be more likely to keep their distance for self-protective reasons.

In the present review, only three included studies [32, 34, 36] compared the effect of face covering use across different face covering policies (mandatory or voluntary) and only through a natural design and for one protective behaviour (physical distancing). In all three studies, the effect of face covering use by others on physical distancing adherence was not influenced by policy context, although in one study the effect was reportedly slightly more pronounced in the mandatory than in the voluntary context. There were also 17 studies in which, although the authors did not assess impact of face covering policy, they did provide information on the real-world policy implemented during the time of study; that is whether face covering use was mandatory, voluntary or whether the policy was varied (i.e., that for some of the duration of the study it was mandatory, and for some of the time it was voluntary). Of the 17, thirteen studies focused on physical distancing (five carried out in a mandatory face covering context, six in a voluntary face covering context, and two in contexts where the policy varied between the two) and results across studies were mixed in all three contexts [28: two studies, 29, 31: two studies, 33, 35, 37, 40, 41, 44–46]. Two focused on mobility, and in both cases face covering use was voluntary but a positive association was found in one [46] and a negative association in the other [44]. There were three studies focused on face-touching [58, 59, 61: study two] and three on hand hygiene [41, 44, 45]. For both protective behaviours face covering policy varied across studies but the face-touching studies all reported negative effects or associations and the hand hygiene studies all reported positive effects or associations. Two studies were focused on close contacts, one of which was conducted under mandated face covering use and found the association to be equivocal [45], and one of which was conducted when face covering use was voluntary and found the association to be negative [44]. A final study that focused on generalised protective behaviour was conducted under varied policies and found a positive association [65].

It is difficult to draw robust conclusions about any interaction between face covering use and face covering policy from studies included in this review. A particular limitation of the literature is that no study to date has applied type of face covering policy (mandatory or voluntary) as an independent variable to assess its interaction with face covering use on risk compensation for other protective behaviours. This is an evidence gap that should be addressed in future research.

### 4.8 Limitations

This review is novel in drawing together current literature on the impact of face covering use on other protective behaviours during the COVID-19 pandemic and contributing to an understanding of risk compensation in this context. However, some limitations should be acknowledged when considering this review’s findings. First, despite every attempt to apply the inclusion and exclusion criteria systematically and objectively, it is likely that some level of subjectivity may have impacted on paper screening and selection. To mitigate against this, 10% of the title and abstract screening was undertaken by two researchers, and any disagreements resolved by discussion and consensus. The full-text screening was also checked by a second researcher. Second, due to resource constraints (namely, the linguistic limitations of the researchers), only papers published in English were included in the review, inevitably creating a geographical bias. For example, 32 of the 47 papers described studies carried out in the UK, Western Europe, Canada, North America, or Australia. An additional consideration relates to the potential to conduct a meta-analysis of the included studies. Given the heterogeneity of the included studies (both in terms of clinical heterogeneity and methodological heterogeneity), a decision was taken not to carry out a meta-analysis. This is in line with Cochrane guidance on heterogeneity within meta-analysis, which states that meta-analysis should only be undertaken if studies are considered to be sufficiently similar to ensure a meaningful outcome and avoid drawing potentially misleading conclusions. However, as further research is conducted in this area, potentially resulting in more clinically and methodologically similar studies, future studies could build on this review by carrying out a meta-analysis.

### 4.9 Conclusion

Overall, findings relating to the impact of face covering use or policy on other protective behaviours are inconsistent, varying both according to the particular behaviour in question, and by study type. For some behavioural outcomes, it is possible to draw tentative conclusions regarding the impact of face coverings on behaviour. For example, evidence suggests that wearing a face covering reduces the amount that an individual touches their face; face coverings may therefore have a positive impact on face touching behaviours. Conversely, evidence suggests that the introduction of face mask mandates may increase mobility; face coverings may therefore have a negative impact on mobility-related behaviours. Some studies suggest that there may be a relationship between wearing a face covering and other protective behaviours (such as increased hand hygiene). However, these studies are predominately cross-sectional and rely on self-report measures; they therefore do not allow conclusions to be drawn about the impact of face covering use on adherence to these behaviours. Evidence relating to the impact of face covering use on physical distancing and close contacts is mixed. Findings are particularly inconsistent in relation to physical distancing, with considerable discrepancy found between study types. Whilst assessments of behaviour in real-life settings tend to find that face covering use either increases or has no impact upon physical distancing, lab experiments tend to suggest that physical distancing decreases in conditions of face covering use. There is some suggestion that the impact of face covering use on physical distancing could be affected by whether or not mandatory face covering policies are in effect (which would indicate that conclusions drawn from a voluntary context should not be directly applied to a mandatory context and vice versa) but further assessment of this potential moderating effect is warranted. Evidence relating to the potential impact of risk compensation is also inconsistent; while some studies suggest risk compensation may play a role in shaping behaviour, others suggest it has no impact.

Overall, this review highlights that the impact of face covering use on other protective behaviours is likely to vary based on the behaviour under investigation. While for some behaviours the evidence suggests a positive impact (e.g., face coverings may result in reduced face touching), for other behaviours the evidence suggests a negative impact (e.g., increased mobility), or provides no clear consensus (e.g., physical distancing, close contacts, and adherence to other protective behaviours). There are two key recommendations that can be made from this review: 1) further research, using more robust research designs, is needed to establish whether face covering use affects different types of protective behaviours; and 2) any policy decisions made in relation to recommended or mandatory face covering use must take into account the inconsistencies and caveats in the evidence base, recognising that current evidence does not allow firm conclusions to be drawn about the potential impact of face covering use on adherence to other protective behaviours. Recommendations for future research are summarised below.

#### 4.9.1 Recommendations for future research

- Further field experiments (or more ecologically valid lab experiments) investigating the effect of face covering use on physical distancing. Such studies might make a particular focus of investigating:
  - Differing effects of face covering use by the self and face covering use by another person
  - How different face covering policies might moderate the relationship between face covering use and physical distancing. For example, a field experiment could be conducted whereby two manipulations occur; one whereby the setting varies according to the face covering policy implemented (mandatory or voluntary), and one whereby a confederate appears either wearing or not wearing a face covering. Physical distancing from the confederate would then be measured. This would allow for the direct comparison between the moderating effects of voluntary and mandatory settings with more control over extraneous factors.
  - How effects might vary according to the operationalisation of distancing; for example, whether the absolute distance is measured, or whether a binary variable capturing whether or not a particular distance or guideline is adhered to is captured
- Lab experiments replicating those cited in this review, but that vary by the framing of physical distancing (i.e., self-protective or prosocial)
- Qualitative research investigating how differing face covering policies affect attitudes towards leaving the home and moving around the community
- Experimental studies, including lab, natural, or field experiments, investigating the effect of face covering use and face covering policies on face-touching, hand hygiene and close contacts

## Supporting information

S1 Appendix Protocol. (PDF)

S2 Appendix Search strategies. (XLSX)

S3 Appendix Quality criteria checklist. (PDF)

S4 Appendix Data extraction tables. (DOCX)

S5 Appendix PRISMA 2020 checklist. (DOCX)

S6 Appendix PRISMA 2020 abstract checklist. (DOCX)
