# Supplementary material for: Effect of face-covering use on adherence to other COVID-19 protective behaviours: A systematic review
Source: PLoS One. 2024 Apr 11;19(4):e0284629. doi: 10.1371/journal.pone.0284629 (PMC11008824; doi:10.1371/journal.pone.0284629)
Supplement: Attachment — Submitted filename: PONE-D-23-08549_R1.pdf [file pone.0284629.s008.pdf]

PLOS ONE

Effect of face-covering use on adherence to other COVID-19 protective behaviours: a systematic review

--Manuscript Draft--

**Manuscript Number:**	PONE-D-23-08549R1
**Article Type:**	Research Article
**Full Title:**	Effect of face-covering use on adherence to other COVID-19 protective behaviours: a systematic review
**Short Title:**	Face-coverings and COVID-19 risk compensation
**Corresponding Author:**	Holly Carter, BSc (HONS) Psychology<br>Public Health England<br>Salisbury, UNITED KINGDOM
**Keywords:**	face coverings; face masks; COVID-19; risk compensation; protective behaviours; non-pharmaceutical interventions; physical distancing; face-touching; hand hygiene
**Abstract:**	During the COVID-19 pandemic, concerns were raised that face covering use may elicit risk compensation; a false sense of security resulting in reduced adherence to other protective behaviours such as physical distancing. This systematic review aimed to investigate the effect of face covering use on adherence to other COVID-19 related protective behaviours. Medline, Embase, PsychInfo, EmCare, medRxiv preprints, Research Square and WHO COVID-19 Research Database were searched. All primary research studies published from 1st January 2020 to 17th May 2022 which investigated the effect of face covering use on adherence to other protective behaviours in public settings during the COVID-19 pandemic were included. Papers were selected and screened in accordance with the PRISMA framework. Backwards and forwards citation searches of included papers were also conducted on 16th September 2022, with eligible papers published between 1st January 2020 and that date being included. A quality appraisal including risk of bias was assessed using the Academy of Nutrition and Dietetics' Quality Criteria Checklist. This review is registered on PROSPERO, number CRD42022331961. 47 papers were included, with quality ranging from low to high. These papers investigated the effects of face covering use and face covering policies on adherence to six categories of behaviour: physical distancing; mobility; face-touching; hand hygiene; close contacts; and generalised protective behaviour. Results reveal no consistent evidence for or against risk compensation, with findings varying according to behaviour and across study types. There is a suggestion that face covering use might reduce face-touching and face covering mandates might increase mobility, though the lack of robust evidence means these are tentative claims. Evidence on the other protective behaviours is largely inconsistent, and therefore confident conclusions cannot be made in these areas. Any policy decisions related to face coverings must consider the inconsistencies and caveats in this evidence base.
**Order of Authors:**	Adam Millest
	Sidra Saeed
	Charles Symons
	Holly Carter
**Response to Reviewers:**	These responses are available in the uploaded file 'Response to Reviewers'
**Additional Information:**	
**Question**	**Response**
**Financial Disclosure**<br><br>Enter a financial disclosure statement that describes the sources of funding for the work included in this submission. Review	The authors received no specific funding for this work.<br><br>HC and CS are supported by the National Institute for Health Research Health Protection Research Units (NIHR HPRU) in Emergency Preparedness and Response, a partnership between UK Health Security Agency (UKHSA), King's College London

Powered by Editorial Manager® and ProduXion Manager® from Aries Systems Corporation

the submission guidelines for detailed requirements. View published research articles from *PLOS ONE* for specific examples.<br><br>This statement is required for submission and **will appear in the published article** if the submission is accepted. Please make sure it is accurate.<br><br>**Unfunded studies**<br>Enter: *The author(s) received no specific funding for this work.*<br><br>**Funded studies**<br>Enter a statement with the following details:<br>• Initials of the authors who received each award<br>• Grant numbers awarded to each author<br>• The full name of each funder<br>• URL of each funder website<br>• Did the sponsors or funders play any role in the study design, data collection and analysis, decision to publish, or preparation of the manuscript?<br>• **NO** - Include this sentence at the end of your statement: *The funders had no role in study design, data collection and analysis, decision to publish, or preparation of the manuscript.*<br>• **YES** - Specify the role(s) played.<br><br>* typeset	and the University of East Anglia. The views expressed are those of the authors and not necessarily those of the NIHR, UKHSA or other government departments
**Competing Interests**<br><br>Use the instructions below to enter a competing interest statement for this submission. On behalf of all authors, disclose any competing interests that could be perceived to bias this work—acknowledging all financial support and any other relevant financial or non-financial competing interests.<br><br>This statement is required for submission and **will appear in the published article** if the submission is accepted. Please make sure it is accurate and that any funding sources listed in your Funding Information later in the submission form are also	The authors have declared that no competing interests exist.

Powered by Editorial Manager® and ProduXion Manager® from Aries Systems Corporation

declared in your Financial Disclosure statement.<br><br>View published research articles from *PLOS ONE* for specific examples.<br><br>**NO authors have competing interests**<br><br>Enter: *The authors have declared that no competing interests exist.*<br><br>**Authors with competing interests**<br><br>Enter competing interest details beginning with this statement:<br><br>*I have read the journal's policy and the authors of this manuscript have the following competing interests: [insert competing interests here]*<br><br>* typeset	
**Ethics Statement**<br><br>Enter an ethics statement for this submission. This statement is required if the study involved:<br><br>• Human participants<br>• Human specimens or tissue<br>• Vertebrate animals or cephalopods<br>• Vertebrate embryos or tissues<br>• Field research<br><br>Write "N/A" if the submission does not require an ethics statement.<br><br>General guidance is provided below. Consult the submission guidelines for detailed instructions. **Make sure that all information entered here is included in the Methods section of the manuscript.**	N/A

**Format for specific study types**<br><br>**Human Subject Research (involving human participants and/or tissue)**<br>• Give the name of the institutional review board or ethics committee that approved the study<br>• Include the approval number and/or a statement indicating approval of this research<br>• Indicate the form of consent obtained (written/oral) or the reason that consent was not obtained (e.g. the data were analyzed anonymously)<br><br>**Animal Research (involving vertebrate animals, embryos or tissues)**<br>• Provide the name of the Institutional Animal Care and Use Committee (IACUC) or other relevant ethics board that reviewed the study protocol, and indicate whether they approved this research or granted a formal waiver of ethical approval<br>• Include an approval number if one was obtained<br>• If the study involved *non-human primates*, add *additional details* about animal welfare and steps taken to ameliorate suffering<br>• If anesthesia, euthanasia, or any kind of animal sacrifice is part of the study, include briefly which substances and/or methods were applied<br><br>**Field Research**<br><br>Include the following details if this study involves the collection of plant, animal, or other materials from a natural setting:<br>• Field permit number<br>• Name of the institution or relevant body that granted permission	
**Data Availability**<br><br>Authors are required to make all data underlying the findings described fully available, without restriction, and from the time of publication. PLOS allows rare exceptions to address legal and ethical concerns. See the PLOS Data Policy and FAQ for detailed information.	Yes - all data are fully available without restriction

Powered by Editorial Manager® and ProduXion Manager® from Aries Systems Corporation

A Data Availability Statement describing where the data can be found is required at submission. Your answers to this question constitute the Data Availability Statement and **will be published in the article**, if accepted.<br><br>**Important:** Stating 'data available on request from the author' is not sufficient. If your data are only available upon request, select 'No' for the first question and explain your exceptional situation in the text box.<br><br>Do the authors confirm that all data underlying the findings described in their manuscript are fully available without restriction?	
**Describe where the data may be found in full sentences. If you are copying our sample text, replace any instances of XXX with the appropriate details.**<br><br>• If the data are **held or will be held in a public repository**, include URLs, accession numbers or DOIs. If this information will only be available after acceptance, indicate this by ticking the box below. For example: *All XXX files are available from the XXX database (accession number(s) XXX, XXX.*).<br>• If the data are all contained **within the manuscript and/or Supporting Information files**, enter the following: *All relevant data are within the manuscript and its Supporting Information files.*<br>• If neither of these applies but you are able to provide **details of access elsewhere**, with or without limitations, please do so. For example:<br><br>*Data cannot be shared publicly because of [XXX]. Data are available from the XXX Institutional Data Access / Ethics Committee (contact via XXX) for researchers who meet the criteria for access to confidential data.*<br><br>*The data underlying the results presented in the study are available from (include the name of the third party*	All data that were used for synthesis are available in the data extraction table. This table can be found in the file titled 'S3_Appendix' in the Supporting Information zip file. Please note that we did not have access to the original raw data for each individual study included in our review. The data that were synthesised in our analysis were extracted from the results sections of each paper.

and contact information or URL).<br>• This text is appropriate if the data are owned by a third party and authors do not have permission to share the data.<br><br>* typeset	
Additional data availability information:	

Powered by Editorial Manager® and ProduXion Manager® from Aries Systems Corporation

Manuscript Click here to access/download;Manuscript;Manuscript.docx

Effect of face-covering use on adherence to other COVID-19 protective behaviours: a systematic review

Adam Millest[1], Sidra Saeed[1], Charles Symons[1], Holly Carter[1*]

[1]Behavioural Science and Insights Unit, UK Health Security Agency, London, United Kingdom

*Corresponding author

Email: behaviouralscience@ukhsa.gov.uk (HC)

Abstract

During the COVID-19 pandemic, concerns were raised that face covering use may elicit risk compensation; a false sense of security resulting in reduced adherence to other protective behaviours such as physical distancing. This systematic review aimed to investigate the effect of face covering use on adherence to other COVID-19 related protective behaviours. Medline, Embase, PsychInfo, EmCare, medRxiv preprints, Research Square and WHO COVID-19 Research Database were searched. All primary research studies published from 1st January 2020 to 17th May 2022 which investigated the effect of face covering use on adherence to other protective behaviours in public settings during the COVID-19 pandemic were included. Papers were selected and screened in accordance with the PRISMA framework. Backwards and forwards citation searches of included papers were also conducted on 16th September 2022, with eligible papers published between 1st January 2020 and that date being included. A quality appraisal including risk of bias was assessed using the Academy of Nutrition and Dietetics' Quality Criteria Checklist. This review is registered on PROSPERO, number CRD42022331961. 47 papers were included, with quality ranging from low to high. These papers investigated the effects of face covering use and face covering policies on adherence to six categories of behaviour: physical distancing; mobility; face-touching; hand hygiene; close contacts; and generalised protective behaviour. Results reveal no consistent evidence for or against risk compensation, with findings varying according to behaviour and across study types. There is a suggestion that face covering use might reduce face-touching and face covering mandates might increase mobility, though the lack of robust evidence means these are tentative claims. Evidence on the other protective behaviours is largely inconsistent, and therefore confident conclusions cannot be made in these areas. Any policy decisions related to face coverings must consider the inconsistencies and caveats in this evidence base.

1. Introduction

The COVID-19 pandemic highlighted the important role that public behaviour can play in the control of infectious disease. Prior to the introduction of vaccination, measures to control the spread of COVID-19 consisted primarily of non-pharmaceutical interventions (NPIs) (e.g. hand cleaning; reducing contact with others; staying at home) and these NPIs included the use of face-coverings (1). Over 160 governments mandated or recommended face-coverings during the pandemic (2), and their use was seen as crucial to controlling the spread of COVID-19.

At the onset of the pandemic however, concerns were raised that the use of face coverings may provide a false sense of security, leading to lower adherence to other protective measures such as hand hygiene and physical distancing; a phenomenon known as 'risk compensation' (3) that has its theoretical origins in both economonics and psychology. Peltzman (4), for example, proposes that safety is a 'good' that we will 'trade in' for other goods in cases where the level of safety we 'have' exceeds our desired level. For example, in the case of driving cars, the theory predicts that safer cars will prompt riskier driving as drivers trade in the excess level of safety (that is, a level of safety that is above their required or desired level) to attain greater 'driving intensity' (5). In the case of face-coverings therefore, this theory might predict that face-coverings provide a level of safety that exceeds the desired level, and people will trade in that excess for goods such as increased social connection (by reducing physical distancing) or convenience (by spending less time washing hands). Wilde (6) predicts such behaviour through a phenomenon he refers to as 'risk homeostasis.' Wilde posits that, just as our bodies have a certain biological balance that we unconsciously look to maintain, psychologically we have an optimum level of risk that we also seek to maintain, and that, depending on the circumstances we find ourselves in, we will look to make risk-related adjustments. As such if we find ourselves in circumstances in which our level of risk is below this optimum level, we will look to increase it through our behaviour. In the case of face-covering use, this theory would predict that by wearing face-coverings our risk of catching or transmitting a virus decreases to below our optimum level, and so we look to achieve risk homeostasis, by, for example, reducing our physical distancing. In summary, though these two theories take a slightly different angle, both predict that when

our behaviour or circumstances reduce the level of risk we are subjected to, we look to compensate for that risk by undertaking behaviour that has an equal and opposite effect.

There is no clear consensus on the existence of risk compensation (7, 8). One review was carried out early in the pandemic to examine the extent to which risk compensation may affect public behaviour (9), however, given the paucity of research into COVID-19 specifically at the time, this review necessarily relied on research into risk compensation in other contexts. In a study in Germany, Henk et al. (10) found that self-reported adherence to protective behaviours was significantly lower after the implementation of two policies: mandatory quarantine for visitors to the country and mandatory face-covering use in shops and on public transport. Similarly, Jones Ritten et al. (11) investigated the risk compensating effect of COVID-19 testing by surveying students at two large universities in the US and found a positive association between testing behaviour and the number of risky events attended (e.g., large indoor gatherings). Moreover, it was found that those who participated in the testing programme perceived that doing so reduced their risk of contracting COVID-19, and further, that there was a positive association in the perceived risk-reducing effect of the testing programme and frequency of attending risky events; that is, the more respondents believed that testing reduced their risk of contracting COVID-19, the more likely they were to attend risky events. In contrast, Ludema et al., (12), used a randomised controlled trial to investigate risk compensation and anti-body testing and found that, irrespective of whether the result was positive or negative, receiving antibody test results did not lead to significant changes in adherence to COVID-19 protective behaviours. The extent to which risk compensation has occurred during the COVID-19 pandemic is therefore unclear.

There is now a substantial body of research investigating the risk compensatory effect of face covering use in the context of COVID-19. The aim of this systematic review was therefore to identify, summarise and assess the findings from studies examining the effect of face covering use on adherence to other protective behaviours in the context of COVID-19.

2. Methods

Details of the the protocol for this systematic review were registered on PROSPERO before screening took place. The protocol's number is CRD42022331961 and can be accessed at https://www.crd.york.ac.uk/PROSPERO/display_record.php?RecordID=331961 (13). It can also be seen in S6 Appendix.

2.1 Eligibility criteria

Studies were eligible if they reported primary quantitative or qualitative research relating to the impact of face covering use on adherence to other protective behaviours during the COVID-19 pandemic. Protective behaviours of interest included but were not limited to: COVID-19 physical/social distancing, hygiene (e.g., avoidance of face-touching, regular handwashing), staying at home, and reducing travel. Studies which examined the effectiveness and/or efficacy of wearing face coverings or factors related to adherence to face covering use were excluded. Published research and pre-publication articles were included. Reviews, position/discussion papers, conference abstracts, protocol papers, modelling studies, case reports and studies published in languages other than English were excluded (see Table 1).

Table 1. Inclusion criteria.

**Inclusion Criteria**	**Exclusion Criteria**
• Papers published from 1st January 2020 to present day at time of search (17th May 2022)<br>• All populations and all public settings other than healthcare settings<br>• Quantitative or qualitative research relating to the impact of face covering use on adherence to other protective behaviours during the COVID-19 pandemic	• Healthcare settings<br>• Papers not in English<br>• Systematic reviews, narrative reviews, guidelines, position/discussion papers, conference abstracts, protocol papers, modelling studies and case reports.

• Face covering use: i. All types of face covering, including (but not limited to) handmade and commercial cloth coverings (cloth, cotton, gauze, etc), and medical coverings such as surgical face masks. ii. Face covering mandate.<br>•Experimental studies, observational studies, qualitative studies, laboratory studies, any other primary data, secondary data analyses.<br>• Published research and pre-publication articles	• Studies which examined the effectiveness and/or efficacy of wearing face coverings or factors related to adherence to face covering use

2.2 Search strategy

A systematic search was conducted by UKHSA researchers on 17th May 2022 for papers from January 2020 until the date of searches. Sources searched included Ovid Medline, Ovid Embase, Ovid PsychInfo, Ovid EmCare, medRxiv preprints, Research Square and WHO COVID-19 Research Database. Search terms included terms related to COVID-19 (e.g. COVID-19, coronavirus, Sars-COV2), face coverings (e.g. mask, face cover, mouth covers) and protective behaviours (e.g., social distancing, handwashing, face-touching). A complete list of search terms is available in S1 Appendix. Forward and citation searches of included papers were also conducted on September 16th 2022, and eligible papers from January 1st2020 to that date were also included.

2.3 Study identification

Selection and screening of papers followed a systematic search method following a Preferred Reporting Items for Systematic Reviews and Meta-Analyses (PRISMA) framework (14). The study identification process is detailed in Fig 1 and contains full details of papers included and excluded at each stage. Papers were initially title- and abstract-screened (decisions were made as to the eligibility of each paper following a review of their title and abstract alone). In the first stage of title and abstract screening, 10% of records were assessed in duplicate by two reviewers, and disagreements were resolved by discussion and

consensus. In the second stage of title and abstract screening one reviewer assessed the remaining 90% of records. The screening tool Rayyan (15) was used for both these first and second stages. All relevant records were then screened by full text by one reviewer (decisions were made as to the eligibility of each paper following a review of the paper in full). These decisions were then checked by a second reviewer. There were five reasons for exclusion: (i) The direction of analysis was incorrect e.g., the study investigated behavioural predictors of face-covering use rather than vice versa (n = 5), (ii) the indepdent variables or outcome variables were not relevant to the research question (n = 14), (iii) the study did not comprise primary empirical research (n = 6), (iv) the paper was a previously undetected duplicate (n = 5), and (v) the paper was not written in English (n = 1). By the end of this process, 47 papers were deemed eligible and included in the review.

Fig 1. PRISMA 2020 flow diagram of the identification of studies process

2.4 Data extraction and synthesis

Data was sought for any relevant COVID-19 protective behaviour outcomes; at the completition of extraction, these comprised the following behavioural outcomes: physicial distancing, mobility, face-touching, hand hygiene, close contacts and generalised measures of protective behaviour. All results that were compatible with each of these outcomes were included. The following information was then collated for each study: country, publication status, population and sample size, methods, key outcome variables and main results. This was initially collated into one table which included an outcome variable column. This table was then divided by outcome variable into six smaller data extraction tables, and a narrative summary of results was produced, structured by these six outcome variable categories.

Data extraction was completed for each included study by one reviewer and independently checked by a second reviewer, with discrepancies resolved by discussion. Any evidence that was not directly relevant to the review question was not extracted.

2.5 Quality assessment

Quality including risk of bias was evaluated by one reviewer using the Quality Criteria Checklist (QCC) tool which can be used to assess the methodological quality of a study (16). Using the QCC, studies were characterised as high, medium or low quality.

This checklist tool is composed of 10 questions, four of which are considered critical (questions on selection bias, group comparability, description of exposure/assessment of transmission routes, and validity of outcome measurements); full details can be seen in S2 Appendix. A study was rated as high methodological quality if the answers were yes to the four critical questions plus at least one of the remaining questions. A study was rated as low methodological quality if answers were no to >50% of the 10 questions. Otherwise, the study was rated as medium methodological quality. In line with a systematic review which found that self-report methods of capturing adherence to protective behaviours can over-estimate objectively observed adherence by up to a factor of five (17), any studies which employed a self-report methodology were deemed not to satisfy the validty of outcome criterion, and so were necessarily limited to a quality score of medium or below. An additional reviewer independently assessed five of the studies, and conflicting findings were discussed. Following discussions, a potential source of bias was identified in the first reviewer's application of one of the tool's questions, and all studies were subsequently re-evaluated to correct for this bias.

3. Results

3.1 Overview of search results

The initial searches generated 12,662 articles. After de-duplication, 7,980 records remained, and title and abstract screening resulted in 70 papers being accepted for full text screening. Following full text screening, 39 papers were accepted for inclusion in the review. Backwards and forwards citation searches were conducted which yielded a further 8 papers for inclusion, meaning the total number of papers included in the review was 47. Quality appraisal of the articles revealed that nine were of a high quality, 33 of a

medium quality, and five of a low quality. Of the papers included nine were pre-prints, 35 were peer-reviewed publications, and three were published conference papers. Finally, it was found that some papers included more than one methodology, intervention or protective behaviour and thus, the number of studies described for each of these categories may exceed the total number of papers.

All studies included used quantitative methods, and none used qualitative methods. These included field experiments (n = 7), natural experiments (n = 14), lab experiments (n = 14), observational studies (n = 10) and cross-sectional studies (n = 9). Of these, 30 studies measured behaviour through observation, and 22 measured behaviour through self-report. Of the self-report studies, 10 were concerned with current or previous behaviour, and 12 with expected or intended behaviour. Though all included papers necessarily investigated the effect of face covering use on protective behaviours, the exact intervention of focus in each case was varied and fit into one of five categories: face covering use (self) i.e., the act of wearing a face covering oneself (n = 21); face covering use (other) i.e., in some way interacting with someone else wearing a face covering (n = 18); face covering use (self and other) i.e., in some way interacting with someone else who is wearing a face covering whilst also wearing a face covering oneself (n = 1); face covering policy i.e., the implementation of a face covering mandate (n = 19); and face covering intervention package i.e., a community programme designed to increase uptake of face covering usage (n = 1). The protective behaviour(s) in each case also varied, and in each case fit into one of 6 categories: physical distancing i.e., interpersonal distance (n = 30); mobility i.e., the extent to which people leave their home and visit public or residential spaces (n = 13); face-touching (n = 7); hand hygiene e.g., hand washing, avoidance of handshakes (n = 5); close contacts i.e., the extent to which people have close and extended interactions with people outside of their household (n = 4); and generalised protective behaviour i.e., a single measure to encapsulate two or more COVID-19 protective behaviours other than face-covering use, e.g, hand-washing, physical distancing and staying at home (n = 2). Full details on each paper can be seen in the data extraction tables in S3 Appendix.

Some studies initially appeared to meet the inclusion criteria but were ultimately excluded. For example, one study investigated how face covering use affected perceptions of physical distance between oneself and another person, however, since it did not investigate how face covering use affected distancing

behaviour, it was not deemed to be eligible for inclusion (18). Similarly, another study investigated participants' judgements on how face covering use moderates the relationship between a given physical distance and transmission risk was not included since, although its focus was related to the review's objectives, it did not directly address it, in that it did not investigate how face covering use directly impacted adherence to protective behaviours (19) .

The section below presents findings in relation to each of these six behaviours and describes the extracted data in terms of the effect that face covering use has on each type of behaviour, also exploring any patterns with regards to how the effect of face covering use varies by the type of intervention, and the type of study design.

3.2 Physical distancing (see table 2a in S3 Appendix for full details)

Outcomes were mixed and varied depending on the type of study. Findings from the majority of lab experiments (10 of 13) indicated that physical distancing was lower in conditions where a face covering was used by another person compared with conditions where it was not (20-27), (28: two studies), and two of these studies also found that physical distancing was lower when a face covering was used by the self (28: two studies). Additionally, one of these studies investigated how physical distancing in conditions with and without face covering use compared with a baseline measure (that is, physical distancing outside the context of face covering use) (20). It was found that physical distancing was significantly greater than the baseline in conditions without face covering use, but not significantly different in conditions with face covering use, potentially suggesting that differences between conditions is driven by participants moving away from people without face coverings (relative to where they would normally position themselves), rather than towards people with face coverings. Of the remaining three studies, one found that physical distancing was lower in conditions of face covering use (other), but that this effect was conditional on situational factors such as effort required to maintain physical distance, and age of the other person (29), and two found that distancing was greater in the case of face covering use (other) (30), (31: Study 2).

In contrast, the majority of field experiments (5 of 6) found that physical distancing was either greater or not significantly different in conditions where face coverings were used, compared to conditions where they were not. Two studies found that physical distancing was greater in conditions where face coverings (self or other) were used compared to when they were not (32), (31: Study 1), with a third study finding that physical distancing was greater in areas where a face covering intervention package had been implemented compared to those where it had not (33). One study found mixed effect[illegible]inding that face covering use (self) had no effect on distancing, but that distancing was greater in conditions of face covering use (other) (34), while another study found no differences in distancing between conditions of face covering use (self and other) and conditions without face covering use (35). A minority of field experiments (1 of 6) found that physical distancing was lower in conditions of face covering use (other), but that this effect was conditional on gender of participant, race and social status of confe[illegible]ate, and face covering policy implemented at the time (36). Additionally, three of the above studies also investigated through a natural design whether the effect of face covering use on physical distancing differed between different time periods wh[illegible] varied by the face covering policy (mandatory or voluntary) implemented at the time. One study found that face-covering use by others significantly reduced physical distancing adherence amongst men only, both in mandatory and in voluntary face covering policy contexts; the effect was slightly more pronounced in the mandatory context (36). The two other studies found no difference between policy contexts; both found that face covering use (other) increased physical distancing both when it was mandated and when it was voluntary (32, 34).

The four natural experiments explored the impact of face covering policy on physical distancing. Three of these found that physical distancing did not significantly differ according to whether face covering wearing was voluntary or mandatory (37: Study 2), (28: Study 2), (34), while a third found that the impact of face covering policy on physical distancing was dependent on context; attention given to physical distancing decreased when a face covering mandate was introduced on public transport, but increased when the mandate was expanded to bars and restaurants, and again when expanded to all public spaces (38).

The four observational studies found either a positive or a neutral relationship between face covering use or face covering policy and physical distancing. Three observational studies found no significant

relationship between face covering use (self) or face covering policy and physical distancing (37: two studies), (39), while the fourth observational study found that physical distancing was greater in those who wore face coverings, and also that it was greater when a face covering mandate was implemented (40).

Four of the six correlational studies found that as self-reported face covering use (self or other) increased, so did self-reported physical distancing (41-44). One study showed mixed results for the relationship between face covering use and physical distancing, finding that those who reported wearing face coverings all the time were more likely to report adherence to physical distancing compared with those who reported never doing so, but those who reported wearing face coverings sometimes were more likely to report having reduced distancing with people than those who reported never doing so (45). The final correlational study found no relationship between face covering use (self) and physical distancing (46).

3.3 Mobility (see table 2b in S3 Appendix for full details)

Of the 10 natural experiments, five found that when face covering policies were in place mobility increased (i.e., people were less likely to stay at home) (47-51), four found that mobility was unchanged, (52-55) and one found that mobility decreased (i.e., people were more likely to stay at home) (56). The lab experiment found that mobility is likely to increase when policies are in place, as participants reported being more willing to use the London Underground when a face covering mandate was in operation (57).

Of the two cross-sectional studies, one found that self-reported adherence to personal protective behaviours, which included face covering use (self) predicted self-reported frequency of going out in public, and that this relationship was mediated by belief in ‘substitution myths’; the belief that one protective behaviour can be substituted for another, with risk staying constant (46). The second cross-sectional study found that those who reported always wearing a face covering outside their home were less likely to report visiting a friend, neighbour, bar or club, compared with those who did not report always wearing a face covering outside their home (44).

3.4 Face-touching (see table 2c in S3 Appendix for full details)

The field experiment found that face covering use (self) resulted in less face-touching; there was a significant increase in the number of individuals who touched their eyes or hair when they were not wearing a face covering, and, when investigating areas not covered by a face covering (hair, forehead, eyes and ears) the absolute number of total touches was significantly higher in those not wearing one (58). Two of the six observational studies found similar results; those who wore face coverings were less likely to touch their face (59, 60). Two more observational studies found that results were dependent on the operationalisation of face-touching; when face-touching was defined as making contact with any part of the face (including touches to the mask), there was no significant relationship between face covering use (self) and face-touching, but when it was defined as making contact with specific parts of the face (not including touches to the mask), those who wore face coverings touched their faces less frequently (61: two studies). A minority of observational studies (1 of 6) found a greater frequency of face-touching in those who wore coverings compared with those who did not (62). The final observational study found that face covering policy had no impact on face-touching; there was no statistically significant association between the face covering policy in operation and frequency of face-touching (63).

3.5 Hand hygiene (see table 2d in S3 Appendix for full details)

The natural experiment found that face covering policy had no impact on hand hygiene; attention paid to hygiene practices was not significantly different according to the implementation of different face covering policies (38). The three cross-sectional studies found that those who reported wearing face coverings more often were more likely to report also adhering to hand hygiene practices (41, 44, 45).

3.6 Close contacts (see table 2e in S3 Appendix for full details)

One of the two natural experiments found that the number of close contacts was significantly lower when a face covering mandate was expanded from public transport to include restaurants and bars, and lower again when expanded to all public spaces (38). The other natural experiment found that a face covering mandate did not affect whether participants cancelled or postponed personal or social activities (55).

One of the three cross-sectional studies found that the relationship between face covering use (self) and number of close contacts was mixed; those who reported wearing face coverings all the time were more likely to report spending less than 15 minutes in close contact with someone compared with those who reported never wearing face coverings, while those who reported sometimes wearing face coverings were more likely to report spending over 60 minutes in close contact with someone compared with those who reported never doing so (45). The second cross-sectional study found that those who did not always wear face coverings outside work were more likely to have close contact with people they do not live with (44), whilst the final cross-sectional study found that self-reported face covering use outside of work significantly predicted a greater number of self-reported daily contacts (64).

3.7 Generalised protective behaviour (see table 2f in S3 Appendix for full details)

The term 'generalised protective behaviour' was used to categorise any studies whose outcome variable was a single measure which encapsulated two or more other COVID-19 protective behaviours besides face covering use e.g., hand washing, physical distancing and staying at home. Both studies found that self-reported face covering use (self) was associated with self-reported behaviours that are likely to reduce the spread of COVID-19. In one study the majority of participants reported that their adherence to other protective behaviours was either unchanged or greater when they wore a face covering compared with when they did not (65), while the other study found that self-reported face covering use (self) was associated with self-reported reduced engagement with COVID-19 related risky behaviour (66).

3.8 Quality assessment

Few clear patterns were found when analysing the results of studies cross-sectionally by quality. With the exception of face-touching, for all behaviours (physical distancing, mobility, hand hygiene, close contacts and generalised protective behaviour) study quality was mixed for those that found positive, negative, neutral or equivocal effects of (or associations with) face covering use. For face-touching, studies were either of a low or medium quality, with all four studies of medium quality finding the same result: a negative

effect of (or association with) face covering use. The three studies of low quality were mixed: one found a positive association, one found a negative association and one found a nonsignificant association.

4. Discussion

Although face coverings have been found to reduce the transmission of airborne diseases, concerns have been raised as to the effect that their use may have on adherence to other COVID-19 protective behaviours. While several studies which explore this issue have been conducted, they had not previously been brought together in a review. This systematic review has identified and summarised the findings of 47 papers which collectively investigate the effect of face covering use on other COVID-19-related protective behaviours, namely, physical distancing, mobility, face-touching, hand hygiene, close contacts and generalised protective behaviour. Findings showed that results varied considerably, and that the nature of the variation often depended upon the specific behaviour investigated and the type of study employed to investigate it. The sections below, organised by behaviour, summarise and discuss the findings.

4.1 Physical distancing

One of the clearest patterns emerging from the studies relating to physical distancing was the distinction between the respective findings of lab experiments and field experiments. The majority of lab experiments found that physical distancing was lower in conditions of face covering use (compared with conditions without face covering use), whereas the majority of field experiments found that physical distancing was either greater or unchanged in conditions of face covering use.

A number of considerations should be made when interpreting these results, starting with the respective merits of field and lab experiments. There are three main advantages of lab experiments. First, the majority of the lab experiments employed a within-subjects design, meaning that individual differences which may impact ~~on~~ physical distancing (e.g., age, gender, risk perception, conscientiousness) are essentially controlled for in a way that cannot be done with between-subjects field experiments (67) (although estimates of such variables, e.g. age, were in some cases recorded and controlled for in the field

experiments). Second, the lab experiments typically allowed for more precision in data collection. The majority of lab experiments used an on-screen slider for participants to indicate their preferred distances, or a virtual reality environment in which computers captured the distances participants maintained from virtual agents. In contrast, some of the field experiments employed human judgement to determine whether a physical distancing guideline had been violated, increasing the risk of bias. However, four of the six field experiments used some form of electronic sensor to collect data (all of which found that physical distancing was greater or unchanged in conditions of face covering use). Third, lab experiments are able to examine the impact of face covering use on physical distancing in a context which is agnostic of face covering policy (mandatory vs voluntary), in contrast to field experiments which are necessarily conducted under periods of either mandatory or voluntary face covering wear (the potential for an interaction between face covering use and face covering policy is discussed in more detail in subsection 4.7).

However, whilst field experiments are less controlled than lab experiments, they are more ecologically valid (68). A key advantage of field experiments compared to lab experiments is that the disease transmission risk is real, not hypothetical; therefore a person's approach to physical distancing has a real impact on their own risk and on others' risk of contracting and spreading COVID-19. In all but one of the six field experiments not only were participants blinded to the experimental condition they were assigned to, but they were also unaware that they were taking part in an experiment at all, and so responses were unlikely to biased by, for example, demand characteristics. Behaviour captured in field experiments may therefore be more representative of and generalisable to wider populations than that collected during lab experiments, in which participants could second-guess research objectives, potentially causing demand effects (67). Additionally, in the majority of lab experiments, rather than being measured for actual behaviour, participants were asked to indicate distances they would prefer from the avatars or characters, allowing them to make a conscious decision which may or may not be indicative of how they might behave in real-life scenarios.

In addition to the lab and field experiments, the effect of face covering use on physical distancing was also investigated through a number of different methodologies; the majority of natural and observational studies tended not to find any significant associations between face covering use (or face covering policy) and

physical distancing, and cross-sectional studies tended to find positive associations between self-reported face covering-wearing and physical distancing. These different study types also have their respective strengths and weaknesses which should be considered when interpreting the results. The observational studies are necessarily high in ecological validity, however are less controlled (69). As such, the means of measurement (human judgement) may be lacking in precision and subject to bias, and furthermore, only claims of association (and not causality) can be made (70). For example it is likely that relationships between face covering-wearing and physical distancing are driven by confounding variables (e.g., risk perception, age, conscientiousness) impacting ~~on~~ both behaviours. The natural experiments, though again high in validity, are types of observational studies and so are subject to the same qualifications. Cross-sectional studies are also unable to draw claims of causality due to the likely impact of confounding variables (70), and may also be limited in their precision since measures are self-reported and not necessarily indicative of real-life behaviour (17).

A final consideration that should be made when interpreting the results is to consider the nature of the intervention in each case. Specifically, consideration should be given to: whether the independent variable (IV) is face covering condition of the self, face covering condition of another person, face covering policy, or some combination of any of the three; and the way in which physical distancing is operationalised in each case. Whilst there is no clear pattern with regards to how these different interventions and operationalisations of distancing impact outcomes, it is important to consider such differences when interpreting findings. Overall, it is clear that there is no consensus in relation to the impact of face covering use on physical distancing and that further research is required to bring the merits of different study types together and capture more reliable, and perhaps more consistent findings with regards to the effect of face covering use on physical distancing.

4.2 Mobility

The findings on mobility are also inconclusive, although there is more evidence to suggest that face covering policies increase mobility than there is to suggest that they decrease mobility. Natural experiments tended to compare changes in mobility from a baseline between areas and periods of time that varied according to the face covering policy implemented. The findings arising from such studies are

therefore likely to be confounded by any number of other variables which are likely to impact upon mobility (e.g., other COVID-19 public health measures, rate of infection at time/area of data collection) and claims of causality must be made with caution. Generally speaking, however, these studies employ rich data sources and complex statistical models to account for these variables as far as is possible, and so conclusions made from these studies can be arrived at with a certain level of confidence. If it is the case that implementing face covering policies increases mobility, a plausible explanation may be that a mandate instils confidence in people that if they are to leave home others are more likely to wear a face covering, leading to a greater sense of security. Further qualitative work could be conducted to investigate how face covering policies impact upon people's decision to leave home and move around their communities.

Two cross-sectional self-report studies which investigated how face covering use by the self impacted ~~on~~ mobility were inconclusive, with relationships found in both directions (44, 46). Interestingly however, in one case it was found that a positive relationship between self-reported adherence to personal protective behaviour (including wearing a face covering) and going out in public with people outside ones' household was mediated by a belief that one protective behaviour can be safely substituted for another (46), providing evidence in favour of the risk compensation hypothesis.

4.3 Face-touching

The impact of face covering use on face-touching was the most consistent of all the behavioural outcomes, with a general trend for face-touching to be lower in conditions of face covering use than in conditions without. This was found in a field experiment (58) and four of six observational studies investigating the relationship between face covering use by the self and face-touching (59, 60), (61: two studies). Whilst observational studies are subject to the same caveats described in the physical distancing subsection, the general pattern of a negative relationship between face touching and face covering use suggests that even if risk compensation is at play, it is not strong enough to result in a positive relationship. Furthermore, that this pattern is corroborated by the experimental study boosts the claim that face covering use might reduce face touching behaviour. Additionally, all of the relatively higher quality studies investigating face-touching (which were all of a medium, as opposed to a low quality) found face covering use to have a negative effect on (or a negative association with) face-touching. It should however be noted that different

operationalisations of face-touching were employed across face-touching studies, and that in cases where significant effects or associations were found, they did not always apply to all parts of the face. Further research using experimental designs is required in this area, but on the basis of the studies reviewed to date, it seems reasonable to conclude that face covering use reduces instances of face-touching.

4.4 Hand hygiene

Across four studies (one natural experiment and three cross-sectional studies), three studies found a positive relationship between either face covering use or face covering policy and hand hygiene practices and a fourth found no significant relationship. The three studies which found positive relationships were cross-sectional (41, 44, 46) and based on self-report surveys; findings therefore show that those who report wearing face coverings are more likely to also report practicing good hand hygiene, but the cross-sectional nature of the studies means a causal relationship cannot be established. Furthermore, these studies can only inform us on how the isolated practices of wearing a face covering and practising hand hygiene relate to one another and cannot tell us whether hand hygiene is affected whilst wearing a face covering. The natural experiment (38), which employed face covering policies as an IV and found no significant effect on self-reported attention paid to hygiene practices may be more informative, suggesting that implementing a face covering mandate has no ‘collateral’ effect on hand hygiene practices. It should be noted however that this study is also subject to the caveats around accuracy associated with self-report data. In summary, the literature on the effect of face covering use on hand hygiene is limited and relies on self-report data, possibly because hand hygiene is a relatively private practice that is difficult to investigate using an experimental or observational design. On the basis of the literature reviewed however, it can be said that there is no evidence thus far to suggest that face covering use causes a reduction in hand hygiene practices.

4.5 Close contacts

There was little consensus in the literature on close contacts. Across two natural experiments, it was found that face covering mandates either had no effect on close contacts, or that they reduced them (38, 55). Across three cross-sectional studies the results were mixed with both positive and negative relationships

found (44, 45, 64). It is therefore difficult to draw robust conclusions about the impact of face covering use on close contacts.

4.6 Generalised protective behaviour

Two cross-sectional studies (65, 66) investigated the relationship between face covering use by the self and generalised adherence to other protective behaviours and found a relationship between face covering use and increased adherence to other protective behaviours (or reduced undertaking of risky behaviours). As previously discussed, cross-sectional studies do not allow for claims of causality to be made, and confounding variables are likely to influence any relationships.

4.7 Risk compensation outcomes under different policies

An area for consideration is whether ~~the~~ risk compensation outcome varies according to whether face covering use is voluntary or mandatory. For example, it might be expected that when face covering use is voluntary, there is a stronger relationship between face covering use (i.e. self-selected face covering use) and adherence to other protective behaviours given that the decision to wear a face covering is more likely to be driven by one’s own risk perception rather than the extrinsic factor of the mandatory face covering policy. Equally, when face covering use is voluntary, their use may serve as a stronger social signal as to the preferences or condition of the person using them since others know that they are doing so despite the lack of a mandate. For example, voluntary use may signal that the user is particularly risk-averse and therefore others may be more likely to keep their distance out of consideration and respect. Alternatively, voluntary use may signal that the user is more likely to be infectious, and so others may be more likely to keep their distance for self-protective reasons.

In the present review, only three included studies compared the effect of face covering use across different face covering policies (mandatory or voluntary) and only through a natural design and for one protective behaviour (physical distancing). In all three studies, the effect of face covering use by others on physical distancing adherence was not influenced by policy context, although in one study the effect was reportedly slightly more pronounced in the mandatory than in the voluntary context. There were also 24 studies in which, although the authors did not assess impact of face covering policy, they did provide information on

the real-world policy implemented during the time of study; that is whether face covering use was mandatory, voluntary or whether the policy was varied (i.e., that for some of the duration of the study it was mandatory, and for some of the time it was voluntary). Thirteen focused on physical distancing (five carried out in a mandatory and six in a voluntary face covering context) and results across studies were mixed in both contexts. Two focused on mobility and in both cases face covering use was voluntary but a positive association was found in one and a negative association in the other. There were three studies focused on face-touching and three on hand hygiene. For both protective behaviours face covering policy varied across studies but the face-touching studies all reported negative effects or associations and the hand hygiene studies all reported positive effects or associations. Two studies were focused on close contacts, one of which was conducted under mandated face covering use and found the association to be equivocal, and one of which was conducted when face covering use was voluntary and found the association to be negative. A final study that focused on generalised protective behaviour was conducted under varied policies and found a positive association.

It is difficult to draw robust conclusions about any interaction between face covering use and face covering policy from studies included in this review. A particular limitation of the literature is that no study to date has applied type of face covering policy (mandatory or voluntary) as an independent variable to assess its interaction with face covering use on risk compensation for other protective behaviours. This is an evidence gap that should be addressed in future research.

4.8 Limitations

This review is novel in drawing together current literature on the impact of face covering use on other protective behaviours during the COVID-19 pandemic, and contributing to an understanding of risk compensation in this context. However, some limitations should be acknowledged when considering this review's findings. First, despite every attempt to apply the inclusion and exclusion criteria systematically and objectively, it is likely that some level of subjectivity may have impacted on paper screening and selection. To mitigate against this, 10% of the title and abstract screening was undertaken by two researchers, and any disagreements resolved by discussion and consensus. The full-text screening was also checked by a second researcher. Second, due to resource constraints (namely, the linguistic

limitations of the researchers), only papers published in English were included in the review, inevitably creating a geographical bias. For example, 32 of the 47 papers described studies carried out in the UK, Western Europe, Canada, North America or Australia.

4.9 Conclusion

Overall, findings relating to the impact of face covering use or policy on other protective behaviours are inconsistent, varying both according to the particular behaviour in question, and by study type. Evidence relating to the potential impact of risk compensation is also inconsistent; while some studies suggest risk compensation may play a role in shaping behaviour, others suggest it has no impact. Findings are particularly inconsistent in relation to physical distancing, with considerable discrepancy found between study types. Whilst assessments of behaviour in real-life settings tend to find that face covering use either increases or has no impact upon physical distancing, lab experiments tend to suggest that physical distancing decreases in conditions of face covering use. There is some suggestion that the impact of face covering use on physical distancing could be affected by whether or not mandatory face covering policies are in effect (which would indicate that conclusions drawn from a voluntary context should not be directly applied to a mandatory context and vice versa) but findings in this area are mixed and further assessment of this moderating effect is warranted. Of the other behaviours, findings (though mixed) tend to suggest that face covering mandates may increase mobility and that face covering use may decrease face touching. While some studies suggest a relationship between face covering use and increased hand hygiene practices and adherence to other protective behaviours, these studies are predominately cross-sectional and rely on self-report measures, and do not allow conclusions to be drawn about the impact of face covering use on adherence to these behaviours. Findings relating to the impact of face covering use on close contacts is mixed, and further research is needed.

Overall, this review highlights that there is no clear consensus in the literature as to whether face covering use improves adherence to other protective behaviours, reduces adherence to other protective behaviours, or has no impact. There are two key recommendations which can be made from this review: 1) further research, using more robust research designs, is needed to establish whether face covering use affects different types of protective behaviours; and 2) any policy decisions made in relation to

recommended or mandatory face covering use must take into account the inconsistencies and caveats in the evidence base, recognising that current evidence does not allow firm conclusions to be drawn about the potential impact of face covering use on adherence to other protective behaviours. Recommendations for future research are summarised below.

4.8.1 Recommendations for future research

- Further field experiments (or more ecologically valid lab experiments) investigating the effect of face covering use on physical distancing. Such studies might make a particular focus of investigating:
    - Differing effects of face covering use by the self and face covering use by another person
    - How different face covering policies might moderate the relationship between face covering use and physical distancing . For example, a field experiment could be conducted whereby two manipulations occur; one whereby the setting varies according to the face covering policy implemented (mandatory or voluntary), and one whereby a confederate appears either wearing or not wearing a face covering. Physical distancing from the confederate would then be measured. This would allow for the direct comparison between the moderating effects of voluntary and mandatory settings with more control over extraneous factors.
    - How effects might vary according to the operationalisation of distancing; for example, whether the absolute distance is measured, or whether a binary variable capturing whether or not a particular distance or guideline is adhered to is captured
- Lab experiments replicating those cited in this review, but which vary by the framing of physical distancing (i.e., self-protective or prosocial)
- Qualitative research investigating how differing face covering policies affect attitudes towards leaving the home and moving around the community
- Experimental studies investigating the effect of face covering use and face covering policies on face-touching, hand hygiene and close contacts

5. Acknowledgements

The authors thank Dr Joanna Milward for contributing to the development of the protocol and search terms, Nicola Pearce-Smith for creating the search strategies and running the searches themselves, as well as providing guidance on screening, and Megan Phillips for contributing to the quality appraisal as a second reviewer.

6. References

1. Ayouni I, Maatoug J, Dhouib W, Zammit N, Fredj SB, Ghammam R, et al. Effective public health measures to mitigate the spread of COVID-19: a systematic review. BMC Public Health. 2021;21:1-14.
2. The Royal Society and the British Academy. Face masks and coverings for the general public: behavioural knowledge, effectiveness of cloth coverings and public messaging. London, UK: The Royal Society; 2020.
3. World Health Organization. Advice on the use of masks in the context of covid-19: interim guidance. 2020. pp 1-5. https://apps.who.int/iris/bitstream/handle/10665/331693/WHO-2019-nCov-IPC_Masks-2020.3-eng.pdf?sequence=1&isAllowed=y
4. Peltzman S. The effects of automobile safety regulation. Journal of Political Economy. 1975 Aug 1;83(4):677-725.
5. Hedlund J. Risky business; safety regulations, risk compensation, and individual behaviour. Injury Prevention. 2000 Jun 1;6(2):82-9.
6. Wilde GJ. The theory of risk homeostasis: implications for safety and health. Risk Analysis. 1982 Dec;2(4):209-25.
7. Greenhalgh T. Face coverings for the public: laying straw men to rest. Journal of Evaluation in Clinical Practice. 2020;26(4):1070-7.
8. Martin G, Hanna E, Dingwall R. Urgency and uncertainty: covid-19, face masks, and evidence informed policy. BMJ. 2020;369:m2017.

9. Mantzari E, Rubin G, Marteau T. Is risk compensation threatening public health in the covid-19 pandemic? BMJ. 2020;370:m2913.

10. Henk K, Rosing F, Wolff F, Frenzel SB, van Dick R, Erkens VA, et al., An examination and extension of the Peltzman effect during the Covid-19 pandemic. Current Research in Ecological and Social Psychology. 2023;4:100091.

11. Jones Ritten C, Thunström L, Cherry T, Wulfhorst JD. Frequent mandatory COVID-19 testing may increase risk behaviour. PNAS Nexus. 2022 Nov;1(5):pgac247.

12. Ludema C, Rosenberg MS, Macy JT, Kianersi S, Luetke M, Chen C, et al. Does receiving a SARS-CoV-2 antibody test result change COVID-19 protective behaviors? Testing risk compensation in undergraduate students with a randomized controlled trial. PLoS ONE. 2022 Dec 20;17(12):e0279347.

13. Millest A, Carter H. Effect of face-covering use on adherence to other Covid-19 protective behaviours. PROSPERO 2022 CRD42022331961 2022 [Available from: https://www.crd.york.ac.uk/prospero/display_record.php?ID=CRD42022331961.

14. Page MJ, McKenzie JE, Bossuyt PM, Boutron I, Hoffmann TC, Mulrow CD, et al. The PRISMA 2020 statement: an updated guideline for reporting systematic reviews. BMJ. 2021;371:n71

15. Ouzzani M, Hammady H, Fedorowicz Z, Elmagarmid A. Rayyan—a web and mobile app for systematic reviews. Systematic Reviews. 2016;5(1):210.

16. Academy of Nutrition and Dietetics. Evidence analysis manual: steps in the academy evidence analysis process. 2016. [cited 2023 Mar 03]. Available from https://www.andeal.org/evidence-analysis-manual.

17. Davies R, Mowbray F, Martin A, Smith L, Rubin G. A systematic review of observational methods used to quantify personal protective behaviours among members of the public during the COVID-19 pandemic, and the concordance between observational and self-report measures in infectious disease health protection. BMC Public Health. 2022;22(1):1436.

18. Chakraborty S, Stefanucci J, Creem-Regehr S, Bodenheimer B. Distance estimation with social distancing: a mobile augmented reality study. In 2021 IEEE International Symposium on Mixed and Augmented Reality Adjunct (ISMAR-Adjunct); 2021 October 04-08; Bari, Italy. IEE; 2021. p. 87-91.

19. Svenson O. People underestimate the change of airborne corona virus exposure when changing distance to an infected person: on interpersonal distance, exposure time, face masks and perceived virus exposure. medRxiv [Preprint]. 2022 [cited 2023 Mar 03]. Available from http://medrxiv.org/content/early/2022/03/15/2022.03.14.22272341.abstract.

20. Biggio M, Bisio A, Bruno V, Garbarini F, Bove M. Wearing a mask shapes interpersonal space during COVID-19 pandemic. Brain Sciences. 2022;12(5):682.

21. Cartaud A, Quesque F, Coello Y. Wearing a face mask against Covid-19 results in a reduction of social distancing. PLoS ONE. 2020;15(12):e0243023.

22. Gulbetekin E. Effects of mask use and race on face perception, emotion recognition, and social distancing during the COVID-19 pandemic. Research Square [Preprint]. 2021 [cited 2023 Mar 03]. Available from https://assets.researchsquare.com/files/rs-692591/v1/1cab9894-2761-4622-b8b9-6d829c3d4343.pdf?c=1631886459.

23. Iachini T, Frassinetti F, Ruotolo F, Sbordone F, Ferrara A, Arioli M, et al. Psychological and situational effects on social distancing and well-being during the COVID-19 pandemic: not a question of real risk. PsyArXiv [Preprint]. 2020 [cited 2023 Mar 03]. Available from https://psyarxiv.com/r7usv/.

24. Lee Y, Chen Y. Influence of wearing surgical mask on interpersonal space perception between mainland Chinese and Taiwanese people. Frontiers in Psychology. 2021;12:692404.

25. Lisi MP, Scattolin M, Fusaro M, Aglioti SM. A Bayesian approach to reveal the key role of mask wearing in modulating projected interpersonal distance during the first COVID-19 outbreak. PLoS ONE. 2021;16(8):e0255598.

26. Xiong W, Wang, C., & Yu, X. The effect of face mask and approach pattern on interpersonal distance in COVID-19 pandemic using VR technology. In: Long S, Dhillon BS, editors. International Conference on Man-Machine-Environment System Engineering; 2022 Oct 21-23; Beijing, China. Singapore: Springer; 2022. p. 92-8.

27. Wang M, Lee Y-C. Investigating the effects of face Mask and gender on interpersonal distance judgments. In: Long S, Dhillon BS, editors. Man-Machine-Environment System Engineering: Proceedings of the 21st International Conference on MMESE; 2021 Oct 23-25; Beijing, China. Singapore: Springer; 2022. p. 40-5.

28. Luckman A, Zeitoun H, Isoni A, Loomes G, Vlaev I, Powdthavee N, et al. Risk compensation during COVID-19: The impact of face mask usage on social distancing. Journal of Experimental Psychology: Applied. 2021;27(4):722-38.

29. Kroczek LOH, Bohme S, Muhlberger A. Face masks reduce interpersonal distance in virtual reality. Scientific Reports. 2022;12(1):2213.

30. Bühler MA, Lynch SD, Bhojwani T, Zidan A, Fiset F, McFadyen BJ, et al. Stay out of the blast radius: influence of surgical masks on virtual pedestrian interactions. Research Square Research Square [Preprint]. 2021 [cited 2023 Mar 03]. Available from https://assets.researchsquare.com/files/rs-852079/v1/3e95f3ec-8eaf-46db-85df-e5283756a6ca.pdf?c=1638763153

31. Seres G, Balleyer AH, Cerutti N, Danilov A, Friedrichsen J, Liu YM, et al. Face masks increase compliance with physical distancing recommendations during the COVID-19 pandemic. Journal of the Economic Science Association. 2021 Nov 13;7:139-58.

32. Marchiori M. COVID-19 and the social sistancing paradox: dangers and solutions. arXiv [Preprint]. 2020 [cited 2023 Mar 03]. Available from https://arxiv.org/pdf/2005.12446.pdf?fbclid=IwAR3edT8vzdd_Rt8ykeVC8Y0RQ1hLs4A9Buny6d3hYc2TZqD5huEg5J478U4

33. Abaluck J, Kwong LH, Styczynski A, Haque A, Kabir MA, Bates-Jefferys E, et al.. Normalizing community mask-wearing: a cluster randomized trial in Bangladesh. National Bureau of Economic Research;2021 Apr. Report No.: 28734.

34. Seres G, Balleyer A, Cerutti N, Friedrichsen J, Suer M. Face mask use and physical distancing before and after mandatory masking: No evidence on risk compensation in public waiting lines. Journal of Economic Behavior & Organization. 2021;192:765-81.

35. Blanken TF, Tanis CC, Nauta FH, Dablander F, Zijlstra BJH, Bouten RRM, et al. Promoting physical distancing during COVID-19: a systematic approach to compare behavioral interventions. Scientific Reports. 2021;11:19463.

36. Aranguren M. Face mask use conditionally decreases compliance with physical distancing rules against COVID-19: gender differences in risk compensation pattern. Annals of Behavioral Medicine. 2022;56:332-46.

37. Liebst LS, Ejbye-Ernst P, de Bruin M, Thomas J, Lindegaard MR. No evidence that mask-wearing in public places elicits risk compensation behavior during the COVID-19 pandemic. Scientific Reports. 2022;12:1511.

38. Jørgensen F, Lindholt MF, Bor A, Petersen MB. Does face mask use elicit risk-compensation? Quasi-experimental evidence from Denmark during the SARS-CoV-2 pandemic. European Journal of Public Health. 2021;31(6):1259-65.

39. Puttock EJ, Marquez J, Young DR, Shirley AM, Han B, McKenzie TL, et al. Association of masking policies with mask adherence and distancing during the SARS-COV-2 Pandemic. American Journal of Infection Control. 2022;50:969-74.

40. Atzema CL, Mostarac I, Button D, Austin PC, Javidan AP, Wintraub L, et al. Assessing effective mask use by the public in two countries: an observational study. BMJ Open. 2021;11:e049389.

41. Betsch C, Korn L, Sprengholz P, Felgendreff L, Eitze S, Schmid P, et al. Social and behavioral consequences of mask policies during the COVID-19 pandemic. Proceedings of the National Academy of Sciences of the United States of America. 2020;117(36):21851-3.

42. Lama Y, Budenz A, Gaysynsky A, Iles IA, Chou WYS. Factors associated with COVID-19 behavioral intentions: findings from an online survey. American Journal of Health Promotion. 2022;36(7);1183-92.

43. Li M, Colby H. Association between actual and perceived U.S. COVID-19 policies and preventive behavior. Annals of Behavioral Medicine. 2021;55:369-75.

44. Sheehan MM, Pfoh E, Speaker SL, Rothberg M. Changes in social behavior over time during the COVID-19 pandemic. Cureus. 2020 Oct 01;12(10):e10754.

45. Doung Ngern P, Suphanchaimat R, Panjangampatthana A, Janekrongtham C, Ruampoom D, Daochaeng N, et al. Associations between wearing masks, washing hands, and social distancing practices, and risk of COVID-19 infection in public: a cohort-based case-control study in Thailand. medRxiv [Preprint]. 2020 [cited 2023 March 03]. Available from https://www.medrxiv.org/content/10.1101/2020.06.11.20128900v4

46. Griggs A, Weaver J, Alvarado J. Masking our risky behavior: how licensing and fear reduction reduce social distancing behavior. Journal of Social Psychology. 2022 Sep 3;162(5):607-20. Epub 2021 Jul 27.

47. Fracalossi de Moraes R, Russell LB, Santos da Silva LL, Toscano CM. Effects of non-pharmaceutical interventions on social distancing during the COVID-19 pandemic: evidence from the 27 Brazilian states. PLoS ONE. 2022;17(3):e0265346.

48. Hosseini MS, Gittler AM. Factors influencing human mobility during the COVID-19 pandemic in selected countries of Europe and North America. 2020 IEEE International Conference Big Data (Big Data); 2020 Dec 10-13; Atlanta, GA, USA. IEE; 2021. p. 4866-72.

49. Wadud Z, Rahman SM, Enam A. Face mask mandates and risk compensation: an analysis of mobility data during the COVID-19 pandemic in Bangladesh. BMJ Global Health. 2022;7:e006803.

50. Wang F, Ge, X, Huang D. Government intervention, human mobility, and COVID-19: a causal pathway analysis from 121 countries. Sustainability. 2022;14:3694.

51. Yan Y, Bayham J, Richter A, Fenichel EP. Risk compensation and face mask mandates during the COVID-19 pandemic. Scientific Reports. 2021;11:3174.

52. Barros V, Manes I, Akinwande V, Cintas C, Bar-Shira O, Ozery-Flato M, et al. A causal inference approach for estimating effects of non-pharmaceutical interventions during Covid-19 pandemic. medRxiv [Preprint]. 2022 [cited 2023 Mar 03]. Available from https://www.medrxiv.org/content/10.1101/2022.02.28.22271671v1

53. Chernozhukov V, Kasahara H, Schrimpf P. Causal impact of masks, policies, behavior on early covid-19 pandemic in the U.S. Journal of Econometrics. 2021;220:23-62.

54. Kovacs R, Dunaiski, M, Tukiainen J. Compulsory face mask policies do not affect community mobility in Germany. SSRN [Preprint]. 2020 [cited 2023 Mar 03]. Available from https://papers.ssrn.com/sol3/papers.cfm?abstract_id=3620070

55. Shin SH, Ji H, Lim H. Heterogeneity in preventive behaviors during COVID-19: health risk, economic insecurity, and slanted information. Social Science & Medicine. 2021;278:113944.

56. Zhao N, Yao S, Thomadsen R, Wang CB. The impact of social Distancing and masking on COVID-19 spread and consumer spending. SSRN [Preprint]. 2022 [cited 2023 Mar 03]. Available from https://papers.ssrn.com/sol3/papers.cfm?abstract_id=3725711

57. Bansal P, Kessels R, Krueger R, Graham DJ. Preferences for using the London Underground during the COVID-19 pandemic. Transportation Research Part A. 2022;160:45-60.

58. de Freitas AVC, Magalhães AM, Carricondo PC, Shinzato AA, Hida RY. Face masks can reduce the frequency of face touching: an observational crossover study. International Journal of Infectious Diseases. 2022;123:54-7.

59. Chen YJ, Qin G, Chen J, Xu JL, Feng DY, Wu XY, et al. Comparison of face-touching behaviors before and during the coronavirus disease 2019 pandemic. JAMA Network Open. 2020;3(7):e2016924.

60. Shiraly R, Shayan Z, McLaws ML. Face touching in the time of COVID-19 in Shiraz, Iran. American Journal of Infection Control. 2020;48:1559-61.

61. Liebst LS, Ejbye-Ernst P, de Bruin M, Thomas J, Lindegaard MR. Face-touching behaviour as a possible correlate of mask-wearing: a video observational study of public place incidents during the COVID-19 pandemic. Transboundary and Emerging Diseases. 2022;69:1319-25.

62. Kungurova Y, Mera R, Brewster E, Ali K, Fakoya AOJ. Covid-19 and face mask use: a St. Kitts case study. Open Access Macedonian Journal of Medical Sciences. 2020 Oct 26;8(T1):346-52.

63. Tao ZY, Dong J, Culleton R. The use of facemasks may not lead to an increase in hand-face contact. Transboundary and Emerging Diseases. 2020;67:3038-40.

64. van den Broek-Altenburg EM, Atherly AJ, Diehl SA, Gleason KM, Hart VC, MacLean CD, et al. Jobs, housing, and mask wearing: cross-sectional study of risk factors for COVID-19. JMIR Public Health and Surveillance. 2021;7(1):e24320.

65. MacIntyre CR, Nguyen PY, Chughtai AA, Trent M, Gerber B, Steinhofel K, et al. Mask use, risk-mitigation behaviours and pandemic fatigue during the COVID-19 pandemic in five cities in Australia, the UK and USA: a cross-sectional survey. International Journal of Infectious Diseases. 2021;106:199-207.

66. Guenther B, Galizzi MM, Sanders JG. Heterogeneity in risk-taking during the COVID-19 pandemic: evidence from the UK lockdown. Frontiers in Psychology. 2021 Apr 01;12:643653.

67. Charness G, Gneezy U, Kuhn MA. Experimental methods: between-subject and within-subject design. Journal of Economic Behaviour & Organization. 2012 Jan 01; 81(1):1-8.

68. Samek A. Advantages and disadvantages of field experiments. In: Schram A, Ule A. Handbook of research methods and applications in experimental economics. Cheltenham (UK) & Northampton (USA). Edward Elgar Publishing: 2019. p. 104-120.

69. Lu CY. Observational studies: a review of study designs, challenges and strategies to reduce confounding. The International Journal of Clinical Practice. 2009 May; 63(5):691-697.

70. Mann CJ. Observational research methods. Research design II: cohort, cross sectional, and case-control studies. Emergency Medicine Journal. 2003; 20:54-60.

7. Supporting information

S1 Appendix. Search strategies

S2 Appendix. Quality criteria checklist

S3 Appendix. Data extraction tables

S4 Appendix. PRISMA 2020 checklist

S5 Appendix. PRISMA 2020 abstract checklist

S6 Appendix. Protocol

Fig 1.

Click here to access/download;Figure;Fig 1.tiff

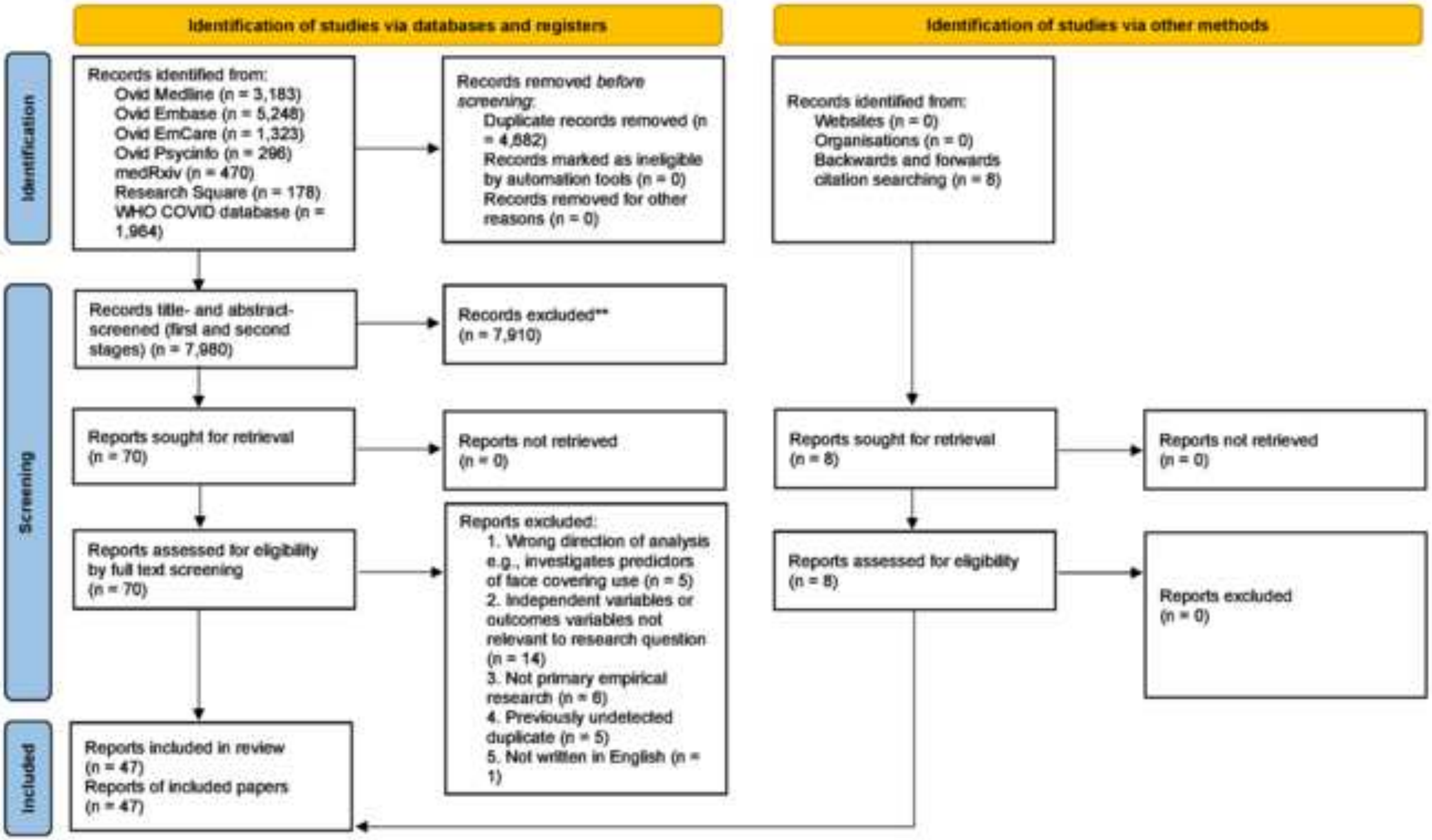

Click here to access/download
Supporting Information - Compressed/ZIP File Archive
Supporting Information.zip

Effect of face-covering use on adherence to other COVID-19 protective behaviours: a systematic review

Formatted: Font: Not Bold

Formatted: Centered

~~Authors:~~

Adam Millest[1*], Sidra Saeed[1], Charles Symons[1], Holly Carter[1*]

[1]Behavioural Science and Insights Unit, ~~(BSIU)~~ UK Health Security Agency, ~~UKHSA~~ London, United Kingdom

*Corresponding author

Email: behaviouralscience@ukhsa.gov.uk (HC)

Abstract

During the COVID-19 pandemic, concerns were raised that face covering use may elicit risk compensation; a false sense of security resulting in reduced adherence to other protective behaviours such as physical distancing. This systematic review aimed to investigate the effect of face covering use on adherence to other COVID-19 related protective behaviours. Medline, Embase, PsychInfo, EmCare, medRxiv preprints, Research Square and WHO COVID-19 Research Database were searched. All primary research studies published from 1st1 January 2020 to 17th May 2022 which investigated the effect of face covering use on adherence to other protective behaviours in public settings during the COVID-19 pandemic were included. Papers were selected and screened in accordance with the PRISMA framework. Backwards and forwards citation searches of included papers were also conducted on 16th September 2022, with eligible papers published between 1st January 2020 and that date being included. A quality appraisal including risk of bias was assessed using the Academy of Nutrition and Dietetics' Quality Criteria Checklist. This review is registered on PROSPERO, number CRD42022331961. 47 papers were included, with quality ranging from low to high. These papers investigated the effects of face covering use and face covering policies on adherence to six categories of behaviour: physical distancing; mobility; face-touching; hand hygiene; close contacts; and generalised protective behaviour. Results reveal no consistent evidence for or against risk compensation, with findings varying according to behaviour and across study types. There is a suggestion that face covering use might reduce face-touching and face covering mandates might increase mobility, though the lack of robust evidence means these are tentative claims. Evidence on the other protective behaviours is largely inconsistent, and therefore confident conclusions cannot be made in these areas. Any policy decisions related to face coverings must consider the inconsistencies and caveats in this evidence base.

Formatted: Superscript

1. Introduction

The COVID-19 pandemic highlighted the important role that public behaviour can play in the control of infectious disease. Prior to the introduction of vaccination, Prior to the introduction of vaccination, measures to control the spread of COVID-19 consisted primarily of non-pharmaceutical interventions (NPIs), (e.g. hand cleaning;, reducing contact with others;, and staying at home) and these NPIs included the use of face-coverings wearing a face covering to effectively reduce the spread and mortality rate of COVID-19 (1). The extent to which populations effectively undertook these behaviours was therefore crucial to controlling the spread of COVID-19. Over 160 governments mandated or recommended face-coverings during the pandemic (2), and their use was seen as crucial to controlling the spread of COVID-19.

A key behaviour which was used to control the spread of COVID-19 was the use of face coverings, with over 160 governments mandateding or recommendeding their use of face coverings during the COVID-19 pandemic (2). Many previous systematic reviews have been carried out on the transmission reduction efficacyof face coverings and other NPIs (e.g.,3, 4). This review aims to explore how face-covering use impacts on and interacts with other COVID-19 protective behaviours.

At the onset of the pandemic however, concerns were raised that the use of face coverings may provide a false sense of security, leading to lower adherence to other protective measures such as hand hygiene and physical distancing; a phenomenon known as 'risk compensation' (35) that has its theoretical origins in both economonics and psychology. Peltzman (4), for example, proposes that safety is a 'good' that we will 'trade in' for other goods in cases where the level of safety we 'have' exceeds our desired level. For example, in the case of driving cars, the theory predicts that safer cars will prompt riskier driving as drivers trade in the excess level of safety (that is, a level of safety that is above their required or desired level) to attain greater 'driving intensity' (5). In the case of face-coverings therefore, this theory might predict that face-coverings provide a level of safety that exceeds the desired level, and people will trade in that excess for goods such as increased social connection (by reducing physical distancing) or convenience (by spending less time washing hands). Wilde (6) predicts such behaviour through a phenomenon he refers to as 'risk homeostasis.' Wilde posits that, just as our bodies have a certain biological balance that we

unconsciously look to maintain, psychologically we have an optimum level of risk that we also seek to maintain, and that, depending on the circumstances we find ourselves in, we will look to make risk-related adjustments. As such if we find ourselves in circumstances in which our level of risk is below this optimum level, we will look to increase it through our behaviour. In the case of face-covering use, this theory would predict that by wearing face-coverings our risk of catching or transmitting a virus decreases to below our optimum level, and so we look to achieve risk homeostasis, by, for example, reducing our physical distancing. In summary, though these two theories take a slightly different angle, both predict that when our behaviour or circumstances reduce the level of risk we are subjected to, we look to compensate for that risk by undertaking behaviour that has an equal and opposite effect.

There is no clear consensus on the Arguments exist both for and against the existence of risk compensation (76, 87)., and o One review was carried out early in the pandemic to examine the extent to which risk compensation may affect public behaviour (98), .however, given the paucity of research into COVID-19 specifically at the time, this review necessarily relied on research into risk compensation in other contexts. In a study in Germany, Henk et al. (10) found that self-reported adherence to protective behaviours was significantly lower after the implementation of two policies: mandatory quarantine for visitors to the country and mandatory face-covering use in shops and on public transport. Similarly, Jones Ritten et al. (11) investigated the risk compensating effect of COVID-19 testing by surveying students at two large universities in the US and found a positive association between testing behaviour and the number of risky events attended (e.g., large indoor gatherings). Moreover, it was found that those who participated in the testing programme perceived that doing so reduced their risk of contracting COVID-19, and further, that there was a positive association in the perceived risk-reducing effect of the testing programme and frequency of attending risky events; that is, the more respondents believed that testing reduced their risk of contracting COVID-19, the more likely they were to attend risky events. In contrast, Ludema et al., (12), used a randomised controlled trial to investigate risk compensation and anti-body testing and found that, irrespective of whether the result was positive or negative, receiving antibody test results did not lead to significant changes in adherence to COVID-19 protective behaviours. The extent to which risk compensation has occurred during the COVID-19 pandemic is therefore unclear. However, given the

paucity of research into COVID-19 specifically, this review necessarily relied on research into risk compensation in other contextsMore recently, the concern about the potential impact of face coverings on other protective behaviours has prompted several studies examining this in the context of COVID-19, and there is now a substantial body of research that can contribute to discussions around risk compensation in this context.

Given the widespread recommendation and mandation of face coverings during the COVID-19 pandemic, it is essential to consider all available evidence in order to understand the potential impact of face covering use on adherence to other types of protective behaviours in the context of COVID-19.There is now a substantial body of research investigating the risk compensatory effect of face-covering use in the context of COVID-19. The aims of this systematic review wasere therefore to: identify, and summarise and assess the findings from studies examining the effect of face covering use on adherence to other protective behaviours in the context of COVID-19.; assess available evidence relating to the effect of face covering use on adherence to other COVID-19 protective behaviours. This aim was achieved and findings will help to inform decisions regarding if and when it is appropriate to recommend or mandate face covering use during future infectious disease outbreaks.

2. Methods

Details of the the protocol for this systematic review were registered on PROSPERO before screening took place. The protocol's number is CRD42022331961 and can be accessed at https://www.crd.york.ac.uk/PROSPERO/display_record.php?RecordID=331961 (1339). It can also be seen in S6 Appendix. As can be seen in the online record, the protocol was amended on 20th May 2022; these amendmends comprised edits to address inconsistencies with the regards to the decision to use the term 'face-covering' or 'face mask' throughout the protocol. Further amendments were made on 5th September 2022, 21st September 2022, 1st November 2022, and 3rd January 2023; these comprised updating the

~~estimated completion date and also updating which stages of the project had been completed (e.g., data extraction, data analysis).~~

2.1 Eligibility criteria

Studies were eligible if they reported primary quantitative or qualitative research relating to the impact of face covering use on adherence to other protective behaviours during the COVID-19 pandemic. Protective behaviours of interest included but were not limited to: COVID-19 physical/social distancing, hygiene (e.g., avoidance of face-touching, regular handwashing), staying at home, and reducing travel. Studies which examined the effectiveness and/or efficacy of wearing face coverings or factors related to adherence to face covering use were excluded. Published research and pre-publication articles were included. Reviews, position/discussion papers, conference abstracts, protocol papers, modelling studies, case reports and studies published in languages other than English were excluded (see Table 1).

Table 1. Inclusion criteria.

Inclusion Criteria	Exclusion Criteria
• Papers published from 1st~~1~~ January 2020 to present day at time of search (17th May 2022)<br>• All populations and all public settings other than healthcare settings<br>• Quantitative or qualitative research relating to the impact of face covering use on adherence to other protective behaviours during the COVID-19 pandemic<br>• Face covering use: i. All types of face covering, including (but not limited to) handmade and commercial cloth coverings (cloth, cotton, gauze,	• Healthcare settings<br>• Papers not in English<br>• Systematic reviews, narrative reviews, guidelines, position/discussion papers, conference abstracts, protocol papers, modelling studies and case reports.<br>• Studies which examined the effectiveness and/or efficacy of wearing face coverings or factors related to adherence to face covering use

etc), and medical coverings such as surgical face masks. ii. Face covering mandate.<br>•Experimental studies, observational studies, qualitative studies, laboratory studies, any other primary data, secondary data analyses.<br>• Published research and pre-publication articles	

2.2 Search strategy

A systematic search was conducted by UKHSA researchers on 17th May 2022 for papers from January 2020 until the date of searches. Sources searched included Ovid Medline, Ovid Embase, Ovid PsychInfo, Ovid EmCare, medRxiv preprints, Research Square and WHO COVID-19 Research Database. Search terms included terms related to COVID-19 (e.g. COVID-19, coronavirus, Sars-COV2), face coverings (e.g. mask, face cover, mouth covers) and protective behaviours (e.g., social distancing, handwashing, face-touching). A complete list of search terms is available in S1 Appendix. Forward and citation searches of included papers were also conducted on September 16th 2022, and eligible papers from January 1st~~1~~ 2020 to that date were also included.

2.3 Study identification

Selection and screening of papers followed a systematic search method following a Preferred Reporting Items for Systematic Reviews and Meta-Analyses (PRISMA) framework (14~~10~~). The study identification process is detailed in Fig 1 and contains full details of papers~~studies~~ included and excluded at each stage. ~~In the first stage~~Papers were initially title- and abstract-screened (decisions were made as to the eligibility of each paper following a review of their title and abstract alone). In the first stage of title and abstract screening, 10% of records were assessed in duplicate by two reviewers, and disagreements were resolved by discussion and consensus. In the second stage of title and abstract screening one reviewer assessed the remaining 90% of records. The screening tool Rayyan (15~~11~~) was used for both these first and second stages. All relevant records were then screened by full text~~-~~ by one reviewer (decisions were made as to

the eligibility of each paper following a review of the paper in full). ~~and~~These decisions were then checked by a second reviewer. There were five reasons for exclusion: (i) The direction of analysis was incorrect e.g., the study investigated behavioural predictors of face-covering use rather than vice versa (n = 5), (ii) the indepdent variables or outcome variables were not relevant to the research question (n = 14), (iii) the study did not comprise primary empirical research (n = 6), (iv) the paper was a previously undetected duplicate (n = 5), and (v) the paper was not written in English (n = 1).~~.~~ By the end of this process, 47 papers were deemed eligible and included in the review.

Fig 1. PRISMA 2020 flow diagram of the identification of studies process

2.4 Data extraction and synthesis

Data was sought for any relevant COVID-19 protective behaviour outcomes; at the completition of extraction, these comprised the following behavioural outcomes: physicial distancing, mobility, face-touching, hand hygiene, close contacts and generalised measures of protective behaviour. All results that were compatible with each of these outcomes were included.The following information was then collated for each study: country, publication status, population and sample size, methods, key outcome variables and main results. This was initially collated into one table which included an outcome variable column. This table was then divided by outcome variable into six smaller data extraction tables, and a narrative summary of results was produced, structured by these six outcome variable categories.

Data extraction was completed for each included study by one reviewer and independently checked by a second reviewer, with discrepancies resolved by discussion. Any evidence that was not directly relevant to the review question was not extracted.

2.5 Quality assessment

Quality including risk of bias was evaluated by one reviewer using the Quality Criteria Checklist (QCC) tool which can be used to assess the methodological quality of a study (16~~12~~). Using the QCC, studies were characterised as high, medium or low quality.

This checklist tool is composed of 10 questions, four of which are considered critical (questions on selection bias, group comparability, description of exposure/assessment of transmission routes, and

validity of outcome measurements); full details can be seen in S2 Appendix. A study was rated as high methodological quality if the answers were yes to the four critical questions plus at least one of the remaining questions. A study was rated as low methodological quality if answers were no to >50% of the 10 questions. Otherwise, the study was rated as medium methodological quality. In line with a systematic review which found that self-report methods of capturing adherence to protective behaviours can over-estimate objectively observed adherence by up to a factor of five (17~~13~~), any studies which employed a self-report methodology were deemed not to satisfy the validty of outcome criterion, and so were necessarily limited to a quality score of medium or below. An additional reviewer independently assessed five of the studies, and conflicting findings were discussed. Following discussions, a potential source of bias was identified in the first reviewer's application of one of the tool's questions, and all studies were subsequently re-evaluated to correct for this bias.

3. Results

3.1 Overview of search results

The initial searches generated 12,662 articles. After de-duplication, 7,980 records remained, and title and abstract screening resulted in 70 papers being accepted for full text screening. Following full text screening, 39 papers were accepted for inclusion in the review. Backwards and forwards citation searches were conducted which yielded a further 8 papers for inclusion, meaning the total number of papers included in the review was 47. Quality appraisal of the articles revealed that nine were of a high quality, 33 of a medium quality, and five of a low quality. ~~Finally, o~~Of the papers included nine were pre-prints, 35 were peer-reviewed publications, and three were published conference papers. Finally, it was found that some papers included more than one methodology, intervention or protective behaviour and thus, the number of studies described for each of these categories may exceed the total number of papers.

~~Note that in the paragraph that follows, frequency of studies are described for each of several categories, e.g., methodology employed, face-covering intervention investigated, protective behaviour investigated, and means of measurement employed (objective or self-report). The total of the n's in each category~~

~~always exceeds the total number of papers included (47). This is because some papers included more than one study, more than one methodology, or were concerned with more than one face-covering intervention or protective behaviour.~~

All studies included used quantitative methods, and none used qualitative methods. These included field experiments (n = 7), natural experiments (n = 14), lab experiments (n = 14), observational studies (n = 10) and cross-sectional studies (n = 9). Of these, 30 studies measured behaviour through observation, and 22 measured behaviour through self-report. Of the self-report studies, 10 were concerned with current or previous behaviour, and 12 with expected or intended behaviour. Though all included papers necessarily investigated the effect of face covering use on protective behaviours, the exact intervention of focus in each case was varied and fit into one of five~~5~~ categories: face covering use (self) i.e., the act of wearing a face covering oneself (n = 21); face covering use (other) i.e., in some way interacting with someone else wearing a face covering (n = 18); face covering use (self and other) i.e., in some way interacting with someone else who is wearing a face covering whilst also wearing a face covering oneself ~~face covering wearing on behalf of oneself and another person with whom one is interacting or is in proximity to~~ (n = 1); face covering policy i.e., the implementation of a face covering mandate (n = 19); and face covering intervention package i.e., a community programme designed to increase uptake of face covering usage (n = 1). The protective behaviour(s) in each case also varied, and in each case fit into one of 6 categories: physical distancing i.e., interpersonal distance (n = 30); mobility i.e., the extent to which people leave their home and visit public or residential spaces (n = 13); face-touching (n = 7); hand hygiene e.g., hand washing, avoidance of handshakes (n = 5); close contacts i.e., the extent to which people have close and extended interactions with people outside of their household (n = 4); and generalised protective behaviour i.e., a single measure to encapsulate two or more COVID-19 protective behaviours other than face-covering use, e.g, hand-washing, physical distancing and staying at home (n = 2). Full details on each paper can be seen in the data extraction tables in S3 Appendix.

Some studies initially appeared to meet the inclusion criteria but were ultimately excluded. For example, one study investigated how face covering use affected perceptions of physical distance between oneself and another person, however, since it did not investigate how face covering use affected distancing

behaviour, it was not deemed to be eligible for inclusion (18~~14~~). Similarly, another study investigated participants' judgements on how face covering use moderates the relationship between a given physical distance and transmission risk was not included since, although its focus was related to the review's objectives, it did not directly address it, in that it did not investigate how face covering use directly impacted adherence to protective behaviours (19~~15~~) .

The section below presents findings in relation to each of these six behaviours and describes the extracted data in terms of the effect that face covering use has on each type of behaviour, also exploring any patterns with regards to how the effect of face covering use varies by the type of intervention, and the type of study design.

3.2 Physical distancing (see table 2a in S3 Appendix for full details)

Outcomes were mixed and varied depending on the type of study. Findings from the majority of lab experiments (10 of 13) indicated that physical distancing was lower in conditions where a face covering was used by another person compared with conditions where it was not (20-27~~16-23~~), (28~~24~~: two studies), and two of these studies also found that physical distancing was lower when a face covering was used by the self (28~~24~~: two studies). Additionally, one of these studies investigated how physical distancing in conditions with and without face covering use compared with a baseline measure (that is, physical distancing outside the context of face covering use) (20~~16~~). It was found that physical distancing was significantly greater than the baseline in conditions without face covering use, but not significantly different in conditions with face covering use, potentially suggesting that differences between conditions is driven by participants moving away from people without face coverings (relative to where they would normally position themselves), rather than towards people with face coverings. Of the remaining three studies, one found that physical distancing was lower in conditions of face covering use (other), but that this effect was conditional on situational factors such as effort required to maintain physical distance, and age of the other person (29~~25~~), and two found that distancing was greater in the case of face covering use (other) (30~~26~~), (31~~27~~: Study 2).

In contrast, the majority of field experiments (5 of 6) found that physical distancing was either greater or not significantly different in conditions where face coverings were used, compared to conditions where they were not. Two studies found that physical distancing was greater in conditions where face coverings (self or other) were used compared to when they were not (32~~28~~), (31~~27~~: Study 1), with a third study finding that physical distancing was greater in areas where a face covering intervention package had been implemented compared to those where it had not (33~~29~~). One study found mixed effects, finding that face covering use (self) had no effect on distancing, but that distancing was greater in conditions of face covering use (other) (34~~30~~), while another study found no differences in distancing between conditions of face covering use (self and other) and conditions without face covering use (35~~31~~). A minority of field experiments (1 of 6) found that physical distancing was lower in conditions of face covering use (other), but that this effect was conditional on gender of participant, race and social status of confederate, and face covering policy implemented at the time (36~~32~~). Additionally, three~~two~~ of the above studies also investigated through a natural design whether the effect of face covering use on physical distancing differed between different time periods which varied by the face covering~~mask~~ policy (mandatory or voluntary) implemented at the time. One study found that face-covering use by others significantly reduced physical distancing adherence amongst men only, both in mandatory and in voluntary face covering policy contexts; the effect was slightly more pronounced in the mandatory context (36). The two other studies found no difference between policy contexts; both found that face covering use (other) increased physical distancing both when it was mandated and when it was voluntary (32, 34).~~one found that a distance-reducing effect of face covering use was stronger when a face covering use was mandated (but only for males) (32), whilst a second did not find a significant interaction between face covering policy and face covering use (30).~~

The four natural experiments explored the impact of face covering policy on physical distancing. Three of these found that physical distancing did not significantly differ according to whether face covering wearing was voluntary or mandatory (37~~33~~: Study 2), (28~~24~~: Study 2), (34~~30~~), while a third found that the impact of face covering policy on physical distancing was dependent on context; attention given to physical distancing decreased when a face covering mandate was introduced on public transport, but increased

when the mandate was expanded to bars and restaurants, and again when expanded to all public spaces (3834).

The four observational studies found either a positive or a neutral relationship between face covering use or face covering policy and physical distancing. Three observational studies found no significant relationship between face covering use (self) or face covering policy and physical distancing (3733: two studies), (3935), while the fourth observational study found that physical distancing was greater in those who wore face coverings, and also that it was greater when a face covering mandate was implemented (4036).

Four of the six correlational studies found that as self-reported face covering use (self or other) increased, so did self-reported physical distancing (41-4437-40). One study showed mixed results for the relationship between face covering use and physical distancing, finding that those who reported wearing face coverings all the time were more likely to report adherence to physical distancing compared with those who reported never doing so, but those who reported wearing face coverings sometimes were more likely to report having reduced distancing with people than those who reported never doing so (4541). The final correlational study found no relationship between face covering use (self) and physical distancing (4642).

3.3 Mobility (see table 2b in S3 Appendix for full details)

Of the 10 natural experiments, five5 found that when face covering policies were in place mobility increased (i.e., people were less likely to stay at home) (47-5143-47), four4 found that mobility was unchanged, (52-5548-51) and one found that mobility decreased (i.e., people were more likely to stay at home) (5652). The lab experiment found that mobility is likely to increase when policies are in place, as participants reported being more willing to use the London Underground when a face covering mandate was in operation (5753).

Of the two cross-sectional studies, one found that self-reported adherence to personal protective behaviours, which included face covering use (self) predicted self-reported frequency of going out in public, and that this relationship was mediated by belief in ‘substitution myths’; the belief that one protective behaviour can be substituted for another, with risk staying constant (4642). The second cross-sectional

study found that those who reported always wearing a face covering outside their home were less likely to report visiting a friend, neighbour, bar or club, compared with those who did not report always wearing a face covering outside their home (44~~40~~).

3.4 Face-touching (see table 2c in S3 Appendix for full details)

The field experiment found that face covering use (self) resulted in less face-touching; there was a significant increase in the number of individuals who touched their eyes or hair when they were not wearing a face covering, and, when investigating areas not covered by a face covering (hair, forehead, eyes and ears) the absolute number of total touches was significantly higher in those not wearing one (58~~54~~). Two of the six observational studies found similar results; those who wore face coverings were less likely to touch their face (59~~55~~, 60~~56~~). Two more observational studies found that results were dependent on the operationalisation of face-touching; when face-touching was defined as making contact with any part of the face (including touches to the mask), there was no significant relationship between face covering use (self) and face-touching, but when it was defined as making contact with specific parts of the face (not including touches to the mask), those who wore face coverings touched their faces less frequently (61~~57~~: two studies). A minority of observational studies (1~~one~~ of 6) found a greater frequency of face-touching in those who wore coverings compared with those who did not (62~~58~~). The final observational study found that face covering policy had no impact on face-touching; there was no statistically significant association between the face covering policy in operation and frequency of face-touching (63~~59~~).

3.5 Hand hygiene (see table 2d in S3 Appendix for full details)

The natural experiment found that face covering policy had no impact on hand hygiene; attention paid to hygiene practices was not significantly different according to the implementation of different face covering policies (38~~34~~). The three cross-sectional studies found that those who reported wearing face coverings more often were more likely to report also adhering to hand hygiene practices (41~~37~~, 44~~40~~, 45~~41~~).

3.6 Close contacts (see table 2e in S3 Appendix for full details)

One of the two natural experiments found that number of close contacts was significantly lower when a face covering mandate was expanded from public transport to include restaurants and bars, and lower again when expanded to all public spaces (38~~34~~). The other natural experiment found that a face covering mandate did not affect whether participants cancelled or postponed personal or social activities (55~~51~~).

One of the three cross-sectional studies found that the relationship between face covering use (self) and number of close contacts was mixed; those who reported wearing face coverings all the time were more likely to report spending less than 15 minutes in close contact with someone compared with those who reported never wearing face coverings, while those who reported sometimes wearing face coverings were more likely to report spending over 60 minutes in close contact with someone compared with those who reported never doing so (45~~41~~). The second cross-sectional study found that those who did not always wear face coverings outside work were more likely to have close contact with people they do not live with (44~~40~~), whilst the final cross-sectional study found that self-reported face covering use outside of work significantly predicted a greater number of self-reported daily contacts (64~~60~~).

3.7 Generalised protective behaviour (see table 2f in S3 Appendix for full details)

The term ‘generalised protective behaviour’ was used to categorise any studies whose outcome variable was a single measure which encapsulated two or more other COVID-19 protective behaviours besides face covering use e.g., hand washing, physical distancing and staying at home. Both studies found that self-reported face covering use (self) was associated with self-reported behaviours that are likely to reduce the spread of COVID-19. In one study the majority of participants reported that their adherence to other protective behaviours was either unchanged or greater when they wore a face covering compared with when they did not (65~~61~~), while the other study found that self-reported face covering use (self) was associated with self-reported reduced engagement with COVID-19 related risky behaviour (66~~62~~).

3.8 Quality assessment

Few clear patterns were found when analysing the results of studies cross-sectionally by quality. With the exception of face-touching, for all behaviours (physical distancing, mobility, hand hygiene, close contacts and generalised protective behaviour) study quality was mixed for those that found positive, negative, neutral or equivocal effects of (or associations with) face covering use. For face-touching, studies were either of a low or medium quality, with all four studies of medium quality finding the same result: a negative effect of (or association with) face covering use. The three studies of low quality were mixed: one found a positive association, one found a negative association and one found a nonsignificant association.

4. Discussion

Although face coverings have been found to reduce the transmission of airborne diseases, concerns have been raised as to the effect that their use may have on adherence to other COVID-19 protective behaviours. While several studies which explore this issue have been conducted, they had not previously been brought together in a review. This systematic review has identified and summarised the findings of 47 papers which collectively investigate the effect of face covering use on other COVID-19-related protective behaviours, namely, physical distancing, mobility, face-touching, hand hygiene, close contacts and generalised protective behaviour. Findings showed that results varied considerably, and that the nature of the variation often depended upon the specific behaviour investigated and the type of study employed to investigate it. The sections below, organised by behaviour, summarise and discuss the findings.

4.1 Physical distancing

One of the clearest patterns emerging from the studies relating to physical distancing was the distinction between the respective findings of lab experiments and field experiments. The majority of lab experiments found that physical distancing was lower in conditions of face covering use (compared with conditions without face covering use), whereas the majority of field experiments found that physical distancing was either greater or unchanged in conditions of face covering use.

A number of considerations should be made when interpreting these results, starting with the respective merits of field and lab experiments. There are three main advantages of lab experiments. First, the majority of the lab experiments employed a within-subjects design, meaning that individual differences which may impact on physical distancing (e.g., age, gender, risk perception, conscientiousness) are essentially controlled for in a way that cannot be done with between-subjects field experiments (67) (although estimates~~ions~~ of such variables, e.g. age, were~~was~~ in some cases recorded and controlled for in the field experiments). Second, the lab experiments typically allowed for more precision in data collection. The majority of lab experiments~~ typically~~ used an on-screen slider for participants to indicate their preferred distances, or a virtual reality environment in which computers captured the distances participants maintained from virtual agents. In contrast, some of the field experiments employed human judgement to determine whether a physical distancing guideline had been violated, increasing the risk of bias. However, four of the six field experiments used some form of electronic sensor to collect data (all of which found that physical distancing was greater or unchanged in conditions of face covering use). Third, lab experiments are able to examine the impact of face covering use on physical distancing in a context which is agnostic of face covering policy (mandatory vs voluntary), in contrast to field experiments which are necessarily conducted under periods of either mandatory or voluntary face covering wear~~. ~~(the potential for an interaction between face covering use and face covering policy is discussed in more detail in subsection 4.7). ~~The impact of face covering use on other protective behaviours may be related to whether face covering use is mandatory or voluntary. For example, during periods of voluntary face covering wear the wearing of a face covering may act as a social cue to keep a greater distance (8); however, face covering wear may not act as the same social cue during periods of mandatory face covering wear (since all will be wearing face coverings). The ability to examine behaviour in a context which is agnostic of face covering policy may therefore be an advantage.~~

However, whilst field experiments are less controlled than lab experiments, they are more ecologically valid (68). A key advantage of field experiments compared to lab experiments is that the disease transmission risk is real, not hypothetical; therefore a person's approach to physical distancing has a real impact on their own risk and on others' risk of contracting and spreading COVID-19. In all but one of the

six field experiments not only were participants blinded to the experimental condition they were assigned to, but they were also unaware that they were taking part in an experiment at all, and so responses were unlikely to biased by, for example, demand characteristics. Behaviour captured in field experiments may therefore be more representative of and generalisable to wider populations than that collected during lab experiments, in which participants could second-guess research objectives, potentially causing demand effects~~characteristics~~ (67). Additionally, in the majority of lab experiments, rather than being measured for actual behaviour, participants were asked to indicate distances they would prefer from the avatars or characters, allowing them to make a conscious decision which may or may not be indicative of how they might behave in real-life scenarios.

In addition to the lab and field experiments, the effect of face covering use on physical distancing was also investigated through a number of different methodologies; the majority of natural and observational studies tended not to find any significant associations between face covering use (or face covering policy) and physical distancing, and cross-sectional studies tended to find positive associations between self-reported face covering-wearing and physical distancing. These different study types also have their respective strengths and weaknesses which should be considered when interpreting the results. The observational studies are necessarily high in ecological validity, however are less controlled (69). As such, the means of measurement (human judgement) may be lacking in precision and subject to bias, and furthermore, only claims of association (and not causality) can be made (70). For example it is likely that relationships between face covering-wearing and physical distancing are driven by confounding variables (e.g., risk perception, age, conscientiousness) impacting on both behaviours. The natural experiments, though again high in validity, are types of observational studies and so are subject to the same qualifications. Cross-sectional studies are also unable to draw claims of causality due to the likely impact of confounding variables (70), and may also be limited in their precision since measures are self-reported and not necessarily indicative of real-life behaviour (17).

A final consideration that should be made when interpreting the results is to consider the nature of the intervention in each case. Specifically, consideration should be given to: whether the independent variable (IV) is face covering condition of the self, face covering condition of another person, face covering policy,

or some combination of any of the three; and the way in which physical distancing is operationalised in each case. Whilst there is no clear pattern with regards to how these different interventions and operationalisations of distancing impact outcomes, it is important to consider such differences when interpreting findings. Overall, it is clear that there is no consensus in relation to the impact of face covering use on physical distancing and that further research is required to bring the merits of different study types together and capture more reliable, and perhaps more consistent findings with regards to the effect of face covering use on physical distancing.

4.2 Mobility

The findings on mobility are also inconclusive, although there is more evidence to suggest that face covering policies increase mobility than there is to suggest that they decrease mobility. Natural experiments tended to compare changes in mobility from a baseline between areas and periods of time that varied according to the face covering policy implemented. The findings arising from such studies are therefore likely to be confounded by any number of other variables which are likely to impact upon mobility (e.g., other COVID-19 public health measures, rate of infection at time/area of data collection) and claims of causality must be made with caution. Generally speaking, however, these studies employ rich data sources and complex statistical models to account for these variables as far as is possible, and so conclusions made from these studies can be arrived at with a certain level of confidence. If it is the case that implementing face covering policies increases mobility, a plausible explanation may be that a mandate instils confidence in people that if they are to leave home others are more likely to wear a face covering, leading to a greater sense of security. Further qualitative work could be conducted to investigate how face covering policies impact upon people's decision to leave home and move around their communities.

Two cross-sectional self-report studies which investigated how face covering use by the self impacted on mobility were inconclusive, with relationships found in both directions (4440, 4642). Interestingly however, in one case it was found that a positive relationship between self-reported adherence to personal protective behaviour (including wearing a face covering) and going out in public with people outside ones' household was mediated by a belief that one protective behaviour can be safely substituted for another (4642), providing evidence in favour of the risk compensation hypothesis.

4.3 Face-touching

The impact of face covering use on face-touching was the most consistent of all the behavioural outcomes, with a general trend for face-touching to be lower in conditions of face covering use than in conditions without. This was found in a field experiment (5854) and four of six observational studies investigating the relationship between face covering use by the self and face-touching (5955, 6056), (6157: two studies). Whilst observational studies are subject to the same caveats described in the physical distancing subsection the general pattern of a negative relationship between face touching and face covering use suggests that even if risk compensation is at play, it is not strong enough to result in a positive relationship. Furthermore, that this pattern is corroborated by the experimental study boosts the claim that face covering use might reduce face touching behaviour. Additionally, all of the relatively higher quality studies investigating face-touching (which were all of a medium, as opposed to a low quality) found face covering use to have a negative effect on (or a negative association with) face-touching. It should however be noted that different operationalisations of face-touching were employed across face-touching these studies, and that in cases where significant effects or associations were found, they did not always apply to all parts of the face. Further research using experimental designs is required in this area, but on the basis of the studies reviewed to date, it seems reasonable to conclude that face covering use reduces instances of face-touching.

4.4 Hand hygiene

Across four studies (one natural experiment and three cross-sectional studies), three studies found a positive relationship between either face covering use or face covering policy and hand hygiene practices and a fourth found no significant relationship. The three studies which found positive relationships were cross-sectional (4137, 4440, 4641) and based on self-report surveys; findings therefore show that those who report wearing face coverings are more likely to also report practicing good hand hygiene, but the cross-sectional nature of the studies means a causal relationship cannot be established. Furthermore, these studies can only inform us on how the isolated practices of wearing a face covering and practising hand hygiene relate to one another and cannot tell us whether hand hygiene is affected whilst wearing a

face covering. The natural experiment (3834), which employed face covering policies as an IV and found no significant effect on self-reported attention paid to hygiene practices may be more informative, suggesting that implementing a face covering mandate has no ‘collateral’ effect on hand hygiene practices. It should be noted however that this study is also subject to the caveats around accuracy associated with self-report data. In summary, the literature on the effect of face covering use on hand hygiene is limited and relies on self-report data, possibly because hand hygiene is a relatively private practice that is difficult to investigate using an experimental or observational design. On the basis of the literature reviewed however, it can be said that there is no evidence thus far to suggest that face covering use causes a reduction in hand hygiene practices.

4.5 Close contacts

There was little consensus in the literature on close contacts. Across two natural experiments, it was found that face covering mandates either had no effect on close contacts, or that they reduced them (3834, 5551). Across three cross-sectional studies the results were mixed with both positive and negative relationships found (4440, 4541, 6460). It is therefore difficult to draw robust conclusions about the impact of face covering use on close contacts.

4.6 Generalised protective behaviour

Two cross-sectional studies (6561, 6662) investigated the relationship between face covering use by the self and generalised adherence to other protective behaviours and found a relationship between face covering use and increased adherence to other protective behaviours (or reduced undertaking of risky behaviours). As previously discussed, cross-sectional studies do not allow for claims of causality to be made, and confounding variables are likely to influence any relationships.

4.7 Risk compensation outcomes under different policies

An area for consideration is whether the risk compensation outcome varies according to whether face covering use is voluntary or mandatory. For example, it might be expected that when face covering use is voluntary, there is a stronger relationship between face covering use (i.e. self-selected face covering use)

and adherence to other protective behaviours given that the decision to wear a face covering is more likely to be driven by one's own risk perception rather than the extrinsic factor of the mandatory face covering policy. Equally, when face covering use is voluntary, their use may serve as a stronger social signal as to the preferences or condition of the person using them since others know that they are doing so despite the lack of a mandate. For example, voluntary use may signal that the user is particularly risk-averse and therefore others may be more likely to keep their distance out of consideration and respect. Alternatively, voluntary use may signal that the user is more likely to be infectious, and so others may be more likely to keep their distance for self-protective reasons.

In the present review, only three included studies compared the effect of face covering use across different face covering policies (mandatory or voluntary) and only through a natural design and for one protective behaviour (physical distancing). In all three studies, the effect of face covering use by others on physical distancing adherence was not influenced by policy context, although in one study the effect was reportedly slightly more pronounced in the mandatory than in the voluntary context. There were also 24 studies in which, although the authors did not assess impact of face covering policy, they did provide information on the real-world policy implemented during the time of study; that is whether face covering use was mandatory, voluntary or whether the policy was varied (i.e., that for some of the duration of the study it was mandatory, and for some of the time it was voluntary). Thirteen focused on physical distancing (five carried out in a mandatory and six in a voluntary face covering context) and results across studies were mixed in both contexts. Two focused on mobility and in both cases face covering use was voluntary but a positive association was found in one and a negative association in the other. There were three studies focused on face-touching and three on hand hygiene. For both protective behaviours face covering policy varied across studies but the face-touching studies all reported negative effects or associations and the hand hygiene studies all reported positive effects or associations. Two studies were focused on close contacts, one of which was conducted under mandated face covering use and found the association to be equivocal, and one of which was conducted when face covering use was voluntary and found the association to be negative. A final study that focused on generalised protective behaviour was conducted under varied policies and found a positive association.

It is difficult to draw robust conclusions about any interaction between face covering use and face covering policy from studies included in this review. A particular limitation of the literature is that no study to date has applied type of face covering policy (mandatory or voluntary) as an independent variable to assess its interaction with face covering use on risk compensation for other protective behaviours. This is an evidence gap that should be addressed in future research.

4.8 Limitations

This review is novel in drawing together current literature on the impact of face covering use on other protective behaviours during the COVID-19 pandemic, and contributing to an understanding of risk compensation in this context. However, some limitations should be acknowledged when considering this review's findings. First, despite every attempt to apply the inclusion and exclusion criteria systematically and objectively, it is likely that some level of subjectivity may have impacted on paper screening and selection. To mitigate against this, 10% of the title and abstract screening was undertaken by two researchers, and any disagreements resolved by discussion and consensus. The full-text screening was also checked by a second researcher. Second, due to resource constraints (namely, the linguistic limitations of the researchers), only papers published in English were included in the review, inevitably creating a geographical bias. For example, 32 of the 47 papers described studies carried out in the UK, Western Europe, Canada, North America or Australia.

4.9 Conclusion

Overall, findings relating to the impact of face covering use or policy on other protective behaviours are inconsistent, varying both according to the particular behaviour in question, and by study type. Evidence relating to the potential impact of risk compensation is also inconsistent; while some studies suggest risk compensation may play a role in shaping behaviour, others suggest it has no impact. Findings are particularly inconsistent in relation to physical distancing, with considerable discrepancy found between study types. Whilst assessments of behaviour in real-life settings tend to find that face covering use either increases or has no impact upon physical distancing, lab experiments tend to suggest that physical distancing decreases in conditions of face covering use. There is some suggestion that the impact of face

covering use on physical distancing could be affected by whether or not mandatory face covering policies are in effect (which would indicate that conclusions drawn from a voluntary context should not be directly applied to a mandatory context and vice versa) but findings in this area are mixed and further assessment of this moderating effect is warranted. Of the other behaviours, findings (though mixed) tend to suggest that face covering mandates may increase mobility and that face covering use may decrease face touching. While some studies suggest a relationship between face covering use and increased hand hygiene practices and adherence to other protective behaviours, these studies are predominately cross-sectional and rely on self-report measures, and do not allow conclusions to be drawn about the impact of face covering use on adherence to these behaviours. Findings relating to the impact of face covering use on close contacts is mixed, and further research is needed.

Overall, this review highlights that there is no clear consensus in the literature as to whether face covering use improves adherence to other protective behaviours, reduces adherence to other protective behaviours, or has no impact. There are two key recommendations which can be made from this review: 1) further research, using more robust research designs, is needed to establish whether face covering use affects different types of protective behaviours; and 2) any policy decisions made in relation to recommended or mandatory face covering use must take into account the inconsistencies and caveats in the evidence base, recognising that current evidence does not allow firm conclusions to be drawn about the potential impact of face covering use on adherence to other protective behaviours. Recommendations for future research are summarised below.

4.8.1 Recommendations for future research

- Further field experiments (or more ecologically valid lab experiments) investigating the effect of face covering use on physical distancing. Such studies might make a particular focus of investigating:
    - Differing effects of face covering use by the self and face covering use by another person
    - How different face covering policies might moderate the relationship between face covering use and physical distancing . For example, a field experiment could be conducted whereby two manipulations occur; one whereby the setting varies according to the face covering

policy implemented (mandatory or voluntary), and one whereby a confederate appears either wearing or not wearing a face covering. Physical distancing from the confederate would then be measured. This would allow for the direct comparison between the moderating effects of voluntary and mandatory settings with more control over extraneous factors.

- How effects might vary according to the operationalisation of distancing; for example, whether the absolute distance is measured, or whether a binary variable capturing whether or not a particular distance or guideline is adhered to is captured

- Lab experiments replicating those cited in this review, but which vary by the framing of physical distancing (i.e., self-protective or prosocial)
- Qualitative research investigating how differing face covering policies affect attitudes towards leaving the home and moving around the community
- Experimental studies investigating the effect of face covering use and face covering policies on face-touching, hand hygiene and close contacts

5. Acknowledgements

The authors thank Dr Joanna Milward for contributing to the development of the protocol and search terms, Nicola Pearce-Smith for creating the search strategies and running the searches themselves, as well as providing guidance on screening, and Megan Phillips for contributing to the quality appraisal as a second reviewer.

6. References

1. Ayouni I, Maatoug J, Dhouib W, Zammit N, Fredj SB, Ghammam R, et al. Effective public health measures to mitigate the spread of COVID-19: a systematic review. BMC Public Health. 2021;21:1-14.

2. The Royal Society and the British Academy. Face masks and coverings for the general public: behavioural knowledge, effectiveness of cloth coverings and public messaging. London, UK: The Royal Society; 2020.

~~3. Jefferson T, Dooley L, Ferroni E, Al-Ansary L, van Driel M, Bawazeer G, et al. Physical interventions to interrupt or reduce the spread of respiratory viruses. Cochrane Database of Systematic Reviews. 2023(1):CD006207.~~

~~4. Li Y, Liang M, Gao L, Ahmed M, Uy J, Cheng C, et al. Face masks to prevent transmission of COVID-19: a systematic review and meta-analysis. American Journal of Infection Control. 2021;49(7):900-6.~~

35. World Health Organization. Advice on the use of masks in the context of covid-19: interim guidance. 2020. pp 1-5. https://apps.who.int/iris/bitstream/handle/10665/331693/WHO-2019-nCov-IPC_Masks-2020.3-eng.pdf?sequence=1&isAllowed=y

4. Peltzman S. The effects of automobile safety regulation. Journal of Political Economy. 1975 Aug 1;83(4):677-725.

5. Hedlund J. Risky business; safety regulations, risk compensation, and individual behaviour. Injury Prevention. 2000 Jun 1;6(2):82-9.

6. Wilde GJ. The theory of risk homeostasis: implications for safety and health. Risk Analysis. 1982 Dec;2(4):209-25.

76. Greenhalgh T. Face coverings for the public: laying straw men to rest. Journal of Evaluation in Clinical Practice. 2020;26(4):1070-7.

87. Martin G, Hanna E, Dingwall R. Urgency and uncertainty: covid-19, face masks, and evidence informed policy. BMJ. 2020;369:m2017.

98. Mantzari E, Rubin G, Marteau T. Is risk compensation threatening public health in the covid-19 pandemic? BMJ. 2020;370:m2913.

10. Henk K, Rosing F, Wolff F, Frenzel SB, van Dick R, Erkens VA, et al., An examination and extension of the Peltzman effect during the Covid-19 pandemic. Current Research in Ecological and Social Psychology. 2023;4:100091.

11. Jones Ritten C, Thunström L, Cherry T, Wulfhorst JD. Frequent mandatory COVID-19 testing may increase risk behaviour. PNAS Nexus. 2022 Nov;1(5):pgac247.

12. Ludema C, Rosenberg MS, Macy JT, Kianersi S, Luetke M, Chen C, et al. Does receiving a SARS-CoV-2 antibody test result change COVID-19 protective behaviors? Testing risk compensation in undergraduate students with a randomized controlled trial. PLoS ONE. 2022 Dec 20;17(12):e0279347.

139. Millest A, Carter H. Effect of face-covering use on adherence to other Covid-19 protective behaviours. PROSPERO 2022 CRD42022331961 2022 [Available from: https://www.crd.york.ac.uk/prospero/display_record.php?ID=CRD42022331961.

140. Page MJ, McKenzie JE, Bossuyt PM, Boutron I, Hoffmann TC, Mulrow CD, et al. The PRISMA 2020 statement: an updated guideline for reporting systematic reviews. BMJ. 2021;371:n71

151. Ouzzani M, Hammady H, Fedorowicz Z, Elmagarmid A. Rayyan—a web and mobile app for systematic reviews. Systematic Reviews. 2016;5(1):210.

162. Academy of Nutrition and Dietetics. Evidence analysis manual: steps in the academy evidence analysis process. 2016. [cited 2023 Mar 03]. Available from https://www.andeal.org/evidence-analysis-manual.

173. Davies R, Mowbray F, Martin A, Smith L, Rubin G. A systematic review of observational methods used to quantify personal protective behaviours among members of the public during the COVID-19 pandemic, and the concordance between observational and self-report measures in infectious disease health protection. BMC Public Health. 2022;22(1):1436.

1814. Chakraborty S, Stefanucci J, Creem-Regehr S, Bodenheimer B. Distance estimation with social distancing: a mobile augmented reality study. In 2021 IEEE International Symposium on Mixed and Augmented Reality Adjunct (ISMAR-Adjunct); 2021 October 04-08; Bari, Italy. IEE; 2021. p. 87-91.

1915. Svenson O. People underestimate the change of airborne corona virus exposure when changing distance to an infected person: on interpersonal distance, exposure time, face masks and perceived virus exposure. medRxiv [Preprint]. 2022 [cited 2023 Mar 03]. Available from http://medrxiv.org/content/early/2022/03/15/2022.03.14.22272341.abstract.

2016. Biggio M, Bisio A, Bruno V, Garbarini F, Bove M. Wearing a mask shapes interpersonal space during COVID-19 pandemic. Brain Sciences. 2022;12(5):682.

21~~17~~. Cartaud A, Quesque F, Coello Y. Wearing a face mask against Covid-19 results in a reduction of social distancing. PLoS ONE. 2020;15(12):e0243023.

22~~18~~. Gulbetekin E. Effects of mask use and race on face perception, emotion recognition, and social distancing during the COVID-19 pandemic. Research Square [Preprint]. 2021 [cited 2023 Mar 03]. Available from https://assets.researchsquare.com/files/rs-692591/v1/1cab9894-2761-4622-b8b9-6d829c3d4343.pdf?c=1631886459.

23~~19~~. Iachini T, Frassinetti F, Ruotolo F, Sbordone F, Ferrara A, Arioli M, et al. Psychological and situational effects on social distancing and well-being during the COVID-19 pandemic: not a question of real risk. PsyArXiv [Preprint]. 2020 [cited 2023 Mar 03]. Available from https://psyarxiv.com/r7usv/.

24~~0~~. Lee Y, Chen Y. Influence of wearing surgical mask on interpersonal space perception between mainland Chinese and Taiwanese people. Frontiers in Psychology. 2021;12:692404.

25~~1~~. Lisi MP, Scattolin M, Fusaro M, Aglioti SM. A Bayesian approach to reveal the key role of mask wearing in modulating projected interpersonal distance during the first COVID-19 outbreak. PLoS ONE. 2021;16(8):e0255598.

26~~2~~. Xiong W, Wang, C., & Yu, X. The effect of face mask and approach pattern on interpersonal distance in COVID-19 pandemic using VR technology. In: Long S, Dhillon BS, editors. International Conference on Man-Machine-Environment System Engineering; 2022 Oct 21-23; Beijing, China. Singapore: Springer; 2022. p. 92-8.

27~~3~~. Wang M, Lee Y-C. Investigating the effects of face Mask and gender on interpersonal distance judgments. In: Long S, Dhillon BS, editors. Man-Machine-Environment System Engineering: Proceedings of the 21st International Conference on MMESE; 2021 Oct 23-25; Beijing, China. Singapore: Springer; 2022. p. 40-5.

28~~24~~. Luckman A, Zeitoun H, Isoni A, Loomes G, Vlaev I, Powdthavee N, et al. Risk compensation during COVID-19: The impact of face mask usage on social distancing. Journal of Experimental Psychology: Applied. 2021;27(4):722-38.

29~~25~~. Kroczek LOH, Bohme S, Muhlberger A. Face masks reduce interpersonal distance in virtual reality. Scientific Reports. 2022;12(1):2213.

3026. Bühler MA, Lynch SD, Bhojwani T, Zidan A, Fiset F, McFadyen BJ, et al. Stay out of the blast radius: influence of surgical masks on virtual pedestrian interactions. Research Square Research Square [Preprint]. 2021 [cited 2023 Mar 03]. Available from https://assets.researchsquare.com/files/rs-852079/v1/3e95f3ec-8eaf-46db-85df-e5283756a6ca.pdf?c=1638763153

3127. Seres G, Balleyer AH, Cerutti N, Danilov A, Friedrichsen J, Liu YM, et al. Face masks increase compliance with physical distancing recommendations during the COVID-19 pandemic. Journal of the Economic Science Association. 2021 Nov 13;7:139-58.

3228. Marchiori M. COVID-19 and the social sistancing paradox: dangers and solutions. arXiv [Preprint]. 2020 [cited 2023 Mar 03]. Available from https://arxiv.org/pdf/2005.12446.pdf?fbclid=IwAR3edT8vzdd_Rt8ykeVC8Y0RQ1hLs4A9Buny6d3hYc2TZqD5huEg5J478U4

3329. Abaluck J, Kwong LH, Styczynski A, Haque A, Kabir MA, Bates-Jefferys E, et al.. Normalizing community mask-wearing: a cluster randomized trial in Bangladesh. National Bureau of Economic Research;2021 Apr. Report No.: 28734.

3430. Seres G, Balleyer A, Cerutti N, Friedrichsen J, Suer M. Face mask use and physical distancing before and after mandatory masking: No evidence on risk compensation in public waiting lines. Journal of Economic Behavior & Organization. 2021;192:765-81.

3531. Blanken TF, Tanis CC, Nauta FH, Dablander F, Zijlstra BJH, Bouten RRM, et al. Promoting physical distancing during COVID-19: a systematic approach to compare behavioral interventions. Scientific Reports. 2021;11:19463.

362. Aranguren M. Face mask use conditionally decreases compliance with physical distancing rules against COVID-19: gender differences in risk compensation pattern. Annals of Behavioral Medicine. 2022;56:332-46.

373. Liebst LS, Ejbye-Ernst P, de Bruin M, Thomas J, Lindegaard MR. No evidence that mask-wearing in public places elicits risk compensation behavior during the COVID-19 pandemic. Scientific Reports. 2022;12:1511.

38~~34~~. Jørgensen F, Lindholt MF, Bor A, Petersen MB. Does face mask use elicit risk-compensation? Quasi-experimental evidence from Denmark during the SARS-CoV-2 pandemic. European Journal of Public Health. 2021;31(6):1259-65.

39~~35~~. Puttock EJ, Marquez J, Young DR, Shirley AM, Han B, McKenzie TL, et al. Association of masking policies with mask adherence and distancing during the SARS-COV-2 Pandemic. American Journal of Infection Control. 2022;50:969-74.

40~~36~~. Atzema CL, Mostarac I, Button D, Austin PC, Javidan AP, Wintraub L, et al. Assessing effective mask use by the public in two countries: an observational study. BMJ Open. 2021;11:e049389.

41~~37~~. Betsch C, Korn L, Sprengholz P, Felgendreff L, Eitze S, Schmid P, et al. Social and behavioral consequences of mask policies during the COVID-19 pandemic. Proceedings of the National Academy of Sciences of the United States of America. 2020;117(36):21851-3.

42~~38~~. Lama Y, Budenz A, Gaysynsky A, Iles IA, Chou WYS. Factors associated with COVID-19 behavioral intentions: findings from an online survey. American Journal of Health Promotion. 2022;36(7);1183-92.

43~~39~~. Li M, Colby H. Association between actual and perceived U.S. COVID-19 policies and preventive behavior. Annals of Behavioral Medicine. 2021;55:369-75.

44~~0~~. Sheehan MM, Pfoh E, Speaker SL, Rothberg M. Changes in social behavior over time during the COVID-19 pandemic. Cureus. 2020 Oct 01;12(10):e10754.

45~~1~~. Doung Ngern P, Suphanchaimat R, Panjangampatthana A, Janekrongtham C, Ruampoom D, Daochaeng N, et al. Associations between wearing masks, washing hands, and social distancing practices, and risk of COVID-19 infection in public: a cohort-based case-control study in Thailand. medRxiv [Preprint]. 2020 [cited 2023 March 03]. Available from https://www.medrxiv.org/content/10.1101/2020.06.11.20128900v4

46~~2~~. Griggs A, Weaver J, Alvarado J. Masking our risky behavior: how licensing and fear reduction reduce social distancing behavior. Journal of Social Psychology. 2022 Sep 3;162(5):607-20. Epub 2021 Jul 27.

47~~3~~. Fracalossi de Moraes R, Russell LB, Santos da Silva LL, Toscano CM. Effects of non-pharmaceutical interventions on social distancing during the COVID-19 pandemic: evidence from the 27 Brazilian states. PLoS ONE. 2022;17(3):e0265346.

48~~44~~. Hosseini MS, Gittler AM. Factors influencing human mobility during the COVID-19 pandemic in selected countries of Europe and North America. 2020 IEEE International Conference Big Data (Big Data); 2020 Dec 10-13; Atlanta, GA, USA. IEE; 2021. p. 4866-72.

49~~45~~. Wadud Z, Rahman SM, Enam A. Face mask mandates and risk compensation: an analysis of mobility data during the COVID-19 pandemic in Bangladesh. BMJ Global Health. 2022;7:e006803.

50~~46~~. Wang F, Ge, X, Huang D. Government intervention, human mobility, and COVID-19: a causal pathway analysis from 121 countries. Sustainability. 2022;14:3694.

51~~47~~. Yan Y, Bayham J, Richter A, Fenichel EP. Risk compensation and face mask mandates during the COVID-19 pandemic. Scientific Reports. 2021;11:3174.

52~~48~~. Barros V, Manes I, Akinwande V, Cintas C, Bar-Shira O, Ozery-Flato M, et al. A causal inference approach for estimating effects of non-pharmaceutical interventions during Covid-19 pandemic. medRxiv [Preprint]. 2022 [cited 2023 Mar 03]. Available from https://www.medrxiv.org/content/10.1101/2022.02.28.22271671v1

53~~49~~. Chernozhukov V, Kasahara H, Schrimpf P. Causal impact of masks, policies, behavior on early covid-19 pandemic in the U.S. Journal of Econometrics. 2021;220:23-62.

54~~50~~. Kovacs R, Dunaiski, M, Tukiainen J. Compulsory face mask policies do not affect community mobility in Germany. SSRN [Preprint]. 2020 [cited 2023 Mar 03]. Available from https://papers.ssrn.com/sol3/papers.cfm?abstract_id=3620070

55~~51~~. Shin SH, Ji H, Lim H. Heterogeneity in preventive behaviors during COVID-19: health risk, economic insecurity, and slanted information. Social Science & Medicine. 2021;278:113944.

56~~52~~. Zhao N, Yao S, Thomadsen R, Wang CB. The impact of social Distancing and masking on COVID-19 spread and consumer spending. SSRN [Preprint]. 2022 [cited 2023 Mar 03]. Available from https://papers.ssrn.com/sol3/papers.cfm?abstract_id=3725711

57~~3~~. Bansal P, Kessels R, Krueger R, Graham DJ. Preferences for using the London Underground during the COVID-19 pandemic. Transportation Research Part A. 2022;160:45-60.

5854. de Freitas AVC, Magalhães AM, Carricondo PC, Shinzato AA, Hida RY. Face masks can reduce the frequency of face touching: an observational crossover study. International Journal of Infectious Diseases. 2022;123:54-7.

5955. Chen YJ, Qin G, Chen J, Xu JL, Feng DY, Wu XY, et al. Comparison of face-touching behaviors before and during the coronavirus disease 2019 pandemic. JAMA Network Open. 2020;3(7):e2016924.

6056. Shiraly R, Shayan Z, McLaws ML. Face touching in the time of COVID-19 in Shiraz, Iran. American Journal of Infection Control. 2020;48:1559-61.

6157. Liebst LS, Ejbye-Ernst P, de Bruin M, Thomas J, Lindegaard MR. Face-touching behaviour as a possible correlate of mask-wearing: a video observational study of public place incidents during the COVID-19 pandemic. Transboundary and Emerging Diseases. 2022;69:1319-25.

6258. Kungurova Y, Mera R, Brewster E, Ali K, Fakoya AOJ. Covid-19 and face mask use: a St. Kitts case study. Open Access Macedonian Journal of Medical Sciences. 2020 Oct 26;8(T1):346-52.

6359. Tao ZY, Dong J, Culleton R. The use of facemasks may not lead to an increase in hand-face contact. Transboundary and Emerging Diseases. 2020;67:3038-40.

6460. van den Broek-Altenburg EM, Atherly AJ, Diehl SA, Gleason KM, Hart VC, MacLean CD, et al. Jobs, housing, and mask wearing: cross-sectional study of risk factors for COVID-19. JMIR Public Health and Surveillance. 2021;7(1):e24320.

6561. MacIntyre CR, Nguyen PY, Chughtai AA, Trent M, Gerber B, Steinhofel K, et al. Mask use, risk-mitigation behaviours and pandemic fatigue during the COVID-19 pandemic in five cities in Australia, the UK and USA: a cross-sectional survey. International Journal of Infectious Diseases. 2021;106:199-207.

662. Guenther B, Galizzi MM, Sanders JG. Heterogeneity in risk-taking during the COVID-19 pandemic: evidence from the UK lockdown. Frontiers in Psychology. 2021 Apr 01;12:643653.

67. Charness G, Gneezy U, Kuhn MA. Experimental methods: between-subject and within-subject design. Journal of Economic Behaviour & Organization. 2012 Jan 01; 81(1):1-8.

68. Samek A. Advantages and disadvantages of field experiments. In: Schram A, Ule A. Handbook of research methods and applications in experimental economics. Cheltenham (UK) & Northampton (USA). Edward Elgar Publishing: 2019. p. 104-120.

69. Lu CY. Observational studies: a review of study designs, challenges and strategies to reduce confounding. The International Journal of Clinical Practice. 2009 May; 63(5):691-697.

70. Mann CJ. Observational research methods. Research design II: cohort, cross sectional, and case-control studies. Emergency Medicine Journal. 2003; 20:54-60.

7. Supporting information

S1 Appendix. Search strategies

S2 Appendix. Quality criteria checklist

S3 Appendix. Data extraction tables

S4 Appendix. PRISMA 2020 checklist

S5 Appendix. PRISMA 2020 abstract checklist

S6 Appendix. Protocol

Responses to Reviewers

Re: Manuscript No: PONE-D-23-08549

Title: Effect of face-covering use on adherence to other COVID-19 protective behaviours: a systematic review

Dear Editor,

Thank you for your consideration of our paper. We would like to thank your reviewers for their constructive comments on our manuscript. We have made the revisions requested and hope that it is now acceptable for publication.

Point by point responses to the reviewer comments are given below. Changes made to existing text in the manuscript are shown with tracked changes in the file 'Manuscript with Track Changes' as requested.

Reviewer 1:

There are many relevant studies that I believe should be included in the background section to justify the need to study risk compensation behavior in this context:
Include the theory that predicts this risk compensation behavior (and references). I think references to Risk Compensation Theory and the Peltzman Effects should be included.
Include studies focusing on risk compensation related to other covid policies. These studies can provide more of framework for why risk compensation is believed to exist for face masking [provides list of studies].

Response:

We agree that this section would benefit from the additions suggested and thank the reviewer for providing links to relevant studies. We have added background theory on risk compensation and the Peltzman effect to the second paragraph to introduction, as well as information on studies focusing on risk compensation related to other COVID-19 protective behaviours to the third paragraph and have included nearly all of the studies suggested by the reviewer.

There are key details of the studies reported on that I believe should be included. For instance, it is unclear whether face masking is required in most the studies or not. For people who self-select into masking, they may generally adhere more to covid-safety measures than those who do not select in to wearing a mask. So those wearing masks may stay further away from those who do not choose to wear a mask. But it may be the case that those who self-select into wearing masks (even though they are adhere more to social distancing than those not opting into masks) may even choose a larger distance if they were not wearing a mask at all – suggesting risk compensation behavior. I think this type of element needs to at least be mentioned prior to the very end of the manuscript. It could be used to explain the results of many of these papers, and not reject risk compensation behavior.

Response:

We agree that the interaction between face covering use and face covering policy is an important area of consideration, and that it should be acknowledged that when people wear face coverings under voluntary conditions they are self-selecting into masking, and that this has potential implications for their own behaviour and the behaviour of those around them. We have added a column to the data extraction table (see S3_Appendix_with track changes) titled 'Real-world face covering policy at time of study' in which it is indicated for each study whether the policy was voluntary, mandatory, varied or not specified. We have also added a

subsection (sub-section 4.7) to the discussion section where we (i) summarise three studies which, in a natural experiment, investigated the interaction between face covering policy and face covering use and (ii) summarise, for each protective behaviour, a cross-sectional analysis investigating study result (e.g., positive or negative effect of face covering use on adherence) by real-world policy. We summarise by saying that there are no clear patterns with regards to this interaction and that we recommend further research.

Be consistent with writing out numbers. Either follow the rule that <10 is written out, or once one number is written out, then all other numbers are written out in that sentence.

Response:

We thank the reviewer for highlighting this inconsistency and it has now been addressed throughout the manuscript.

Line 26: The beginning of the sentence is written twice

Response:

We thank the reviewer for highlighting this error and it has now been corrected.

Line 31: The first part of the sentence is redundant to the previous paragraph

Lines 36-48: This should be reduced to one paragraph with more focus on the theory of risk compensation and the Peltzman effect. Further, these paragraphs are very repetitive and could benefit from tightening of the language.

Response:

We agree with both above points and the introduction has now been heavily edited, both to include literature on risk compensation and the Peltzman effect and to tighten the language and reduce repetition.

Lines 51-52: I suggest deleting the statement "assess available evidence relating to the effect of face covering use on adherence to other COVID-19 protective behaviours" since it does not add any new information

Response:

We agree and have deleted this statement.

Lines 52-54: But, this review does not make suggestions on "if and when it is appropriate to recommend or mandate face covering use during future infectious disease outbreaks." So, I suggest removing this statement.

Response:

We agree and have deleted this statement.

Lines 59-64: Since the protocol (and its amendments) are accessible at the available link, I don't think these statements are needed in the main text.

Response:

We agree and have deleted this statement.

Figure 1: This figure, although informative, does not include the 'stages' that are indicated in the text. Further, it does not explain what the stage of 'title and abstract screening' means. Does this mean that only the title and abstract were initially reviewed to determine if a paper was to be included in this review? If so, this needs more explanation.

Response:

We agree and have addressed this issue as follows: An explanation of title and abstract screening has been added to the paper, as has an explanation of full-text screening. The words title and abstract screening (stages one and two) and full-text screening have also been added to the figure in the relevant place to better link the text to the figure.

Line 96: Extra period.

Response:

We thank the reviewer for highlighting this error and it has now been corrected.

Lines 103-107: Is this information needed in the main text? I don't think so.

Response:

We have retained this information to satisfy items 5, 10a, 10b and 13a of the PRISMA checklist which are as follows:

"Specify the inclusion and exclusion criteria for the review and how studies were grouped for the syntheses"

"List and define all outcomes for which data were sought. Specify whether all results that were compatible with each outcome domain in each study were sought (e.g. for all measures, time points, analyses), and if not, the methods used to decide which results to collect."

"List and define all other variables for which data were sought (e.g. participant and intervention characteristics, funding sources). Describe any assumptions made about any missing or unclear information."

"Describe the processes used to decide which studies were eligible for each synthesis (e.g. tabulating the study intervention characteristics and comparing against the planned groups for each synthesis (item #5))."

Lines 111-127: The quality assessment that is conducted seems out of place since it is not used at all in the review analysis. Why not use the differing levels of quality from this assessment to conduct an additional analysis in the review? For example, do all low-quality papers find similar results, which are different than high-quality papers?

Response:

We agree that results of the quality assessment should be used in the review analysis. In line with the reviewer's suggestions, we have explored the outcomes of studies cross-sectionally by study quality, the full findings of which can be seen in Appendix 1 of this response letter. There is a suggestion of a relationship between study quality and study outcome in the case of face-touching, but no other clear patterns have emerged. These findings are now described in the new sub-section 3.8, and also mentioned in the face-touching subsection of the discussion, sub-section 4.4.

Lines 124-127: I don't know if this is needed in the main text. I suggest moving it to an appendix.

Response:

We have decided to retain these statements in order to satisfy PRISMA checklist item 11 which is as follows:

"Specify the methods used to assess risk of bias in the included studies, including details of the tool(s) used, how many reviewers assessed each study and whether they worked independently, and if applicable, details of automation tools used in the process."

Line 138: This sentence is unclear.

Response:

We agree. In line with this, and also with additional comments from reviewer 2 on the statements in this paragraph, the whole paragraph has been deleted, and an additional sentence added to previous paragraph instead.

Lines 152-153: It is unclear what this statement means: "face covering wearing on behalf of oneself and another person with whom one is interacting or is in proximity …"

Response:

We agree. The statement was intended to refer to situations in which two people are interacting in proximity and both are wearing face coverings. The language has been edited to try and convey this more clearly.

Lines 164-171: I don't think is paragraph is needed and suggest deleting it.

Response:

This paragraph has been retained to satisfy PRISMA checklist item 16b which is as follows:

"Cite studies that might appear to meet the inclusion criteria, but which were excluded, and *explain why they were excluded"*

Lines 236-237: Compared to who? Is this result found in comparison to those who reported not wearing a mask?

Response:

Yes, this result was found in comparison to those who reported not always wearing a mask. This clarification has been added and we thank the reviewer for raising this ambiguity.

Discussion section: Many statements are made about the strengths and weakness of the stated approaches without use of references

Response:

Appropriate references have now been added to the Discussion section.

Lines 312-315: This statement relates to my comments about self-selecting into masking. I think this is one huge advantage of lab experiments that should be more clearly explained.

Response:

This section has now been removed as we have discussed the topic of self-selected face-covering wearing in more detail in the new sub-section 4.7. Note that we refer to this new sub-section within this paragraph.

Reviewer 2:

My major comment is the distinct lack of meta-analysis, which to me is a shame. The authors clearly point out trends in individual studies, along with conflicting data from different studies. A meta-analysis of these studies (seperated by type of study, and type of intervention for example) would really help crystallise the conclusions that the authors draw. For example, face coverings might reduce face-touching and increase mobility from a forest plot, even if individual studies conflict on

this. Trends towards significance might also be important here, along with presentation of a heterogeneity statistic.

Response:

We have attempted to follow the reviewer's recommendation by consulting with colleagues who have worked on meta-analyses previously. However, we have been presented with logistical obstacles and have now reached the pragmatic decision to attempt to publish the review in line with the registered scope which will provide an opportunity for other researchers to apply meta-analytic techniques to some of the literature that we have identified and synthesised in our paper.

Whilst it may be the case for mobility that there are enough papers which are sufficiently homogenous to allow for them to be meta-analysed, we believe this is not the case for all behaviours. For example, the reviewer rightly notes that there are four observational studies on physical distancing which have different outcomes, and which would benefit from a meta-analysis. It should be noted however that there are three important differences between these studies. Firstly, whilst three of them compared physical distancing between individuals who were and were not wearing face-coverings, the fourth compared physical distancing between people in areas which did or did not have mandatory face covering use. Secondly, there are differences with regards to the operationalisation of the outcome variable; whilst two of the studies define a breach of physical distancing as coming within 1.5 metres of another person, one study uses 2m as their criterion distance, and another use 6ft. Thirdly, whilst one of the studies was conducted under conditions of mandatory face-covering use, for two of the studies the policy (mandatory or voluntary) was varied throughout the course of the study, and for one study the policy was not specified; as is discussed in the paper, the relationship between face covering use and adherence to other behaviours is likely to be affected by whether face covering use in mandatory or voluntary. This is one example of where a group of studies is sufficiently heterogenous as to make the adoption of meta-analysing them questionable, and there are other examples. For instance, whilst six observational studies investigate the effect of face-covering use on face-touching, in some cases face-touching is operationalised as a continuous variable (number of touches to the face) and in others it is operationalised as a categorical variable (whether or not the face was touched at all during a set length of time; a length of time which is not consistent across studies). Additionally, across all studies there is significant heterogeneity with regards to the independent variable; in some cases the effect of face-covering use by the self is investigated, in others it is face-covering use by others, and in others the effect of a face mask mandate.

Abstract:
Minor comments
- I would suggest including some statistics (OR/p values) in your abstract, for the meta-analysed data on how face covering use might reduce face-touching and increase mobility even if the trend is not significant.

Response:

We refer to the initial response concerning a meta-analysis

Introduction
Minor comments
- 'prior to the introduction of the vaccination' is typed twice.

Response:

We thank the reviewer for highlighting this error and it has now been corrected.

Results
Major comments - I am surprised that no meta-analysis of effects was done. This to me makes the results very confusing. For example in section 3.1, there were four observational studies that found a positive or neutral relationship between face covering use or face covering policy and physical distancing; individually, three found no significant relationship between these two variables whilst a fourth one did. Surely this justifies a meta-analysis that takes into account the sample size and thus can present an overall effect?

Similarly for mobility - the 10 natural experiments had conflicting results. Why not meta-analyse these studies? Whilst I acknowledge significant heterogeneity, I do think it is still worth doing an overall analysis, which would help support statements that the authors make in their discussion.

Response:

We refer to the initial response concerning a meta-analysis

Minor comments - Sections 3.1: it is unusual to write in manuscripts: 'note that in the paragraph that follows..' - I think this could be covered in methods in a more 'reportive' way. 'We found that some papers included more than one methodology in their study and thus, the number of studies described for each methodology may exceed the total number of studies'.

Response:

We agree that the language in this statement is not appropriate. We have deleted it and replaced it with a sentence similar to that which the reviewer suggests and thank the reviewer for this suggestion.

I think the last paragraph of the first section is unnecessary.

Response:

We have retained this paragraph as it outlines the final rationale and aim of the study, but we have shortened it to be more concise.

Discussion
The authors make multiple clear statements; which were not present in their abstract and in my opinion, isn't necessarily supported by their results, given the lack of meta-analysis. For example - the authors mention in the first paragraph in 4.1 that the majority of lab experiments found that physical distancing was lower in conditions of face covering use, whereas the majority of field experiments found that physical distancing was greater or unchanged. This clearly deserves meta-analysis (seperately) in my opinion to support this conclusion.

Response:

We refer to the initial response concerning a meta-analysis. Additionally, heterogeneity amongst these studies should be acknowledged. Firstly, amongst the field studies there is heterogeneity with regards to the operationalisation of outcome variables; in some cases physical distancing is operationalised as a binary categorical variable which defines participants as adhering or not adhering to physical distancing guidelines (and across studies there is heterogeneity with regards to the distance by which adherence is defined), in others

it is operationalised as continuous variable, measuring the actual distance kept by participant between themselves and a confederate, and in others is operationalised as a continuous variable, measuring the number of times a participant violates physical distancing guidelines). There is also heterogeneity with regards to the independent variable across these studies; in some cases it is face covering use by the participant, and in some cases it is face covering use by others (e.g., confederates). There is also heterogeneity with regards to the real-world face covering policy during the time of study; in some cases face covering use is mandatory, in some it is voluntary, and in some it is varied.

Appendix 1: Analysis of study quality by effect or association

The tables and paragraphs below (grouped by protective behaviour) outline findings of cross-sectional analysing the studies' results by study quality. For the sake of simplicity, studies where there was a conditionally positive or negative effect or association (e.g., where the effect was conditional on gender) have been categorised as simply having a positive or negative effect or association (rather than a conditionally positive or negative effect or association).

Physical distancing

Table 1 – Physical distancing: study quality by effect or association

**Study quality**	**Effect or association**				
	Positive	Neutral	Negative	Equivocal	Total
High	1	0	0	0	**1**
Medium	9	6	11	2	**28**
Low	2	0	0	0	**2**
**Total**	**12**	**6**	**11**	**2**	**31**

Of the 31 studies on physical distancing, most (n = 28) studies were of a medium quality, and of these, nine showed a positive effect or association whilst 11 showed a negative effect or association. The remainder found that the effect or association was neutral (n = 6) or equivocal (n = 2). There were two studies of low quality, and one of high quality, and all of these found a positive effect or association. Therefore, analysing the results of studies on physical distancing cross-sectionally by study quality does not reveal any clear patterns and therefore does not add any substantive insight to the overall analysis.

Mobility

Table 2 – Mobility: study quality by effect or association

**Study quality**	**Effect or association**				
	Positive	Neutral	Negative	Equivocal	Total
High	4	3	1	0	**8**
Medium	3	1	1	0	**5**
Low	0	0	0	0	**0**
**Total**	**7**	**4**	**2**	**0**	**13**

Of the 13 studies on mobility, eight were of a high quality, five were of a medium quality, and none were of a low quality. Of the high-quality studies, only one found a negative effect or association, as was the case for medium quality studies, and the most frequent result for both high and medium quality studies was a positive effect or association. Therefore, analysing the results of studies on mobility cross-sectionally by study quality does not reveal any clear patterns and therefore does not add any substantive insight to the overall analysis.

Face-touching

Table 3 – Face-touching: study quality by effect or association

**Study quality**	**Effect or association**				
	Positive	Neutral	Negative	Equivocal	Total
High	0	0	0	0	**0**
Medium	0	0	4	0	**4**
Low	1	1	1	0	**3**
**Total**	**1**	**1**	**5**	**0**	**7**

Of the seven studies on face-touching, four were of a medium quality and one was of a low quality. All the medium quality studies found a negative effect or association whereas the three low quality papers were evenly split with regards to the results found (one positive, one neutral and one negative effect or association). Therefore, the general finding that most studies on face-touching found negative effects or associations is corroborated when analysing the studies cross-sectionally by study quality, as all of the studies which were of a relative higher quality found this same result; that is, that face-covering use has negative effect on (or is negatively associated with) face-touching.

Hand hygiene

Table 4 – Hand hygiene: study quality by effect or association

**Study quality**	**Effect or association**				
	Positive	Neutral	Negative	Equivocal	Total
High	0	0	0	0	**0**
Medium	3	1	0	0	**4**
Low	0	0	0	0	**0**
**Total**	**3**	**1**	**0**	**0**	**4**

All the studies on hand hygiene (n = 4) were of a medium quality and so it is not possible to cross-sectionally analyse them by study quality.

Close contacts

Table 5 – Close contacts: study quality by effect or association

**Study quality**	**Effect or association**				
	Positive	Neutral	Negative	Equivocal	Total
High	0	1	0	0	**1**
Medium	1	0	2	1	**4**
Low	0	0	0	0	**0**
**Total**	**1**	**1**	**2**	**1**	**5**

Of the five studies on close contacts, most (n = 4) were of a medium quality, and one was of a high quality. Of the medium quality studies two found a negative effect or association, one found a positive association, and one found that the association was equivocal. The high-quality study found a neutral (i.e., nonsignificant) effect. Therefore, analysing the results of studies on close contacts cross-

sectionally by study quality does not reveal any clear patterns and therefore does not add any substantive insight to the overall analysis.

Generalised protective behaviour

Table 6 – Generalised protective behaviour: study quality by effect or association

**Study quality**	**Effect or association**				
	Positive	Neutral	Negative	Equivocal	Total
High	0	0	0	0	**0**
Medium	2	0	0	0	**2**
Low	0	0	0	0	**0**
**Total**	**2**	0	0	0	**2**

Both studies on generalised protective behaviour were of a medium quality and so it is not possible to cross-sectionally analyse them by study quality.
