# Supplementary material for: Effect of face-covering use on adherence to other COVID-19 protective behaviours: A systematic review
Source: PLoS One. 2024 Apr 11;19(4):e0284629. doi: 10.1371/journal.pone.0284629 (PMC11008824; doi:10.1371/journal.pone.0284629)
Supplement: Attachment — Submitted filename: Response to Reviewers.docx [file pone.0284629.s007.docx]

**Responses to Reviewers**

**Re: Manuscript No: PONE-D-23-08549**

Title: Effect of face-covering use on adherence to other COVID-19 protective behaviours: a systematic review

Dear Editor,

Thank you for your consideration of our paper. We would like to thank your reviewers for their constructive comments on our manuscript. We have made the revisions requested and hope that it is now acceptable for publication.

**Point by point responses to the reviewer comments are given below. Changes made to existing text in the manuscript are shown with tracked changes in the file ‘Manuscript with Track Changes’ as requested.**

**Reviewer 1:**

*There are many relevant studies that I believe should be included in the background section to justify the need to study risk compensation behavior in this context:
Include the theory that predicts this risk compensation behavior (and references). I think references to Risk Compensation Theory and the Peltzman Effects should be included.
Include studies focusing on risk compensation related to other covid policies. These studies can provide more of framework for why risk compensation is believed to exist for face masking* [provides list of studies]*.*

Response:

We agree that this section would benefit from the additions suggested and thank the reviewer for providing links to relevant studies. We have added background theory on risk compensation and the Peltzman effect to the second paragraph to introduction, as well as information on studies focusing on risk compensation related to other COVID-19 protective behaviours to the third paragraph and have included nearly all of the studies suggested by the reviewer.

*There are key details of the studies reported on that I believe should be included. For instance, it is unclear whether face masking is required in most the studies or not. For people who self-select into masking, they may generally adhere more to covid-safety measures than those who do not select in to wearing a mask. So those wearing masks may stay further away from those who do not choose to wear a mask. But it may be the case that those who self-select into wearing masks (even though they are adhere more to social distancing than those not opting into masks) may even choose a larger distance if they were not wearing a mask at all – suggesting risk compensation behavior. I think this type of element needs to at least be mentioned prior to the very end of the manuscript. It could be used to explain the results of many of these papers, and not reject risk compensation behavior.*

Response:

We agree that the interaction between face covering use and face covering policy is an important area of consideration, and that it should be acknowledged that when people wear face coverings under voluntary conditions they are self-selecting into masking, and that this has potential implications for their own behaviour and the behaviour of those around them. We have added a column to the data extraction table (see S3_Appendix_with track changes) titled ‘Real-world face covering policy at time of study’ in which it is indicated for each study whether the policy was voluntary, mandatory, varied or not specified. We have also added a subsection (sub-section 4.7) to the discussion section where we (i) summarise three studies which, in a natural experiment, investigated the interaction between face covering policy and face covering use and (ii) summarise, for each protective behaviour, a cross-sectional analysis investigating study result (e.g., positive or negative effect of face covering use on adherence) by real-world policy. We summarise by saying that there are no clear patterns with regards to this interaction and that we recommend further research.

*Be consistent with writing out numbers. Either follow the rule that <10 is written out, or once one number is written out, then all other numbers are written out in that sentence.*

Response:

We thank the reviewer for highlighting this inconsistency and it has now been addressed throughout the manuscript.

*Line 26: The beginning of the sentence is written twice*

Response:

We thank the reviewer for highlighting this error and it has now been corrected.

*Line 31: The first part of the sentence is redundant to the previous paragraph*

*Lines 36-48: This should be reduced to one paragraph with more focus on the theory of risk compensation and the Peltzman effect. Further, these paragraphs are very repetitive and could benefit from tightening of the language.*

Response:

We agree with both above points and the introduction has now been heavily edited, both to include literature on risk compensation and the Peltzman effect and to tighten the language and reduce repetition.

*Lines 51-52: I suggest deleting the statement “assess available evidence relating to the effect of face covering use on adherence to other COVID-19 protective behaviours” since it does not add any new information*

Response:

We agree and have deleted this statement.

*Lines 52-54: But, this review does not make suggestions on “if and when it is appropriate to recommend or mandate face covering use during future infectious disease outbreaks.” So, I suggest removing this statement.*

Response:

We agree and have deleted this statement.

*Lines 59-64: Since the protocol (and its amendments) are accessible at the available link, I don’t think these statements are needed in the main text.*

Response:

We agree and have deleted this statement.

*Figure 1: This figure, although informative, does not include the ‘stages’ that are indicated in the text. Further, it does not explain what the stage of ‘title and abstract screening’ means. Does this mean that only the title and abstract were initially reviewed to determine if a paper was to be included in this review? If so, this needs more explanation.*

Response:

We agree and have addressed this issue as follows: An explanation of title and abstract screening has been added to the paper, as has an explanation of full-text screening. The words title and abstract screening (stages one and two) and full-text screening have also been added to the figure in the relevant place to better link the text to the figure.

*Line 96: Extra period.*

Response:

We thank the reviewer for highlighting this error and it has now been corrected.

*Lines 103-107: Is this information needed in the main text? I don’t think so.*

Response:

We have retained this information to satisfy items 5, 10a, 10b and 13a of the PRISMA checklist which are as follows:

“Specify the inclusion and exclusion criteria for the review and how studies were grouped for the syntheses”

“List and define all outcomes for which data were sought. Specify whether all results that were compatible with each outcome domain in each study were sought (e.g. for all measures, time points, analyses), and if not, the methods used to decide which results to collect.”

“List and define all other variables for which data were sought (e.g. participant and intervention characteristics, funding sources). Describe any assumptions made about any missing or unclear information.”

“Describe the processes used to decide which studies were eligible for each synthesis (e.g. tabulating the study intervention characteristics and comparing against the planned groups for each synthesis (item #5)).”

*Lines 111-127: The quality assessment that is conducted seems out of place since it is not used at all in the review analysis. Why not use the differing levels of quality from this assessment to conduct an additional analysis in the review? For example, do all low-quality papers find similar results, which are different than high-quality papers?*

Response:

We agree that results of the quality assessment should be used in the review analysis. In line with the reviewer’s suggestions, we have explored the outcomes of studies cross-sectionally by study quality, the full findings of which can be seen in Appendix 1 of this response letter. There is a suggestion of a relationship between study quality and study outcome in the case of face-touching, but no other clear patterns have emerged. These findings are now described in the new sub-section 3.8, and also mentioned in the face-touching subsection of the discussion, sub-section 4.4.

*Lines 124-127: I don’t know if this is needed in the main text. I suggest moving it to an appendix.*

Response:

We have decided to retain these statements in order to satisfy PRISMA checklist item 11 which is as follows:

“Specify the methods used to assess risk of bias in the included studies, including details of the tool(s) used, how many reviewers assessed each study and whether they worked independently, and if applicable, details of automation tools used in the process.”

*Line 138: This sentence is unclear.*

Response:

We agree. In line with this, and also with additional comments from reviewer 2 on the statements in this paragraph, the whole paragraph has been deleted, and an additional sentence added to previous paragraph instead.

*Lines 152-153: It is unclear what this statement means: “face covering wearing on behalf of oneself and another person with whom one is interacting or is in proximity …”*

Response:

We agree. The statement was intended to refer to situations in which two people are interacting in proximity and both are wearing face coverings. The language has been edited to try and convey this more clearly.

*Lines 164-171: I don’t think is paragraph is needed and suggest deleting it.*

Response:

This paragraph has been retained to satisfy PRISMA checklist item 16b which is as follows:

“Cite studies that might appear to meet the inclusion criteria, but which were excluded, and *explain why they were excluded”*

*Lines 236-237: Compared to who? Is this result found in comparison to those who reported not wearing a mask?*

Response:

Yes, this result was found in comparison to those who reported not always wearing a mask. This clarification has been added and we thank the reviewer for raising this ambiguity.

*Discussion section: Many statements are made about the strengths and weakness of the stated approaches without use of references*

Response:

Appropriate references have now been added to the Discussion section.

*Lines 312-315: This statement relates to my comments about self-selecting into masking. I think this is one huge advantage of lab experiments that should be more clearly explained.*

Response:

This section has now been removed as we have discussed the topic of self-selected face-covering wearing in more detail in the new sub-section 4.7. Note that we refer to this new sub-section within this paragraph.

**Reviewer 2:**

*My major comment is the distinct lack of meta-analysis, which to me is a shame. The authors clearly point out trends in individual studies, along with conflicting data from different studies. A meta-analysis of these studies (seperated by type of study, and type of intervention for example) would really help crystallise the conclusions that the authors draw. For example, face coverings might reduce face-touching and increase mobility from a forest plot, even if individual studies conflict on this. Trends towards significance might also be important here, along with presentation of a heterogeneity statistic.*

Response:

We have attempted to follow the reviewer’s recommendation by consulting with colleagues who have worked on meta-analyses previously. However, we have been presented with logistical obstacles and have now reached the pragmatic decision to attempt to publish the review in line with the registered scope which will provide an opportunity for other researchers to apply meta-analytic techniques to some of the literature that we have identified and synthesised in our paper.

Whilst it may be the case for mobility that there are enough papers which are sufficiently homogenous to allow for them to be meta-analysed, we believe this is not the case for all behaviours. For example, the reviewer rightly notes that there are four observational studies on physical distancing which have different outcomes, and which would benefit from a meta-analysis. It should be noted however that there are three important differences between these studies. Firstly, whilst three of them compared physical distancing between individuals who were and were not wearing face-coverings, the fourth compared physical distancing between people in areas which did or did not have mandatory face covering use. Secondly, there are differences with regards to the operationalisation of the outcome variable; whilst two of the studies define a breach of physical distancing as coming within 1.5 metres of another person, one study uses 2m as their criterion distance, and another use 6ft. Thirdly, whilst one of the studies was conducted under conditions of mandatory face-covering use, for two of the studies the policy (mandatory or voluntary) was varied throughout the course of the study, and for one study the policy was not specified; as is discussed in the paper, the relationship between face covering use and adherence to other behaviours is likely to be affected by whether face covering use in mandatory or voluntary. This is one example of where a group of studies is sufficiently heterogenous as to make the adoption of meta-analysing them questionable, and there are other examples. For instance, whilst six observational studies investigate the effect of face-covering use on face-touching, in some cases face-touching is operationalised as a continuous variable (number of touches to the face) and in others it is operationalised as a categorical variable (whether or not the face was touched at all during a set length of time; a length of time which is not consistent across studies). Additionally, across all studies there is significant heterogeneity with regards to the independent variable; in some cases the effect of face-covering use by the self is investigated, in others it is face-covering use by others, and in others the effect of a face mask mandate.

*Abstract:
Minor comments
- I would suggest including some statistics (OR/p values) in your abstract, for the meta-analysed data on how face covering use might reduce face-touching and increase mobility even if the trend is not significant.*

Response:

We refer to the initial response concerning a meta-analysis

*Introduction
Minor comments
- 'prior to the introduction of the vaccination' is typed twice.*

Response:

We thank the reviewer for highlighting this error and it has now been corrected.

*Results
Major comments - I am surprised that no meta-analysis of effects was done. This to me makes the results very confusing. For example in section 3.1, there were four observational studies that found a positive or neutral relationship between face covering use or face covering policy and physical distancing; individually, three found no significant relationship between these two variables whilst a fourth one did. Surely this justifies a meta-analysis that takes into account the sample size and thus can present an overall effect?

Similarly for mobility - the 10 natural experiments had conflicting results. Why not meta-analyse these studies? Whilst I acknowledge significant heterogeneity, I do think it is still worth doing an overall analysis, which would help support statements that the authors make in their discussion.*

Response:

We refer to the initial response concerning a meta-analysis

*Minor comments - Sections 3.1: it is unusual to write in manuscripts: 'note that in the paragraph that follows..' - I think this could be covered in methods in a more 'reportive' way. 'We found that some papers included more than one methodology in their study and thus, the number of studies described for each methodology may exceed the total number of studies'.*

Response:

We agree that the language in this statement is not appropriate. We have deleted it and replaced it with a sentence similar to that which the reviewer suggests and thank the reviewer for this suggestion.

*I think the last paragraph of the first section is unnecessary.*

Response:

We have retained this paragraph as it outlines the final rationale and aim of the study, but we have shortened it to be more concise.

*Discussion
The authors make multiple clear statements; which were not present in their abstract and in my opinion, isn't necessarily supported by their results, given the lack of meta-analysis. For example - the authors mention in the first paragraph in 4.1 that the majority of lab experiments found that physical distancing was lower in conditions of face covering use, whereas the majority of field experiments found that physical distancing was greater or unchanged. This clearly deserves meta-analysis (seperately) in my opinion to support this conclusion.*

Response:

We refer to the initial response concerning a meta-analysis. Additionally, heterogeneity amongst these studies should be acknowledged. Firstly, amongst the field studies there is heterogeneity with regards to the operationalisation of outcome variables; in some cases physical distancing is operationalised as a binary categorical variable which defines participants as adhering or not adhering to physical distancing guidelines (and across studies there is heterogeneity with regards to the distance by which adherence is defined), in others it is operationalised as continuous variable, measuring the actual distance kept by participant between themselves and a confederate, and in others is operationalised as a continuous variable, measuring the number of times a participant violates physical distancing guidelines). There is also heterogeneity with regards to the independent variable across these studies; in some cases it is face covering use by the participant, and in some cases it is face covering use by others (e.g., confederates). There is also heterogeneity with regards to the real-world face covering policy during the time of study; in some cases face covering use is mandatory, in some it is voluntary, and in some it is varied.

**Appendix 1: Analysis of study quality by effect or association**

The tables and paragraphs below (grouped by protective behaviour) outline findings of cross-sectional analysing the studies’ results by study quality. For the sake of simplicity, studies where there was a conditionally positive or negative effect or association (e.g., where the effect was conditional on gender) have been categorised as simply having a positive or negative effect or association (rather than a conditionally positive or negative effect or association).

**Physical distancing**

**Table 1 – Physical distancing: study quality by effect or association**

| **Study quality** | **Effect or association** | | | | |
| --- | --- | --- | --- | --- | --- |
|  | Positive | Neutral | Negative | Equivocal | Total |
| High | 1 | 0 | 0 | 0 | **1** |
| Medium | 9 | 6 | 11 | 2 | **28** |
| Low | 2 | 0 | 0 | 0 | **2** |
| **Total** | **12** | **6** | **11** | **2** | **31** |

Of the 31 studies on physical distancing, most (n = 28) studies were of a medium quality, and of these, nine showed a positive effect or association whilst 11 showed a negative effect or association. The remainder found that the effect or association was neutral (n = 6) or equivocal (n = 2). There were two studies of low quality, and one of high quality, and all of these found a positive effect or association. Therefore, analysing the results of studies on physical distancing cross-sectionally by study quality does not reveal any clear patterns and therefore does not add any substantive insight to the overall analysis.

**Mobility**

**Table 2 – Mobility: study quality by effect or association**

| **Study quality** | **Effect or association** | | | | |
| --- | --- | --- | --- | --- | --- |
|  | Positive | Neutral | Negative | Equivocal | Total |
| High | 4 | 3 | 1 | 0 | **8** |
| Medium | 3 | 1 | 1 | 0 | **5** |
| Low | 0 | 0 | 0 | 0 | **0** |
| **Total** | **7** | **4** | **2** | **0** | **13** |

Of the 13 studies on mobility, eight were of a high quality, five were of a medium quality, and none were of a low quality. Of the high-quality studies, only one found a negative effect or association, as was the case for medium quality studies, and the most frequent result for both high and medium quality studies was a positive effect or association. Therefore, analysing the results of studies on mobility cross-sectionally by study quality does not reveal any clear patterns and therefore does not add any substantive insight to the overall analysis.

**Face-touching**

**Table 3 – Face-touching: study quality by effect or association**

| **Study quality** | **Effect or association** | | | | |
| --- | --- | --- | --- | --- | --- |
|  | Positive | Neutral | Negative | Equivocal | Total |
| High | 0 | 0 | 0 | 0 | **0** |
| Medium | 0 | 0 | 4 | 0 | **4** |
| Low | 1 | 1 | 1 | 0 | **3** |
| **Total** | **1** | **1** | **5** | **0** | **7** |

Of the seven studies on face-touching, four were of a medium quality and one was of a low quality. All the medium quality studies found a negative effect or association whereas the three low quality papers were evenly split with regards to the results found (one positive, one neutral and one negative effect or association). Therefore, the general finding that most studies on face-touching found negative effects or associations is corroborated when analysing the studies cross-sectionally by study quality, as all of the studies which were of a relative higher quality found this same result; that is, that face-covering use has negative effect on (or is negatively associated with) face-touching.

**Hand hygiene**

**Table 4 – Hand hygiene: study quality by effect or association**

| **Study quality** | **Effect or association** | | | | |
| --- | --- | --- | --- | --- | --- |
|  | Positive | Neutral | Negative | Equivocal | Total |
| High | 0 | 0 | 0 | 0 | **0** |
| Medium | 3 | 1 | 0 | 0 | **4** |
| Low | 0 | 0 | 0 | 0 | **0** |
| **Total** | **3** | **1** | **0** | **0** | **4** |

All the studies on hand hygiene (n = 4) were of a medium quality and so it is not possible to cross-sectionally analyse them by study quality.

**Close contacts**

**Table 5 – Close contacts: study quality by effect or association**

| **Study quality** | **Effect or association** | | | | |
| --- | --- | --- | --- | --- | --- |
|  | Positive | Neutral | Negative | Equivocal | Total |
| High | 0 | 1 | 0 | 0 | **1** |
| Medium | 1 | 0 | 2 | 1 | **4** |
| Low | 0 | 0 | 0 | 0 | **0** |
| **Total** | **1** | **1** | **2** | **1** | **5** |

Of the five studies on close contacts, most (n = 4) were of a medium quality, and one was of a high quality. Of the medium quality studies two found a negative effect or association, one found a positive association, and one found that the association was equivocal. The high-quality study found a neutral (i.e., nonsignificant) effect. Therefore, analysing the results of studies on close contacts cross-sectionally by study quality does not reveal any clear patterns and therefore does not add any substantive insight to the overall analysis.

**Generalised protective behaviour**

**Table 6 – Generalised protective behaviour: study quality by effect or association**

| **Study quality** | **Effect or association** | | | | |
| --- | --- | --- | --- | --- | --- |
|  | Positive | Neutral | Negative | Equivocal | Total |
| High | 0 | 0 | 0 | 0 | **0** |
| Medium | 2 | 0 | 0 | 0 | **2** |
| Low | 0 | 0 | 0 | 0 | **0** |
| **Total** | **2** | 0 | 0 | 0 | **2** |

Both studies on generalised protective behaviour were of a medium quality and so it is not possible to cross-sectionally analyse them by study quality.
