# Supplementary material for: Effect of face-covering use on adherence to other COVID-19 protective behaviours: A systematic review
Source: PLoS One. 2024 Apr 11;19(4):e0284629. doi: 10.1371/journal.pone.0284629 (PMC11008824; doi:10.1371/journal.pone.0284629)
Supplement: Attachment — Submitted filename: Response to reviewers 13.03.24.docx [file pone.0284629.s009.docx]

Reviewers' comments:

Reviewer's Responses to Questions

**Comments to the Author**

1. If the authors have adequately addressed your comments raised in a previous round of review and you feel that this manuscript is now acceptable for publication, you may indicate that here to bypass the “Comments to the Author” section, enter your conflict of interest statement in the “Confidential to Editor” section, and submit your "Accept" recommendation.

Reviewer #1: (No Response)

Reviewer #2: (No Response)

Reviewer #3: All comments have been addressed

2. Is the manuscript technically sound, and do the data support the conclusions?

The manuscript must describe a technically sound piece of scientific research with data that supports the conclusions. Experiments must have been conducted rigorously, with appropriate controls, replication, and sample sizes. The conclusions must be drawn appropriately based on the data presented.

Reviewer #1: Yes

Reviewer #2: Partly

Reviewer #3: Yes

3. Has the statistical analysis been performed appropriately and rigorously?

Reviewer #1: N/A

Reviewer #2: No

Reviewer #3: N/A

4. Have the authors made all data underlying the findings in their manuscript fully available?

The [PLOS Data policy](https://eur01.safelinks.protection.outlook.com/?url=http%3A%2F%2Fwww.plosone.org%2Fstatic%2Fpolicies.action%23sharing&data=05%7C02%7CHolly.Carter%40ukhsa.gov.uk%7C9febfee3ee3b41091a0608dc27bd474b%7Cee4e14994a354b2ead475f3cf9de8666%7C0%7C0%7C638428941130843133%7CUnknown%7CTWFpbGZsb3d8eyJWIjoiMC4wLjAwMDAiLCJQIjoiV2luMzIiLCJBTiI6Ik1haWwiLCJXVCI6Mn0%3D%7C0%7C%7C%7C&sdata=I3s3dXlYlp1B3j9YVyIpgjJa5tgNBkJ%2FxK5e8%2FWvJnA%3D&reserved=0) requires authors to make all data underlying the findings described in their manuscript fully available without restriction, with rare exception (please refer to the Data Availability Statement in the manuscript PDF file). The data should be provided as part of the manuscript or its supporting information, or deposited to a public repository. For example, in addition to summary statistics, the data points behind means, medians and variance measures should be available. If there are restrictions on publicly sharing data—e.g. participant privacy or use of data from a third party—those must be specified.

Reviewer #1: Yes

Reviewer #2: Yes

Reviewer #3: Yes

5. Is the manuscript presented in an intelligible fashion and written in standard English?

PLOS ONE does not copyedit accepted manuscripts, so the language in submitted articles must be clear, correct, and unambiguous. Any typographical or grammatical errors should be corrected at revision, so please note any specific errors here.

Reviewer #1: No

Reviewer #2: Yes

Reviewer #3: Yes

6. Review Comments to the Author

Please use the space provided to explain your answers to the questions above. You may also include additional comments for the author, including concerns about dual publication, research ethics, or publication ethics. (Please upload your review as an attachment if it exceeds 20,000 characters)

Reviewer #1: I enjoyed reading the revised manuscript, “Effect of face-covering use on adherence to other COVID-19 protective behaviours: a systematic review”. The potential for adverse behavior related to risk compensation from COVID-related protective measures is a very important issue. I believe the changes made improved the quality of the manuscript and highlight the potential issues related to face making. Yet, there are many grammatical and clarification issues I believe should be addressed. I have highlighted my specific comments in the attached manuscript through embedded comments.

*We thank the reviewer for their detailed review and helpful suggestions for improving grammar and clarity. We have incorporated all suggested changes and feel that the manuscript is strengthened as a result.*

Reviewer #2: Thank you for the opportunity to re-review this manuscript. The authors have put much work into this revision.

My main feedback with regards to this work was the lack of meta-analysis of the study effects. The authors, having consulted with colleagues who have performed meta-analyses in the past, have decided to not apply meta-analysis techniques. One of the reasons they provide is the high level of heterogeneity between studies.

It is usual for meta-analysis exploring the field of social science to have high degrees of heterogeneity - in my opinion, this does not preclude pooling of data and has been done in the past. Instead, one can explore heterogeneity through various sensitivity analyses, which provide an important visualisation of the data available. This would, without words, highlight much of the variation the authors discuss in their discussion section, and therefore the need to improve data collection and analyses, including greater standardisation across studies.

The main reason for why I suggested a meta-analyses is that I don't feel like the authors have made the most out of the data that they have synthesised. The authors conclude that the impact of face coverings on behaviour is inconsistent, with some finding and effect whilst others do not - this is clearly going to be impacted by the size of the study and may actually be the incorrect conclusion. Furthermore, the author says that 'there is no clear consensus in the literature as to whether face covering use improves adherence to other protective behaviours...' The authors must at the very least, if they not meta-analysing, discuss more precisely the contexts in which face coverings may change behaviour, and in other circumstances they do not. This again is the point of a data synthesis; if there is already a clear consensus in the literature, why do a data synthesis in the first place?

*We thank the reviewer for taking the time to review our manuscript, and for their considered comments. As previously discussed, and noted by the reviewer, we have decided not to carry out a meta-analysis due to the heterogeneity of the studies included within the review. The Cochrane handbook cautions against the use of meta-analysis when studies have high levels of heterogeneity, due to the increased risk of errors or incorrect conclusions (Cochrane Handbook, Chapter 10: Analysing data and undertaking meta-analyses). In line with this, and in response to one of Reviewer 1’s comments, we have now added the following text to the limitations in section 4.8 to explain our decision not to carry out meta-analysis: “An additional consideration relates to the potential to conduct a meta-analysis of the included studies. Given the heterogeneity of the included studies (both in terms of clinical heterogeneity and methodological heterogeneity), a decision was taken not to carry out a meta-analysis. This is in line with Cochrane guidance on heterogeneity within meta-analysis, which states that meta-analysis should only be undertaken if studies are considered to be sufficiently similar to ensure a meaningful outcome and avoid drawing potentially misleading conclusions. However, as further research is conducted in this area, potentially resulting in more clinically and methodologically similar studies, future studies could build on this review by carrying out a meta-analysis”.*

*While we have decided not to carry out a meta-analysis, for the reasons outlined above, we agree with Reviewer 2 that a more precise overall discussion of the contexts in which face coverings do, or do not, change behaviour would be beneficial to the reader, and would make the review more impactful. We had previously discussed the overall impact of face covering use in different contexts within each behavioural outcome section of the discussion (sections 4.1 – 4.6). We have now also expanded the overall conclusion to state more precisely the contexts in which face coverings may impact different behaviours, and to highlight for which behaviours the evidence-base allows potential conclusions about the impact of face coverings to be reached, and for which behaviours further research is required:*

*“For some behavioural outcomes, it is possible to draw tentative conclusions regarding the impact of face coverings on behaviour. For example, evidence suggests that wearing a face covering reduces the amount that an individual touches their face; face coverings may therefore have a positive impact on face touching behaviours. Conversely, evidence suggests that the introduction of face mask mandates may increase mobility; face coverings may therefore have a negative impact on mobility-related behaviours. Some studies suggest that there may be a relationship between wearing a face covering and other protective behaviours (such as increased hand hygiene). However, these studies are predominately cross-sectional and rely on self-report measures; they therefore do not allow conclusions to be drawn about the impact of face covering use on adherence to these behaviours. Evidence relating to the impact of face covering use on physical distancing and close contacts is mixed. Findings are particularly inconsistent in relation to physical distancing, with considerable discrepancy found between study types. Whilst assessments of behaviour in real-life settings tend to find that face covering use either increases or has no impact upon physical distancing, lab experiments tend to suggest that physical distancing decreases in conditions of face covering use. There is some suggestion that the impact of face covering use on physical distancing could be affected by whether or not mandatory face covering policies are in effect (which would indicate that conclusions drawn from a voluntary context should not be directly applied to a mandatory context and vice versa) but further assessment of this potential moderating effect is warranted. Evidence relating to the potential impact of risk compensation is also inconsistent; while some studies suggest risk compensation may play a role in shaping behaviour, others suggest it has no impact.* *Overall, this review highlights that the impact of face covering use on other protective behaviours is likely to vary based on the behaviour under investigation. While for some behaviours the evidence suggests a positive impact (e.g., face coverings may result in reduced face touching), for other behaviours the evidence suggests a negative impact (e.g., increased mobility), or provides no clear consensus (e.g., physical distancing, close contacts, and adherence to other protective behaviours).”*

Reviewer #3: This is a thorough and balanced systematic review regarding the effect of face-covering use on adherence to other COVID-19 protective behaviours, and is of value in informing response to future responses to similar public health threats.

7. PLOS authors have the option to publish the peer review history of their article ([what does this mean?](https://eur01.safelinks.protection.outlook.com/?url=https%3A%2F%2Fjournals.plos.org%2Fplosone%2Fs%2Feditorial-and-peer-review-process%23loc-peer-review-history&data=05%7C02%7CHolly.Carter%40ukhsa.gov.uk%7C9febfee3ee3b41091a0608dc27bd474b%7Cee4e14994a354b2ead475f3cf9de8666%7C0%7C0%7C638428941130849006%7CUnknown%7CTWFpbGZsb3d8eyJWIjoiMC4wLjAwMDAiLCJQIjoiV2luMzIiLCJBTiI6Ik1haWwiLCJXVCI6Mn0%3D%7C0%7C%7C%7C&sdata=r9rNJ%2Bry1b%2BX9Wn93dM%2BEVeIJ9cTCovkAc9Asugk2F0%3D&reserved=0)). If published, this will include your full peer review and any attached files.


If you choose “no”, your identity will remain anonymous but your review may still be made public.


**Do you want your identity to be public for this peer review?** For information about this choice, including consent withdrawal, please see our [Privacy Policy](https://eur01.safelinks.protection.outlook.com/?url=https%3A%2F%2Fwww.plos.org%2Fprivacy-policy&data=05%7C02%7CHolly.Carter%40ukhsa.gov.uk%7C9febfee3ee3b41091a0608dc27bd474b%7Cee4e14994a354b2ead475f3cf9de8666%7C0%7C0%7C638428941130855240%7CUnknown%7CTWFpbGZsb3d8eyJWIjoiMC4wLjAwMDAiLCJQIjoiV2luMzIiLCJBTiI6Ik1haWwiLCJXVCI6Mn0%3D%7C0%7C%7C%7C&sdata=YeaODpoF4CABcvafWPO%2BX2JWF1vkbgHUHmzEOR%2BL1tI%3D&reserved=0).

Reviewer #1: **Yes:** Chian Jones Ritten

Reviewer #2: **Yes:** Daniel Pan

Reviewer #3: **Yes:** Dr Sarah Markham
