# Supplementary material for: Effect of face-covering use on adherence to other COVID-19 protective behaviours: A systematic review
Source: PLoS One. 2024 Apr 11;19(4):e0284629. doi: 10.1371/journal.pone.0284629 (PMC11008824; doi:10.1371/journal.pone.0284629)
Supplement: Peer review history

# Peer review: Decision Letter 0

**DOI:** 10.1371/journal.pone.0284629.r001

**Authors:** Rebecca F. Baggaley

20 Jul 2023

PONE-D-23-08549Effect of face-covering use on adherence to other COVID-19 protective behaviours: a systematic reviewPLOS ONE

Dear Dr. Millest,

Thank you for submitting your manuscript to PLOS ONE. After careful consideration, we feel that it has merit but does not fully meet PLOS ONE’s publication criteria as it currently stands. Therefore, we invite you to submit a revised version of the manuscript that addresses the points raised during the review process. Please address all recommendations from reviewers, particularly with regard to study inclusion and appropriate evaluation of study quality and potential biases. Please also respond to Reviewer #2's suggestion to perform a meta-analysis, either by including such an analysis or by providing a satisfactory defence, if you feel that this is not necessary.

Please submit your revised manuscript by Sep 03 2023 11:59PM. If you will need more time than this to complete your revisions, please reply to this message or contact the journal office at plosone@plos.org. When you're ready to submit your revision, log on to https://www.editorialmanager.com/pone/ and select the 'Submissions Needing Revision' folder to locate your manuscript file.

Please include the following items when submitting your revised manuscript:

- A rebuttal letter that responds to each point raised by the academic editor and reviewer(s). You should upload this letter as a separate file labeled 'Response to Reviewers'.
- A marked-up copy of your manuscript that highlights changes made to the original version. You should upload this as a separate file labeled 'Revised Manuscript with Track Changes'.
- An unmarked version of your revised paper without tracked changes. You should upload this as a separate file labeled 'Manuscript'.

If you would like to make changes to your financial disclosure, please include your updated statement in your cover letter. Guidelines for resubmitting your figure files are available below the reviewer comments at the end of this letter.

If applicable, we recommend that you deposit your laboratory protocols in protocols.io to enhance the reproducibility of your results. Protocols.io assigns your protocol its own identifier (DOI) so that it can be cited independently in the future. For instructions see: https://journals.plos.org/plosone/s/submission-guidelines#loc-laboratory-protocols. Additionally, PLOS ONE offers an option for publishing peer-reviewed Lab Protocol articles, which describe protocols hosted on protocols.io. Read more information on sharing protocols at https://plos.org/protocols?utm_medium=editorial-email&utm_source=authorletters&utm_campaign=protocols.

We look forward to receiving your revised manuscript.

Kind regards,

Rebecca F. Baggaley

Academic Editor

PLOS ONE

Journal Requirements:

When submitting your revision, we need you to address these additional requirements.

1. Please ensure that your manuscript meets PLOS ONE's style requirements, including those for file naming. The PLOS ONE style templates can be found at

https://journals.plos.org/plosone/s/file?id=wjVg/PLOSOne_formatting_sample_main_body.pdf and

https://journals.plos.org/plosone/s/file?id=ba62/PLOSOne_formatting_sample_title_authors_affiliations.pdf

2. We note that the grant information you provided in the ‘Funding Information’ and ‘Financial Disclosure’ sections do not match.

When you resubmit, please ensure that you provide the correct grant numbers for the awards you received for your study in the ‘Funding Information’ section.

3. Thank you for stating the following financial disclosure:

“The authors received no specific funding for this work.

HC and CS are supported by the National Institute for Health Research Health Protection Research Units (NIHR HPRU) in Emergency Preparedness and Response, a partnership between UK Health Security Agency (UKHSA), King's College London and the University of East Anglia. The views expressed are those of the authors and not necessarily those of the NIHR, UKHSA or other government departments”

At this time, please address the following queries:

a)        Please clarify the sources of funding (financial or material support) for your study. List the grants or organizations that supported your study, including funding received from your institution.

b)        State what role the funders took in the study. If the funders had no role in your study, please state: “The funders had no role in study design, data collection and analysis, decision to publish, or preparation of the manuscript.”

c)        If any authors received a salary from any of your funders, please state which authors and which funders.

d)        If you did not receive any funding for this study, please state: “The authors received no specific funding for this work.”

Please include your amended statements within your cover letter; we will change the online submission form on your behalf.

4. In your Data Availability statement, you have not specified where the minimal data set underlying the results described in your manuscript can be found. PLOS defines a study's minimal data set as the underlying data used to reach the conclusions drawn in the manuscript and any additional data required to replicate the reported study findings in their entirety. All PLOS journals require that the minimal data set be made fully available. For more information about our data policy, please see http://journals.plos.org/plosone/s/data-availability.

Upon re-submitting your revised manuscript, please upload your study’s minimal underlying data set as either Supporting Information files or to a stable, public repository and include the relevant URLs, DOIs, or accession numbers within your revised cover letter. For a list of acceptable repositories, please see http://journals.plos.org/plosone/s/data-availability#loc-recommended-repositories. Any potentially identifying patient information must be fully anonymized.

Important: If there are ethical or legal restrictions to sharing your data publicly, please explain these restrictions in detail. Please see our guidelines for more information on what we consider unacceptable restrictions to publicly sharing data: http://journals.plos.org/plosone/s/data-availability#loc-unacceptable-data-access-restrictions. Note that it is not acceptable for the authors to be the sole named individuals responsible for ensuring data access.

We will update your Data Availability statement to reflect the information you provide in your cover letter.

5. We note that this manuscript is a systematic review or meta-analysis; our author guidelines therefore require that you use PRISMA guidance to help improve reporting quality of this type of study. Please upload copies of the completed PRISMA checklist as Supporting Information with a file name “PRISMA checklist”.

[Note: HTML markup is below. Please do not edit.]

Reviewers' comments:

Reviewer's Responses to Questions

**Comments to the Author**

1. Is the manuscript technically sound, and do the data support the conclusions?

The manuscript must describe a technically sound piece of scientific research with data that supports the conclusions. Experiments must have been conducted rigorously, with appropriate controls, replication, and sample sizes. The conclusions must be drawn appropriately based on the data presented. 

Reviewer #1: Partly

Reviewer #2: Partly

**********

2. Has the statistical analysis been performed appropriately and rigorously? 

Reviewer #1: Yes

Reviewer #2: N/A

**********

3. Have the authors made all data underlying the findings in their manuscript fully available?

The PLOS Data policy requires authors to make all data underlying the findings described in their manuscript fully available without restriction, with rare exception (please refer to the Data Availability Statement in the manuscript PDF file). The data should be provided as part of the manuscript or its supporting information, or deposited to a public repository. For example, in addition to summary statistics, the data points behind means, medians and variance measures should be available. If there are restrictions on publicly sharing data—e.g. participant privacy or use of data from a third party—those must be specified.

Reviewer #1: Yes

Reviewer #2: Yes

**********

4. Is the manuscript presented in an intelligible fashion and written in standard English?

PLOS ONE does not copyedit accepted manuscripts, so the language in submitted articles must be clear, correct, and unambiguous. Any typographical or grammatical errors should be corrected at revision, so please note any specific errors here.

Reviewer #1: No

Reviewer #2: Yes

**********

5. Review Comments to the Author

Please use the space provided to explain your answers to the questions above. You may also include additional comments for the author, including concerns about dual publication, research ethics, or publication ethics. (Please upload your review as an attachment if it exceeds 20,000 characters)

Reviewer #1: I enjoyed reading, “Effect of face-covering use on adherence to other COVID-19 protective behaviours: a systematic review”. I think the topic is of broad interest, as a systematic review of the potential for face masking to create the unintended consequence of decreased adherence to other COVID-related safety measures, is very important. With some changes, I think the manuscript could make an important contribution to the literature.

Below are my comments on the manuscript:

There are many relevant studies that I believe should be included in the background section to justify the need to study risk compensation behavior in this context:

Include the theory that predicts this risk compensation behavior (and references). I think references to Risk Compensation Theory and the Peltzman Effects should be included.

Include studies focusing on risk compensation related to other covid policies. These studies can provide more of framework for why risk compensation is believed to exist for face masking. Here are a few references I suggest including:

https://doi.org/10.1016/j.cresp.2023.100091

https://doi.org/10.1093/pnasnexus/pgac247

http://dx.doi.org/10.1136/postgradmedj-2021-140234

https://doi.org/10.7326/M20-8251

https://doi.org/10.1371/journal.pone.0279347

There are key details of the studies reported on that I believe should be included. For instance, it is unclear whether face masking is required in most the studies or not. For people who self-select into masking, they may generally adhere more to covid-safety measures than those who do not select in to wearing a mask. So those wearing masks may stay further away from those who do not choose to wear a mask. But it may be the case that those who self-select into wearing masks (even though they are adhere more to social distancing than those not opting into masks) may even choose a larger distance if they were not wearing a mask at all – suggesting risk compensation behavior. I think this type of element needs to at least be mentioned prior to the very end of the manuscript. It could be used to explain the results of many of these papers, and not reject risk compensation behavior.

Be consistent with writing out numbers. Either follow the rule that <10 is written out, or once one number is written out, then all other numbers are written out in that sentence.

Line 26: The beginning of the sentence is written twice

Line 31: The first part of the sentence is redundant to the previous paragraph

Lines 36-48: This should be reduced to one paragraph with more focus on the theory of risk compensation and the Peltzman effect. Further, these paragraphs are very repetitive and could benefit from tightening of the language.

Lines 51-52: I suggest deleting the statement “assess available evidence relating to the effect of face covering use on adherence to other COVID-19 protective behaviours” since it does not add any new information

Lines 52-54: But, this review does not make suggestions on “if and when it is appropriate to recommend or mandate face covering use during future infectious disease outbreaks.” So, I suggest removing this statement.

Lines 59-64: Since the protocol (and its amendments) are accessible at the available link, I don’t think these statements are needed in the main text.

Figure 1: This figure, although informative, does not include the ‘stages’ that are indicated in the text. Further, it does not explain what the stage of ‘title and abstract screening’ means. Does this mean that only the title and abstract were initially reviewed to determine if a paper was to be included in this review? If so, this needs more explanation.

Line 96: Extra period.

Lines 103-107: Is this information needed in the main text? I don’t think so.

Lines 111-127: The quality assessment that is conducted seems out of place since it is not used at all in the review analysis. Why not use the differing levels of quality from this assessment to conduct an additional analysis in the review? For example, do all low-quality papers find similar results, which are different than high-quality papers?

Lines 124-127: I don’t know if this is needed in the main text. I suggest moving it to an appendix.

Line 138: This sentence is unclear.

Lines 152-153: It is unclear what this statement means: “face covering wearing on behalf of oneself and another person with whom one is interacting or is in proximity …”

Lines 164-171: I don’t think is paragraph is needed and suggest deleting it.

Lines 236-237: Compared to who? Is this result found in comparison to those who reported not wearing a mask?

Discussion section: Many statements are made about the strengths and weakness of the stated approaches without use of references.

Lines 312-315: This statement relates to my comments about self-selecting into masking. I think this is one huge advantage of lab experiments that should be more clearly explained.

Lines 463-484: Good inclusion of recommendations for future research!

Reviewer #2: Thank you for the opportunity to review this paper. Millest and colleagues aimed to perform a systematic review, investigating the effect of face covering use on adherence to other COVID-19 related behaviours. They found essentially that there is no evidence that face covering use had any effect on other related behaviours, but that the quality of the evidence is still poor. Generally the manuscript is well written, comprehensive and almost feels like reading a thesis, given its multiple sections and subsections.

My major comment is the distinct lack of meta-analysis, which to me is a shame. The authors clearly point out trends in individual studies, along with conflicting data from different studies. A meta-analysis of these studies (seperated by type of study, and type of intervention for example) would really help crystallise the conclusions that the authors draw. For example, face coverings might reduce face-touching and increase mobility from a forest plot, even if individual studies conflict on this. Trends towards significance might also be important here, along with presentation of a heterogeneity statistic.

Abstract:

Minor comments

- I would suggest including some statistics (OR/p values) in your abstract, for the meta-analysed data on how face covering use might reduce face-touching and increase mobility even if the trend is not significant.

Introduction

Minor comments

- 'prior to the introduction of the vaccination' is typed twice.

Results

Major comments - I am surprised that no meta-analysis of effects was done. This to me makes the results very confusing. For example in section 3.1, there were four observational studies that found a positive or neutral relationship between face covering use or face covering policy and physical distancing; individually, three found no significant relationship between these two variables whilst a fourth one did. Surely this justifies a meta-analysis that takes into account the sample size and thus can present an overall effect?

Similarly for mobility - the 10 natural experiments had conflicting results. Why not meta-analyse these studies? Whilst I acknowledge significant heterogeneity, I do think it is still worth doing an overall analysis, which would help support statements that the authors make in their discussion.

Minor comments - Sections 3.1: it is unusual to write in manuscripts: 'note that in the paragraph that follows..' - I think this could be covered in methods in a more 'reportive' way. 'We found that some papers included more than one methodology in their study and thus, the number of studies described for each methodology may exceed the total number of studies'.

I think the last paragraph of the first section is unnecessary.

Discussion

The authors make multiple clear statements; which were not present in their abstract and in my opinion, isn't necessarily supported by their results, given the lack of meta-analysis. For example - the authors mention in the first paragraph in 4.1 that the majority of lab experiments found that physical distancing was lower in conditions of face covering use, whereas the majority of field experiments found that physical distancing was greater or unchanged. This clearly deserves meta-analysis (seperately) in my opinion to support this conclusion.

**********

6. PLOS authors have the option to publish the peer review history of their article (what does this mean?). If published, this will include your full peer review and any attached files.

If you choose “no”, your identity will remain anonymous but your review may still be made public.

**Do you want your identity to be public for this peer review?** For information about this choice, including consent withdrawal, please see our Privacy Policy.

Reviewer #1: **Yes: **Chian Jones Ritten

Reviewer #2: **Yes: **Daniel Pan

**********

[NOTE: If reviewer comments were submitted as an attachment file, they will be attached to this email and accessible via the submission site. Please log into your account, locate the manuscript record, and check for the action link "View Attachments". If this link does not appear, there are no attachment files.]

While revising your submission, please upload your figure files to the Preflight Analysis and Conversion Engine (PACE) digital diagnostic tool, https://pacev2.apexcovantage.com/. PACE helps ensure that figures meet PLOS requirements. To use PACE, you must first register as a user. Registration is free. Then, login and navigate to the UPLOAD tab, where you will find detailed instructions on how to use the tool. If you encounter any issues or have any questions when using PACE, please email PLOS at figures@plos.org. Please note that Supporting Information files do not need this step.

# Author comment: Author response to Decision Letter 0

**DOI:** 10.1371/journal.pone.0284629.r002

4 Sep 2023

These responses are available in the uploaded file 'Response to Reviewers'

Attachment Submitted filename: Response to Reviewers.docx

# Peer review: Decision Letter 1

**DOI:** 10.1371/journal.pone.0284629.r003

**Authors:** Rebecca F. Baggaley

1 Feb 2024

PONE-D-23-08549R1Effect of face-covering use on adherence to other COVID-19 protective behaviours: a systematic reviewPLOS ONE

Dear Dr. Carter,

Thank you for submitting your manuscript to PLOS ONE. After careful consideration, we feel that it has merit but does not fully meet PLOS ONE’s publication criteria as it currently stands. Therefore, we invite you to submit a revised version of the manuscript that addresses the points raised during the review process.

Please submit your revised manuscript by Mar 17 2024 11:59PM. If you will need more time than this to complete your revisions, please reply to this message or contact the journal office at plosone@plos.org. When you're ready to submit your revision, log on to https://www.editorialmanager.com/pone/ and select the 'Submissions Needing Revision' folder to locate your manuscript file.

Please include the following items when submitting your revised manuscript:

- A rebuttal letter that responds to each point raised by the academic editor and reviewer(s). You should upload this letter as a separate file labeled 'Response to Reviewers'.
- A marked-up copy of your manuscript that highlights changes made to the original version. You should upload this as a separate file labeled 'Revised Manuscript with Track Changes'.
- An unmarked version of your revised paper without tracked changes. You should upload this as a separate file labeled 'Manuscript'.

If you would like to make changes to your financial disclosure, please include your updated statement in your cover letter. Guidelines for resubmitting your figure files are available below the reviewer comments at the end of this letter.

If applicable, we recommend that you deposit your laboratory protocols in protocols.io to enhance the reproducibility of your results. Protocols.io assigns your protocol its own identifier (DOI) so that it can be cited independently in the future. For instructions see: https://journals.plos.org/plosone/s/submission-guidelines#loc-laboratory-protocols. Additionally, PLOS ONE offers an option for publishing peer-reviewed Lab Protocol articles, which describe protocols hosted on protocols.io. Read more information on sharing protocols at https://plos.org/protocols?utm_medium=editorial-email&utm_source=authorletters&utm_campaign=protocols.

We look forward to receiving your revised manuscript.

Kind regards,

Rebecca F. Baggaley

Academic Editor

PLOS ONE

Journal Requirements:

Please review your reference list to ensure that it is complete and correct. If you have cited papers that have been retracted, please include the rationale for doing so in the manuscript text, or remove these references and replace them with relevant current references. Any changes to the reference list should be mentioned in the rebuttal letter that accompanies your revised manuscript. If you need to cite a retracted article, indicate the article’s retracted status in the References list and also include a citation and full reference for the retraction notice.

[Note: HTML markup is below. Please do not edit.]

Reviewers' comments:

Reviewer's Responses to Questions

**Comments to the Author**

1. If the authors have adequately addressed your comments raised in a previous round of review and you feel that this manuscript is now acceptable for publication, you may indicate that here to bypass the “Comments to the Author” section, enter your conflict of interest statement in the “Confidential to Editor” section, and submit your "Accept" recommendation.

Reviewer #1: (No Response)

Reviewer #2: (No Response)

Reviewer #3: All comments have been addressed

**********

2. Is the manuscript technically sound, and do the data support the conclusions?

The manuscript must describe a technically sound piece of scientific research with data that supports the conclusions. Experiments must have been conducted rigorously, with appropriate controls, replication, and sample sizes. The conclusions must be drawn appropriately based on the data presented. 

Reviewer #1: Yes

Reviewer #2: Partly

Reviewer #3: Yes

**********

3. Has the statistical analysis been performed appropriately and rigorously? 

Reviewer #1: N/A

Reviewer #2: No

Reviewer #3: N/A

**********

4. Have the authors made all data underlying the findings in their manuscript fully available?

The PLOS Data policy requires authors to make all data underlying the findings described in their manuscript fully available without restriction, with rare exception (please refer to the Data Availability Statement in the manuscript PDF file). The data should be provided as part of the manuscript or its supporting information, or deposited to a public repository. For example, in addition to summary statistics, the data points behind means, medians and variance measures should be available. If there are restrictions on publicly sharing data—e.g. participant privacy or use of data from a third party—those must be specified.

Reviewer #1: Yes

Reviewer #2: Yes

Reviewer #3: Yes

**********

5. Is the manuscript presented in an intelligible fashion and written in standard English?

PLOS ONE does not copyedit accepted manuscripts, so the language in submitted articles must be clear, correct, and unambiguous. Any typographical or grammatical errors should be corrected at revision, so please note any specific errors here.

Reviewer #1: No

Reviewer #2: Yes

Reviewer #3: Yes

**********

6. Review Comments to the Author

Please use the space provided to explain your answers to the questions above. You may also include additional comments for the author, including concerns about dual publication, research ethics, or publication ethics. (Please upload your review as an attachment if it exceeds 20,000 characters)

Reviewer #1: I enjoyed reading the revised manuscript, “Effect of face-covering use on adherence to other COVID-19 protective behaviours: a systematic review”. The potential for adverse behavior related to risk compensation from COVID-related protective measures is a very important issue. I believe the changes made improved the quality of the manuscript and highlight the potential issues related to face making. Yet, there are many grammatical and clarification issues I believe should be addressed. I have highlighted my specific comments in the attached manuscript through embedded comments.

Reviewer #2: Thank you for the opportunity to re-review this manuscript. The authors have put much work into this revision.

My main feedback with regards to this work was the lack of meta-analysis of the study effects. The authors, having consulted with colleagues who have performed meta-analyses in the past, have decided to not apply meta-analysis techniques. One of the reasons they provide is the high level of heterogeneity between studies.

It is usual for meta-analysis exploring the field of social science to have high degrees of heterogeneity - in my opinion, this does not preclude pooling of data and has been done in the past. Instead, one can explore heterogeneity through various sensitivity analyses, which provide an important visualisation of the data available. This would, without words, highlight much of the variation the authors discuss in their discussion section, and therefore the need to improve data collection and analyses, including greater standardisation across studies.

The main reason for why I suggested a meta-analyses is that I don't feel like the authors have made the most out of the data that they have synthesised. The authors conclude that the impact of face coverings on behaviour is inconsistent, with some finding and effect whilst others do not - this is clearly going to be impacted by the size of the study and may actually be the incorrect conclusion. Furthermore, the author says that 'there is no clear consensus in the literature as to whether face covering use improves adherence to other protective behaviours...' The authors must at the very least, if they not meta-analysing, discuss more precisely the contexts in which face coverings may change behaviour, and in other circumstances they do not. This again is the point of a data synthesis; if there is already a clear consensus in the literature, why do a data synthesis in the first place?

Reviewer #3: This is a thorough and balanced systematic review regarding the effect of face-covering use on adherence to other COVID-19 protective behaviours, and is of value in informing response to future responses to similar public health threats.

**********

7. PLOS authors have the option to publish the peer review history of their article (what does this mean?). If published, this will include your full peer review and any attached files.

If you choose “no”, your identity will remain anonymous but your review may still be made public.

**Do you want your identity to be public for this peer review?** For information about this choice, including consent withdrawal, please see our Privacy Policy.

Reviewer #1: **Yes: **Chian Jones Ritten

Reviewer #2: **Yes: **Daniel Pan

Reviewer #3: **Yes: **Dr Sarah Markham

**********

[NOTE: If reviewer comments were submitted as an attachment file, they will be attached to this email and accessible via the submission site. Please log into your account, locate the manuscript record, and check for the action link "View Attachments". If this link does not appear, there are no attachment files.]

While revising your submission, please upload your figure files to the Preflight Analysis and Conversion Engine (PACE) digital diagnostic tool, https://pacev2.apexcovantage.com/. PACE helps ensure that figures meet PLOS requirements. To use PACE, you must first register as a user. Registration is free. Then, login and navigate to the UPLOAD tab, where you will find detailed instructions on how to use the tool. If you encounter any issues or have any questions when using PACE, please email PLOS at figures@plos.org. Please note that Supporting Information files do not need this step.

Attachment Submitted filename: PONE-D-23-08549_R1.pdf

# Author comment: Author response to Decision Letter 1

**DOI:** 10.1371/journal.pone.0284629.r004

13 Mar 2024

Reviewers' comments:

Reviewer's Responses to Questions

Comments to the Author

1. If the authors have adequately addressed your comments raised in a previous round of review and you feel that this manuscript is now acceptable for publication, you may indicate that here to bypass the “Comments to the Author” section, enter your conflict of interest statement in the “Confidential to Editor” section, and submit your "Accept" recommendation.

Reviewer #1: (No Response)

Reviewer #2: (No Response)

Reviewer #3: All comments have been addressed

________________________________________

2. Is the manuscript technically sound, and do the data support the conclusions?

The manuscript must describe a technically sound piece of scientific research with data that supports the conclusions. Experiments must have been conducted rigorously, with appropriate controls, replication, and sample sizes. The conclusions must be drawn appropriately based on the data presented. 

Reviewer #1: Yes

Reviewer #2: Partly

Reviewer #3: Yes

________________________________________

3. Has the statistical analysis been performed appropriately and rigorously? 

Reviewer #1: N/A

Reviewer #2: No

Reviewer #3: N/A

________________________________________

4. Have the authors made all data underlying the findings in their manuscript fully available?

The PLOS Data policy requires authors to make all data underlying the findings described in their manuscript fully available without restriction, with rare exception (please refer to the Data Availability Statement in the manuscript PDF file). The data should be provided as part of the manuscript or its supporting information, or deposited to a public repository. For example, in addition to summary statistics, the data points behind means, medians and variance measures should be available. If there are restrictions on publicly sharing data—e.g. participant privacy or use of data from a third party—those must be specified.

Reviewer #1: Yes

Reviewer #2: Yes

Reviewer #3: Yes

________________________________________

5. Is the manuscript presented in an intelligible fashion and written in standard English?

PLOS ONE does not copyedit accepted manuscripts, so the language in submitted articles must be clear, correct, and unambiguous. Any typographical or grammatical errors should be corrected at revision, so please note any specific errors here.

Reviewer #1: No

Reviewer #2: Yes

Reviewer #3: Yes

________________________________________

6. Review Comments to the Author

Please use the space provided to explain your answers to the questions above. You may also include additional comments for the author, including concerns about dual publication, research ethics, or publication ethics. (Please upload your review as an attachment if it exceeds 20,000 characters)

Reviewer #1: I enjoyed reading the revised manuscript, “Effect of face-covering use on adherence to other COVID-19 protective behaviours: a systematic review”. The potential for adverse behavior related to risk compensation from COVID-related protective measures is a very important issue. I believe the changes made improved the quality of the manuscript and highlight the potential issues related to face making. Yet, there are many grammatical and clarification issues I believe should be addressed. I have highlighted my specific comments in the attached manuscript through embedded comments.

We thank the reviewer for their detailed review and helpful suggestions for improving grammar and clarity. We have incorporated all suggested changes and feel that the manuscript is strengthened as a result.

Reviewer #2: Thank you for the opportunity to re-review this manuscript. The authors have put much work into this revision.

My main feedback with regards to this work was the lack of meta-analysis of the study effects. The authors, having consulted with colleagues who have performed meta-analyses in the past, have decided to not apply meta-analysis techniques. One of the reasons they provide is the high level of heterogeneity between studies.

It is usual for meta-analysis exploring the field of social science to have high degrees of heterogeneity - in my opinion, this does not preclude pooling of data and has been done in the past. Instead, one can explore heterogeneity through various sensitivity analyses, which provide an important visualisation of the data available. This would, without words, highlight much of the variation the authors discuss in their discussion section, and therefore the need to improve data collection and analyses, including greater standardisation across studies.

The main reason for why I suggested a meta-analyses is that I don't feel like the authors have made the most out of the data that they have synthesised. The authors conclude that the impact of face coverings on behaviour is inconsistent, with some finding and effect whilst others do not - this is clearly going to be impacted by the size of the study and may actually be the incorrect conclusion. Furthermore, the author says that 'there is no clear consensus in the literature as to whether face covering use improves adherence to other protective behaviours...' The authors must at the very least, if they not meta-analysing, discuss more precisely the contexts in which face coverings may change behaviour, and in other circumstances they do not. This again is the point of a data synthesis; if there is already a clear consensus in the literature, why do a data synthesis in the first place?

We thank the reviewer for taking the time to review our manuscript, and for their considered comments. As previously discussed, and noted by the reviewer, we have decided not to carry out a meta-analysis due to the heterogeneity of the studies included within the review. The Cochrane handbook cautions against the use of meta-analysis when studies have high levels of heterogeneity, due to the increased risk of errors or incorrect conclusions (Cochrane Handbook, Chapter 10: Analysing data and undertaking meta-analyses). In line with this, and in response to one of Reviewer 1’s comments, we have now added the following text to the limitations in section 4.8 to explain our decision not to carry out meta-analysis: “An additional consideration relates to the potential to conduct a meta-analysis of the included studies. Given the heterogeneity of the included studies (both in terms of clinical heterogeneity and methodological heterogeneity), a decision was taken not to carry out a meta-analysis. This is in line with Cochrane guidance on heterogeneity within meta-analysis, which states that meta-analysis should only be undertaken if studies are considered to be sufficiently similar to ensure a meaningful outcome and avoid drawing potentially misleading conclusions. However, as further research is conducted in this area, potentially resulting in more clinically and methodologically similar studies, future studies could build on this review by carrying out a meta-analysis”.

While we have decided not to carry out a meta-analysis, for the reasons outlined above, we agree with Reviewer 2 that a more precise overall discussion of the contexts in which face coverings do, or do not, change behaviour would be beneficial to the reader, and would make the review more impactful. We had previously discussed the overall impact of face covering use in different contexts within each behavioural outcome section of the discussion (sections 4.1 – 4.6). We have now also expanded the overall conclusion to state more precisely the contexts in which face coverings may impact different behaviours, and to highlight for which behaviours the evidence-base allows potential conclusions about the impact of face coverings to be reached, and for which behaviours further research is required:

“For some behavioural outcomes, it is possible to draw tentative conclusions regarding the impact of face coverings on behaviour. For example, evidence suggests that wearing a face covering reduces the amount that an individual touches their face; face coverings may therefore have a positive impact on face touching behaviours. Conversely, evidence suggests that the introduction of face mask mandates may increase mobility; face coverings may therefore have a negative impact on mobility-related behaviours. Some studies suggest that there may be a relationship between wearing a face covering and other protective behaviours (such as increased hand hygiene). However, these studies are predominately cross-sectional and rely on self-report measures; they therefore do not allow conclusions to be drawn about the impact of face covering use on adherence to these behaviours. Evidence relating to the impact of face covering use on physical distancing and close contacts is mixed. Findings are particularly inconsistent in relation to physical distancing, with considerable discrepancy found between study types. Whilst assessments of behaviour in real-life settings tend to find that face covering use either increases or has no impact upon physical distancing, lab experiments tend to suggest that physical distancing decreases in conditions of face covering use. There is some suggestion that the impact of face covering use on physical distancing could be affected by whether or not mandatory face covering policies are in effect (which would indicate that conclusions drawn from a voluntary context should not be directly applied to a mandatory context and vice versa) but further assessment of this potential moderating effect is warranted. Evidence relating to the potential impact of risk compensation is also inconsistent; while some studies suggest risk compensation may play a role in shaping behaviour, others suggest it has no impact. Overall, this review highlights that the impact of face covering use on other protective behaviours is likely to vary based on the behaviour under investigation. While for some behaviours the evidence suggests a positive impact (e.g., face coverings may result in reduced face touching), for other behaviours the evidence suggests a negative impact (e.g., increased mobility), or provides no clear consensus (e.g., physical distancing, close contacts, and adherence to other protective behaviours).” 

Reviewer #3: This is a thorough and balanced systematic review regarding the effect of face-covering use on adherence to other COVID-19 protective behaviours, and is of value in informing response to future responses to similar public health threats.

________________________________________

7. PLOS authors have the option to publish the peer review history of their article (what does this mean?). If published, this will include your full peer review and any attached files.

If you choose “no”, your identity will remain anonymous but your review may still be made public.

Do you want your identity to be public for this peer review? For information about this choice, including consent withdrawal, please see our Privacy Policy.

Reviewer #1: Yes: Chian Jones Ritten

Reviewer #2: Yes: Daniel Pan

Reviewer #3: Yes: Dr Sarah Markham

Attachment Submitted filename: Response to reviewers 13.03.24.docx

# Peer review: Decision Letter 2

**DOI:** 10.1371/journal.pone.0284629.r005

**Authors:** Wondwossen Amogne Degu

27 Mar 2024

Effect of face-covering use on adherence to other COVID-19 protective behaviours: a systematic review

PONE-D-23-08549R2

Dear Dr. Carter,

We’re pleased to inform you that your manuscript has been judged scientifically suitable for publication and will be formally accepted for publication once it meets all outstanding technical requirements.

Within one week, you’ll receive an e-mail detailing the required amendments. When these have been addressed, you’ll receive a formal acceptance letter and your manuscript will be scheduled for publication.

An invoice will be generated when your article is formally accepted. Please note, if your institution has a publishing partnership with PLOS and your article meets the relevant criteria, all or part of your publication costs will be covered. Please make sure your user information is up-to-date by logging into Editorial Manager at Editorial Manager® and clicking the ‘Update My Information' link at the top of the page. If you have any questions relating to publication charges, please contact our Author Billing department directly at authorbilling@plos.org.

If your institution or institutions have a press office, please notify them about your upcoming paper to help maximize its impact. If they’ll be preparing press materials, please inform our press team as soon as possible -- no later than 48 hours after receiving the formal acceptance. Your manuscript will remain under strict press embargo until 2 pm Eastern Time on the date of publication. For more information, please contact onepress@plos.org.

Kind regards,

Wondwossen Amogne Degu, M.D

Academic Editor

PLOS ONE

Additional Editor Comments (optional):

Reviewers' comments:

Reviewer's Responses to Questions

**Comments to the Author**

1. If the authors have adequately addressed your comments raised in a previous round of review and you feel that this manuscript is now acceptable for publication, you may indicate that here to bypass the “Comments to the Author” section, enter your conflict of interest statement in the “Confidential to Editor” section, and submit your "Accept" recommendation.

Reviewer #1: All comments have been addressed

Reviewer #2: All comments have been addressed

Reviewer #3: All comments have been addressed

**********

2. Is the manuscript technically sound, and do the data support the conclusions?

The manuscript must describe a technically sound piece of scientific research with data that supports the conclusions. Experiments must have been conducted rigorously, with appropriate controls, replication, and sample sizes. The conclusions must be drawn appropriately based on the data presented. 

Reviewer #1: Yes

Reviewer #2: Partly

Reviewer #3: Yes

**********

3. Has the statistical analysis been performed appropriately and rigorously? 

Reviewer #1: Yes

Reviewer #2: I Don't Know

Reviewer #3: Yes

**********

4. Have the authors made all data underlying the findings in their manuscript fully available?

The PLOS Data policy requires authors to make all data underlying the findings described in their manuscript fully available without restriction, with rare exception (please refer to the Data Availability Statement in the manuscript PDF file). The data should be provided as part of the manuscript or its supporting information, or deposited to a public repository. For example, in addition to summary statistics, the data points behind means, medians and variance measures should be available. If there are restrictions on publicly sharing data—e.g. participant privacy or use of data from a third party—those must be specified.

Reviewer #1: Yes

Reviewer #2: No

Reviewer #3: Yes

**********

5. Is the manuscript presented in an intelligible fashion and written in standard English?

PLOS ONE does not copyedit accepted manuscripts, so the language in submitted articles must be clear, correct, and unambiguous. Any typographical or grammatical errors should be corrected at revision, so please note any specific errors here.

Reviewer #1: Yes

Reviewer #2: Yes

Reviewer #3: Yes

**********

6. Review Comments to the Author

Please use the space provided to explain your answers to the questions above. You may also include additional comments for the author, including concerns about dual publication, research ethics, or publication ethics. (Please upload your review as an attachment if it exceeds 20,000 characters)

Reviewer #1: Thank you for the opportunity to read the edited manuscript, "Effect of face-covering use on adherence to other COVID-19 protective behaviours: a systematic review". The paper now makes a clear and important contribution to the literature on COVID-19 protective behaviors and risk compensation.

There are still some remaining grammatical errors throughout the manuscript that should be addressed prior to publication.

Reviewer #2: Thank you for the opportunity to once again review this paper. I see that the authors have decided to not meta-analyse, citing the Cochrane Handbook relating to the point about heterogeneity. In my opinion, it is difficult to decide about the value of a meta-analysis prior to performing it. My interpretation of the cited text from the Cochrane Handbook is that interpretation of meta-analysis results should be done with the heterogeneity in mind, rather than choosing to not analyse from the beginning.

Nevertheless the authors have done their very best and addressed the text to justify my comments aside from meta-analysis results. I believe it should now be up to the Editor to decide whether the journal should include this work with or without meta-analysis.

Reviewer #3: I have reviewed and this interesting and clinically relevant paper and recommend it for publication.

**********

7. PLOS authors have the option to publish the peer review history of their article (what does this mean?). If published, this will include your full peer review and any attached files.

If you choose “no”, your identity will remain anonymous but your review may still be made public.

**Do you want your identity to be public for this peer review?** For information about this choice, including consent withdrawal, please see our Privacy Policy.

Reviewer #1: **Yes: **Chian Jones Ritten

Reviewer #2: **Yes: **Daniel Pan

Reviewer #3: No

**********

# Editor report: Acceptance letter

**DOI:** 10.1371/journal.pone.0284629.r006

**Authors:** Wondwossen Amogne Degu

1 Apr 2024

PONE-D-23-08549R2 

PLOS ONE

Dear Dr. Carter, 

I'm pleased to inform you that your manuscript has been deemed suitable for publication in PLOS ONE. Congratulations! Your manuscript is now being handed over to our production team.

At this stage, our production department will prepare your paper for publication. This includes ensuring the following:

* All references, tables, and figures are properly cited

* All relevant supporting information is included in the manuscript submission,

* There are no issues that prevent the paper from being properly typeset

If revisions are needed, the production department will contact you directly to resolve them. If no revisions are needed, you will receive an email when the publication date has been set. At this time, we do not offer pre-publication proofs to authors during production of the accepted work. Please keep in mind that we are working through a large volume of accepted articles, so please give us a few weeks to review your paper and let you know the next and final steps. 

Lastly, if your institution or institutions have a press office, please let them know about your upcoming paper now to help maximize its impact. If they'll be preparing press materials, please inform our press team within the next 48 hours. Your manuscript will remain under strict press embargo until 2 pm Eastern Time on the date of publication. For more information, please contact onepress@plos.org.

If we can help with anything else, please email us at customercare@plos.org.

Thank you for submitting your work to PLOS ONE and supporting open access. 

Kind regards, 

PLOS ONE Editorial Office Staff

on behalf of

Dr. Wondwossen Amogne Degu 

Academic Editor

PLOS ONE
